# A new hybrid optimization approach using PSO, Nelder-Mead Simplex and Kmeans clustering algorithms for 1D Full Waveform Inversion

**DOI:** 10.1371/journal.pone.0277900

**Published:** 2022-12-14

**Authors:** Rutinaldo Aguiar Nascimento, Álvaro Barroca Neto, Yuri Shalom de Freitas Bezerra, Hugo Alexandre Dantas do Nascimento, Liacir dos Santos Lucena, Joaquim Elias de Freitas

**Affiliations:** 1 Secretaria de Estado da Educação, da Cultura, do Esporte e do Lazer do Rio Grande do Norte, Natal, RN, Brazil; 2 Programa de Pós-graduação em Ciência e Engenharia de Petróleo, Universidade Federal do Rio Grande do Norte, Natal, RN, Brazil; 3 Instituto de Informática, Universidade Federal de Goiás, Goiânia, GO, Brazil; Torrens University Australia, AUSTRALIA

## Abstract

The FWI is formulated as a nonlinear optimization problem that traditionally uses local (derivative-based) minimization to find the scalar field of properties that best represents the field seismic data. This problem has a high computational cost and accuracy limited to local minima, in addition to suffering from a slow convergence rate (Cycle Skipping). Therefore, we developed a two-phase hybrid optimization algorithm based on DFO algorithms. The first use global minimization and clustering technique. The second use local minimization. In phase 1 we adopted the modified PSO and K-means algorithms and in phase 2, we adopted the ANMS. We call the hybrid algorithm of the PSO-Kmeans-ANMS. Where K-means is responsible for dividing swarms of particles into 2 clusters at every instant. This strategy aims to automatically balance the mechanisms of exploration and exploitation of the parameter search space by the hybrid algorithm, allowing one to find more precise solutions and consequently improving its convergence. The PSO-Kmeans-ANMS algorithm was validated on the set of 12 benchmark functions and applied to the FWI 1D problem. We compared PSO-Kmeans-ANMS with classic PSO, modified PSO, and ANMS algorithms. The metrics used were are the average execution time and the success rate (an error of ± 4% of the optimal solution). In all validation experiments and the FWI application, the PSO-Kmeans-ANMS performed well in terms of robustness and computational efficiency. In the case of FWI, there was a significant reduction in computational cost, thus presenting a relevant result.

## Introduction

In the oil industry, the seismic reflection method is the most used in the prospection of hydrocarbon deposits, as it allows imaging the structures and geological layers of the subsurface regions based on the behavior of seismic waves in relation to the different petrophysical properties of the rocks, at a relatively low cost.

The imaging is performed through the application of a seismic data inversion technique. This technique occurs through an iterative inversion process, which uses an optimization method to minimize an objective function. This function quantifies the misfit between the data observed in the seismic exploration and those calculated in the modeling. In this way, making the most of the information available in the data, which includes the phase, amplitude, and transit time of the seismic wave field, Tarantola [1].

In the last decade, Full Waveform Inversion (FWI) has become one of the most powerful techniques for inversion of seismic data. This technique is capable of estimating with high precision the elastic parameters of the velocity model that describes, with the best possible approximation, the observed data. That is, it can find the smallest possible misfit between observed and calculated data.

Traditionally, FWI uses the adjoint operator method, according to Plessix [2], based on an optimization strategy with derivatives, such as gradient descent, conjugate gradient, and quasi-Newton. However, these approaches have a high computational cost with an accuracy limited to local minima, thus requiring an initial model close enough to the global optimum so that it can converge to at least an approximate solution, Datta *et al.* [3].

Therefore, many strategies have been proposed to deal with the mentioned problems, and recently, hybrid optimization algorithms were applied to FWI to make the cost function minimization process more efficient and accurate, in the search for the global minimum.

Hybrid search algorithms have the potential to combine global and local optimization techniques. Such algorithms do not require a good initial model to obtain good results and have a low computational cost, compared to global optimization algorithms, Chunduru *et al.* [4].

In this work, we develop a new two-phase optimization hybrid algorithm, composed of the modified PSO, K-means, and ANMS algorithms. The hybrid algorithm, here called PSO-Kmeans-ANMS, was built with a new approach that, in Phase 1, consists of the global search by PSO and the clustering of the swarm of particles by K-means. This last one is used to divide the swarm into two clusters at each iteration. When one of the clusters becomes dominant, in size, or the swarm appears homogeneous, according to the metric of the standard deviation between the values of the swarm’s objective function, Phase 1 of the hybrid algorithm ends. When there is a solution close to the global minimum, Phase 2 begins, which consists of the assertiveness of the local search by ANMS. Therefore, the specific objective of the study is the commitment to obtaining accurate solutions with fewer evaluations of the objective function. Therefore, we aim to reduce the computational cost for the optimization problem with a complex and costly misfit function, as in the case of the FWI. The results of this research show that these objectives were achieved by the proposed hybrid algorithm compared to the classic PSO, modified PSO, and ANMS algorithms.

For validation and understanding, we applied the proposed hybrid algorithm to 12 benchmark functions. Convergence performance was measured by success rate and average execution time. The qualitative results of the new algorithm are compared to those obtained with each of the classic PSO, modified PSO, and ANMS algorithms.

The rest of this article is organized as follows: Theory, Validation with Benchmark Functions, and 1D FWI Application. The Theory section presents all aspects related to the FWI problem, 1D Seismic problem modeling, Numerical solution of the wave equation, description of the DFO algorithms, K-means Clustering Algorithm, Hilbert Curve, and description of our hybrid approach.

## Related literature

In an attempt to improve the performance of non-linear optimization, new variants of the PSO have been continuously developed and successfully implemented in the most diverse areas of research. One is hybrid optimization, which combines PSO strategies with other types of optimizers. For instance, Fan, Liang and Zahara [5] proposed a hybrid approach using the Nelder-Mead Simplex and PSO algorithms for the optimization of multimodal functions. Its main advantage is its easy numerical implementation. A set of 17 test functions showed that the hybrid approach was superior to the two algorithms that make it up, separately, in terms of solution quality and convergence rate. In addition, the referred hybrid method was also compared to eight other published methods, such as a hybrid genetic algorithm (GA), a continuous GA, a simulated annealing (SA) and a tabu search (TS). Overall, the new approach proved to be extremely effective and efficient in finding ideal solutions for applications with multimodal functions.

Rana, Jasola and Kuma [6] proposed a hybrid sequential cluster approach, using a PSO in sequence with a K-means algorithm, for data clustering. In that approach, the PSO was also employed to find the cluster center before a K-means clustering. The new approach overcame the drawbacks of both algorithms, improved clustering and avoided being trapped in local solution. Experiments with four different data sets compared K-means and PSO alone against the hybrid K-means combined with PSO and with GA. The results showed that the proposed algorithm generates more accurate, robust and better cluster results.

Koduru, Das and Welch [7] also presented a hybrid algorithm using the Nelder-Mead Simplex and the PSO, but with a different approach, including the K-means clustering algorithm. They analyzed the two hybrid algorithms with and without K-means clustering and showed that both approaches led to a significant acceleration in the convergence rate for several well-known reference problems, as well as for the problem of fitting a gene model with observable data. Firouzi, Sadeghi and Niknam [8] proposed a hybrid algorithm similar to the previous idea but using PSO, SA and K-means algorithms to find a better cluster partition. The efficiency of the proposed clustering technique was evaluated with several sets of reference data, showing that the proposed algorithm surpasses many others methods such as PSO, SA, Ant Colony Optimization (ACO), GA, TS, Honey Bee Mating Optimization (HBMO), Nelder-Mead Simplex and K-means for partial clustering problems. The efficiency is evaluated form other hybrids algorithms such as the combination of PSO and SA, combination of K-means and PSO, combination of Nelder-Mead Simplex and PSO, and other similar to Koduru, Das and Welch [7].

Nayak *et al.* [9] proposed a hybridization between improved PSO (IPSO) and GA algorithms, together with the K-means clustering algorithm, to help convergence. In the first stage, the IPSO was used to obtain a global solution among the best cluster centers. Then, GA crossing sequences were employed to improve the quality of the models, while mutation was applied to diversify the search in the solution space, therefore avoiding premature convergence. The performance of the proposed hybrid algorithm was compared to other existing clustering techniques like the K-means alone and to hybrid combinations between K-means with PSO and with GA.

Perumal and Dilip [10] proposed a hybrid method using the Gravitational Search Algorithm (GSA), K-means, Nelder-Mead Simplex and PSO algorithms. The method helped the K-means to escape from local optima and also increased the convergence rate. The authors specialized their algorithm for improving the cluster centers on arbitrary data sets.

More recently, Fakhouri, Hudaib and Sleit [11] proposed a hybrid algorithm using the PSO, Sine-Cosine Algorithm (SCA) and the Nelder-Mead Simplex. The performance of the new algorithm on a set of 23 known unimodal and multimodal functions was higher than that obtained by the PSO and by other more advanced algorithms. The hybrid algorithm was also tested for solving an engineering design problem, such as spring compression and welded beam. This demonstrated that, in engineering application problems, the proposed algorithm has a good response and can be used in difficult cases, involving unknown research areas.

Other approaches have been successful in increasing the convergence rate by improving the diversity of solutions. According tot [12], the improved gray wolf optimization (I-GWO) algorithm introduces a search strategy named dimension learning-based hunting (DLH) inspired by the individual hunting of gray wolves. This selects the candidate from the GWO or DLH search strategies based on the quality of the new solutions. The cooperation between these two search strategies improves the global and local search capability of the algorithm. Similar to [11], I-GWO has been tested on 29 reference functions and a variety of engineering design problems. It presented excellent results, both in terms of precision and convergence rate. Thus, the use of improved versions of known algorithms should be an inspiration in the development of more effective hybrid algorithms.

## Theory

In this section, we briefly describe the FWI, the equations that govern the inversion problem and its numerical solution, as well as the theoretical aspects of the optimization algorithms PSO classic, PSO modified, ANMS and K-means also a brief introduction to the Hilbert curve. We conclude with the design and operational procedure of our new hybrid algorithm.

### Full waveform inversion

In the one dimensional seismic inversion problems, such as the FWI, we usually define the vectors with the seismic data **d** and the model parameters (or the model itself) **m**, where **d** ∈ ℜ^*T*^ and **m** ∈ ℜ^*N*^. A mathematical operator *G* relates **m** to **d**. The equation associated to this problem is given by

$$\begin{array}{c} G(m)=d. \end{array}$$ (1)

The equation above represents the direct problem (also called forward modeling), in which, given **m** and **G**, we get **d**, by applying the linear system **Gm** = d*, where d* is calculated data. The inverse problem aims to find **m**, by having **d** and **G**^−1^ applying the same equation in inverse form, **G**^−1^**d**^*obs*^ = **m**, where **d**^*obs*^ is observed data.

For FWI, the direct operator **G** is composed by the seismic wave equation and its restrictions for the referred problem, that is, the initial and the boundary conditions. Unfortunately, the direct inversion of the system in Eq (1) is not possible in most cases, because analytical solutions of the seismic wave equation become too complex and are usually limited to simple problems [13]. Nevertheless, any inverse problem can be posed as an optimization problem and the FWI is one of the most nonlinear inversion problems [14]. The most common objective function used in inverse theory is the least squares function, based on the *l*_2_ norm. This aims to quantify the misfit between the calculated and the observed data in a smooth way. The best fit of the calculated data to the observed one is the associated one with the minimum value of the misfit function given below [15]

$$\begin{array}{c} \phi \left(m\right)=\frac{1}{2}{∥d^{obs}-d^{*}∥}_{2}^{2}. \end{array}$$ (2)

In this way, the FWI problem is naturally formulated as a nonlinear optimization problem of the form

$$\begin{array}{c} \text{minimize}\;\phi (m)\\ \text{subject}\;\text{to}\;Gm=d^{*}. \end{array}$$

The FWI can be seen as an optimization problem that starts with an initial guessed solution (an initial model) and iteratively improves it in order to obtain a solution that is more compatible with the observed data. This is done by repeating a sequence of steps such as: performing a forward modeling, evaluating an objective function, computing an improvement direction and updating the current model. The forward modeling solves the wave equation by simulating the wave propagation in a physical medium, in this case, a model of the physical properties of this medium. Traditionally, FWI is based on the adjoint operator method which employs derivatives. In that approach, for each iteration, a search direction and a step length is calculated to update the model [1]. The general formula can be stated as

$$\begin{array}{c} m^{k+1}=m^{k}-\alpha_{h}h^{k}, \end{array}$$ (3)

where *k* is the number of iterations, *α*_*h*_ denotes the step length in relation to **h**, which denotes the search direction. For the steepest descent method, the search direction is equal to the gradient of the misfit function, $h=g=\frac{\partial }{\partial m}\phi \left(m\right)$. For the Gauss-Newton method, the search direction is **h** = **H**^−1^**g**, where **H** is the Hessian matrix of the misfit function [16] and it is computationally much more expensive to be calculated.

**Fig 1:**
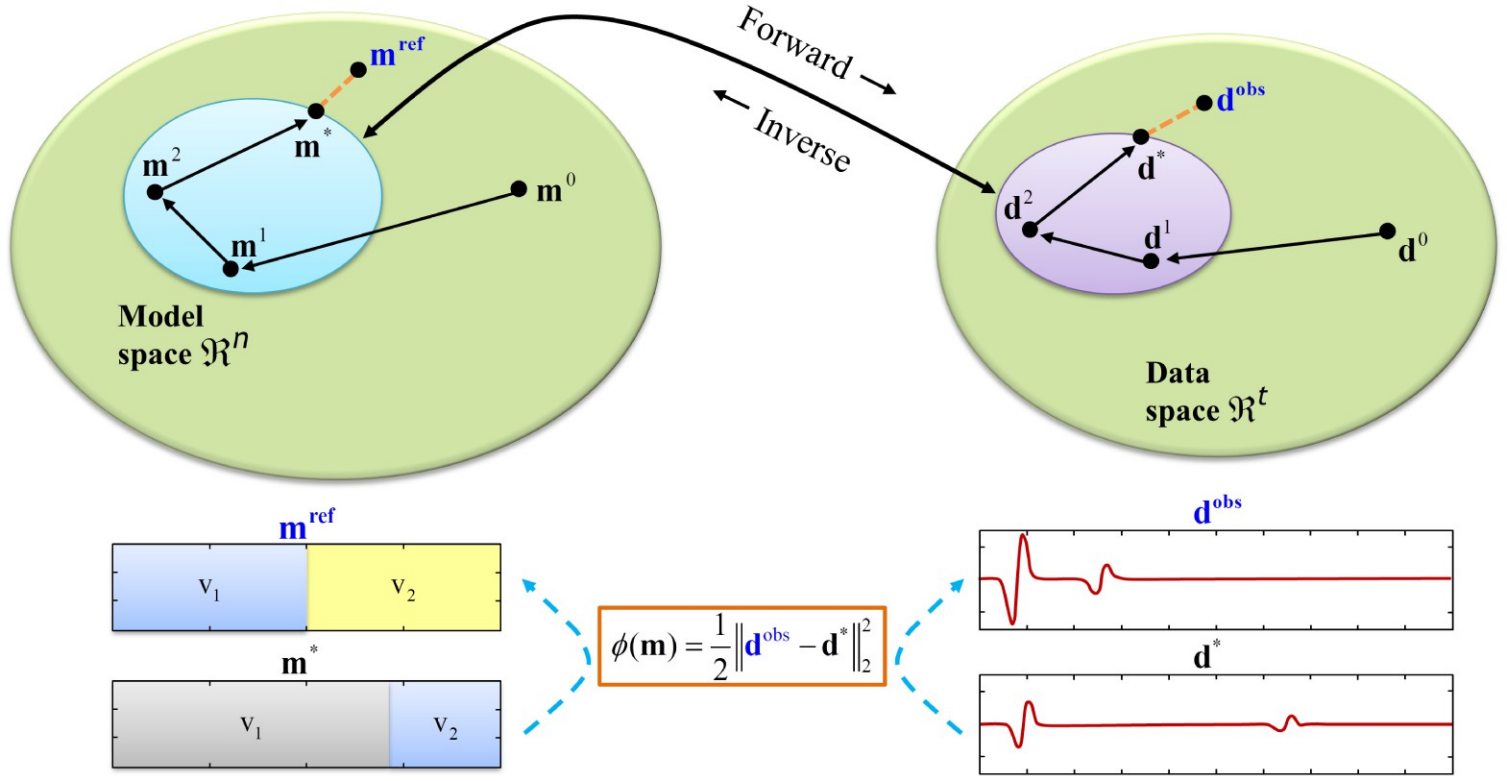
Forward and inverse problems and misfit function. The FWI iterative process solves the inverse seismic problem by successively applying the direct seismic problem, fitting the model to the data, until the misfit is minimal.

Fig 1 shows schematically how the FWI works in the iterative updating of an initial model **m**^0^ to an estimated model m* until the misfit between the calculated data d* and observed data **d**^*obs*^ is minimal. That is, it searches for the minimum of *ϕ*(**m**), given by Eq (2). The idea is to walk iteratively through the model space, ℜ^*N*^, in such a way that m* gets closer to **m**^*ref*^, the true model or the solution to the FWI problem, as d* becomes closer to **d**^*obs*^ in the data space, ℜ^*T*^. At the bottom of the figure, an example of FWI 1D shows a velocity model composed of two layers (*V*_1_ and *V*_2_) and a reflector between them and their respective seismogram trace for the observed **d**^*obs*^ and calculated data d*. It is expected that when d* ≈ **d**^*obs*^ ⇒ m* ≈ **m**^*ref*^.

The excessive computational cost related to the traditional FWI is due to the extra wavefield propagation’s required by the adjoint operator (gradient-based) and the high number of iterations to find a reliable solution. However, global optimization methods can be efficient and has better convergence rates. In addition to the ability to find global solutions, global optimization algorithms do not need an initial model closer to the true model of the problem. In fact, they can be used for finding reliable initial models that can, then, be inputted to a gradient-based FWI method in order to be improved.

Precisely, the optimization stage of the FWI problem can be treated as a nonlinear programming problem or a constrained optimization problem. In a canonical form, it is written as:

$$\text{subject}\;\text{to}\overset{\text{minimize}\;\phi \text{(m),}}{\left\{\begin{array}{l} Gm=d^{*}\\ L_{low_{j}}\leq m_{j}\leq L_{up_{j}},j=1,2,\cdots ,N \end{array}\right.}$$

In this context, *ϕ*(**m**) is called an objective function. The number of parameters of a considered model is equal to *N*, where $L_{low_{j}}$ and $L_{up_{j}}$ are restrictions associated with the lower and upper limits for each parameter *m*_*j*_ of the model. For FWI based on global optimization algorithms, there are many ways to update the model’s parameters. But they can all be written in the same way as in Eq (3). When using PSO as the optimization algorithm for the FWI problem, the search direction is the velocity of the particle in the search space, **h** = **V**, and *α*_*h*_ is equal to a negative constant. Consequently, the model is the position of the particles, **m** = **X**, resulting in a model-update formula of the form **X**^*k*+1^ = **X**^*k*^ + **V**^*k*+1^[17].

In FWI using global optimization, the dimension of the search space, that is, the number of model parameters to be optimized, drastically influences the precision and the ability of the algorithms to find an optimal solution. It may even make the solution-space search unfeasible [18, 19]. Almost all approaches applied to the FWI problem make use of some type of reparameterization of the geologic model. Reducing the number of parameters (unknowns) consequently reduces the computational cost and enables the application of the most diverse algorithms. The main strategy used in 2D problems is know as *two grids*[20], where there is an inversion grid (coarse) and a modeling grid (fine). The first grid is where the parameters are optimized, that is, these are the parameters that the inversion process changes in the search for a solution and has a low sampling rate (few parameters). The second grid is generated from the first, usually by an interpolation procedure, and is used in the forward problem, that is, to generate the synthetic data with a high sampling rate (many elements).

For 1D problems, the main strategy is known as *block* or *parametric*, where the model parameters are few, as little as possible without loss in the accuracy of the description of the model of interest, and are used to generate an application model, that is, compatible with forward modeling. In general, we can define a parameterization operator **Γ** that converts a set of parameters **m** = (*m*_1_, *m*_2_, ⋯, *m*_*N*_) in a vector or matrix that represents the scalar velocity field of the wave equation,

Eq (4). This operator can be defined, mathematically, by

$$\begin{array}{c} c=\Gamma (m), \end{array}$$ (4)

where **Γ** it is computationally simple to define. In this way, the problem can be stated as

$$\text{subject to}\overset{\text{minimize}\;\phi \left(m\right)}{\{\begin{array}{c} Gc=d^{*},c=\Gamma \left(m\right)\\ L_{low_{j}}\leq m_{j}\leq L_{up_{j}},j=1,2,\cdots ,N \end{array}}$$

### 1D Seismic problem modeling

The forward modelling corresponds to the mathematical model of propagation of an acoustic wave. The mathematical operator *G* is represented by a set of Partial Differential Equations (PDE). In this study, the phenomenon is governed by isotropic wave equation in one-dimension (1D), given from the following expression

$$\begin{array}{c} {}[\frac{1}{c^{2}(x)}\frac{\partial^{2}}{\partial t^{2}}-\frac{\partial^{2}}{\partial x^{2}}]u(x,t)=f(t)\delta (x-x_{s}), \end{array}$$ (5)

where *c* is the propagation velocity in the wavefield and *u* is the wavefield amplitude or pressure field. *δ*(*x*−*x*_*s*_) is the Dirac delta function with *x*_*s*_ the position of the source. *f*(*t*) is the signature of the seismic source (wavelet form) and *t* is the wave propagation time (in seconds) [21].

This type of PDE need two initial values (IV) and, to avoid reflections at the edges, two boundary conditions (BC), given by:

$$\begin{array}{c} IV\{\begin{array}{c} u(x,t)=0,\;t\leq 0\\ \frac{\partial }{\partial t}u(x,t)=0,\;t\leq 0 \end{array} \end{array}$$ (6)

and

$$\begin{array}{c} BC\{\begin{array}{c} \frac{1}{c(x_{0})}\frac{\partial }{\partial t}u(x_{0},t)-\frac{\partial }{\partial x}u(x_{0},t)=0\\ \frac{1}{c(x_{L})}\frac{\partial }{\partial t}u(x_{L},t)+\frac{\partial }{\partial x}u(x_{L},t)=0 \end{array} \end{array}$$ (7)

where *x*_0_ and *x*_*L*_ are the edge limits of the physical domain. The BC conditions are a mathematical artifice introduced to prevent unwanted reflections at the borders. They are one of the simplest method to numerically solve this problem in the computational domain, and belong to a larger category of approaches known as Absorbing Boundary Conditions (ABC) [21].

The signature of the seismic source *f*(*t*) in general (and also in this work) is given by the Ricker wavelet, usually obtained from the second derivative of a Gaussian function [22]. It has the following mathematical expression:

$$\begin{array}{c} f(t)=[1-2\pi^{2}\nu_{0}^{2}{\left(t-t_{0}\right)}^{2}]e^{-\pi^{2}\nu_{0}^{2}{\left(t-t_{0}\right)}^{2}}, \end{array}$$ (8)

with $t_{0}=6/(\pi \nu_{0}\sqrt{2})$ and *ν*_0_ being the peak frequency in Hertz. The term (*t*−*t*_0_) is the time shift.

### Numerical solution of the wave equation

The direct problem or forward modeling consists in determining the wavefield propagation in the geological medium, through a mathematical model that describes the physical phenomenon, in this case, the wave equation. With this in mind, it is possible to predict whether the observed data, recorded on a seismogram, matches a given model. To the acoustic wave, the model is composed of the seismic-wave propagation velocity for each point of the medium.

**Fig 2:**
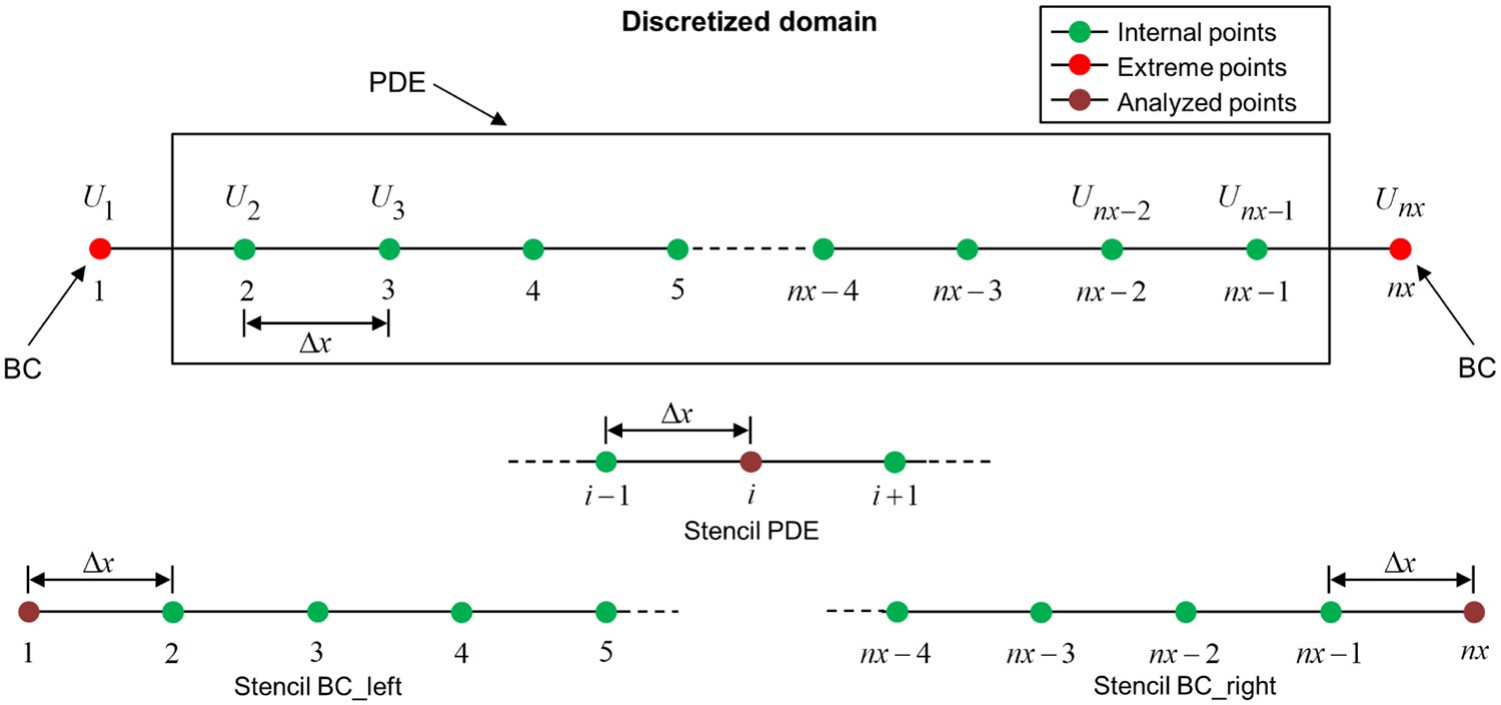
Uniform mesh of *nx* points in 1D for the FDM scheme. The image shows the stencil and points where the wave (PDE) and the boundary conditions (BC) equations are applied.

The forward modeling is described by Eq (5), which is solved numerically. There are several methods that can be used for this purpose: Finite Element Method (FEM) and Finite Difference Method (FDM) are the most used ones. In the present paper, we employ the FDM method. The essence of the FDM is the discretization of the continuous domain into a mesh of nodes, where each infinitesimal variation *dx* is now a discrete value Δ*x* with *n*_*x*_ points [21]. In that way, we leave the continuous domain for a discrete one, $u\left(x,t\right)\Rightarrow U_{i}^{t}$, where the derivatives are approximated by finite differences using only the values of $U_{i}^{t}$ in the nodal points, in order to find the solution of the PDE in those points. Fig 2 shows the spatial domain discretization scheme, the stencil used and the region where the equation that governs the phenomenon is applied, as well as, the boundary conditions. Such a discretization of the derivative is made using the Taylor series. The stencil contains the list of nodal points used in the finite difference approximation scheme, even the point under analysis.

In most FDM applications to the Eq (5) and in this work, the second spatial and temporal derivatives are approximated by second order centered difference formulas, resulting in the Eq (9). The green dots (internal points) in Fig 2 show the discrete domain application of this equation. It is a standard three-point scheme (stencil) in 1D with second-order precision [23]. There are many methods to solve ABC (Eq (7)). According to Gao *et al.* [24], one of the most precise and simple solutions is known as Reynolds boundary. In that method, the spatial and temporal first order derivatives are replaced by finite differences with first order precision. They are advanced in time and advanced and backwarded in space, to the left and right-edge limits, respectively [21]. In the present work, we use a modified Reynolds boundary method to solve the ABC equations. To improve the numerical results of the original Reynolds boundary, we apply finite difference formulas of fourth order of precision in space on the left and right edges, resulting in the Eq (10). The red dots (extreme points) in Fig 2 show the discrete domain application of this equation.

$$\begin{array}{c} \frac{1}{c_{i}^{2}}\frac{U_{i}^{t-1}-2U_{i}^{t}+U_{i}^{t+1}}{{\left(\Delta t\right)}^{2}}-\frac{U_{i-1}^{t}-2U_{i}^{t}+U_{i+1}^{t}}{{\left(\Delta x\right)}^{2}}=f_{i}^{t} \end{array}$$ (9)

$$\begin{array}{c} ABC\{\begin{array}{c} \frac{1}{c_{1}}\frac{U_{1}^{t+1}-U_{1}^{t}}{\Delta t}-\frac{-\left(25/12\right)U_{1}^{t}+4U_{2}^{t}-3U_{3}^{t}+\left(4/3\right)U_{4}^{t}-\left(1/4\right)U_{5}^{t}}{\Delta x}=0\\ \frac{1}{c_{nx}}\frac{U_{nx}^{t+1}-U_{nx}^{t}}{\Delta t}+\frac{\left(1/4\right)U_{nx-4}^{t}-\left(4/3\right)U_{nx-3}^{t}+3U_{nx-2}^{t}-4U_{nx-1}^{t}+\left(25/12\right)U_{nx}^{t}}{\Delta x}=0 \end{array} \end{array}$$ (10)

A FDM explicit scheme, which provides the wavefield in the future time **U**^*t*+1^ in function of the wavefields at the present time **U**^*t*^ and the previous time **U**^*t*−1^, for the discretized Eqs (9) and (10) for the left, internal and right numerical domains, respectively in that order, is:

$$\begin{array}{c} U_{1}^{t+1}=(1-\frac{25}{12}\lambda_{1})U_{1}^{t}+4\lambda_{1}U_{2}^{t}-3\lambda_{1}U_{3}^{t}+\frac{4}{3}\lambda_{1}U_{4}^{t}-\frac{1}{4}\lambda_{1}U_{5}^{t} \end{array}$$ (11)

$$\begin{array}{c} U_{i}^{t+1}=\lambda_{i}^{2}U_{i-1}^{t}+(2-2\lambda_{i}^{2})U_{i}^{t}+\lambda_{i}^{2}U_{i+1}^{t}-U_{i}^{t-1}+\xi_{i}^{2}f_{i}^{t},\;i=2,\cdots ,nx-1 \end{array}$$ (12)

$$\begin{array}{c} U_{nx}^{t+1}=-\frac{1}{4}\lambda_{nx}U_{nx-4}^{t}+\frac{4}{3}\lambda_{nx}U_{nx-3}^{t}-3\lambda_{nx}U_{nx-2}^{t}+4\lambda_{nx}U_{nx-1}^{t}+(1-\frac{25}{12}\lambda_{nx})U_{nx}^{t} \end{array}$$ (13)

with

$$\begin{array}{c} \left\{ \begin{array}{c} \lambda_{i}=c_{i}\frac{\Delta t}{\Delta x},\;i=1,\cdots ,nx\\ \xi_{i}=c_{i}\Delta t,\;i=2,\cdots ,nx-1 \end{array}\right. \end{array}$$ (14)

and

$$\begin{array}{c} f_{i}^{t}=\{\begin{array}{c} f(t),\;if\;x_{i}=x_{s}\\ \;0,\;if\;x_{i}\neq x_{s} \end{array}. \end{array}$$ (15)

This scheme can be seen as a linear system or a matrix equation such as

$$\begin{array}{c} U^{t+1}=AU^{t}-U^{t-1}+F^{t} \end{array}$$ (16)

or, in its expanded form, as

$$\begin{array}{c} \left[\begin{array}{c} U_{1}^{t+1}\\ U_{2}^{t+1}\\ \vdots \\ U_{i}^{t+1}\\ \vdots \\ U_{nx-1}^{t+1}\\ U_{nx}^{t+1} \end{array}]=A[\begin{array}{c} U_{1}^{t}\\ U_{2}^{t}\\ \vdots \\ U_{i}^{t}\\ \vdots \\ U_{nx-1}^{t}\\ U_{nx}^{t} \end{array}]-[\begin{array}{c} 0\\ U_{2}^{t-1}\\ \vdots \\ U_{i}^{t-1}\\ \vdots \\ U_{nx-1}^{t-1}\\ 0 \end{array}]+[\begin{array}{c} 0\\ \xi_{2}^{2}f_{2}^{t}\\ \vdots \\ \xi_{i}^{2}f_{i}^{t}\\ \vdots \\ \xi_{nx-1}^{2}f_{nx-1}^{t}\\ 0 \end{array}\right] \end{array}$$ (17)

being **U**^*t*+1^, **U**^*t*^, **U**^*t*−1^ and **F**^*t*^ vectors of size (*nx* × 1) with **F**^*t*^ the source vector and **U**^*t*+1^, **U**^*t*^ and **U**^*t*−1^ the wavefield vectors (internal points). **A** is a three-diagonal matrix, except the first and last line, of the length (*nx* × *nx*), expressed by:

$$\begin{array}{c} A=[\begin{array}{ccccccc} 1-\frac{25}{12}\lambda_{1} & 4\lambda_{1} & -3\lambda_{1} & \frac{4}{3}\lambda_{1} & -\frac{1}{4}\lambda_{1} & \ldots & 0\\ \lambda_{2}^{2} & 2-2\lambda_{2}^{2} & \lambda_{2}^{2} & 0 & 0 & \ldots & 0\\ 0 & \lambda_{3}^{2} & 2-2\lambda_{3}^{2} & \lambda_{3}^{2} & 0 & \ldots & 0\\ \vdots & \vdots & \vdots & \vdots & \vdots & \cdots & \vdots \\ 0 & 0 & 0 & \lambda_{nx-2}^{2} & 2-2\lambda_{nx-2}^{2} & \lambda_{nx-2}^{2} & 0\\ 0 & 0 & 0 & 0 & \lambda_{nx-1}^{2} & 2-2\lambda_{nx-1}^{2} & \lambda_{nx-1}^{2}\\ 0 & 0 & -\frac{1}{4}\lambda_{nx} & \frac{4}{3}\lambda_{nx} & -3\lambda_{nx} & 4\lambda_{nx} & 1-\frac{25}{12}\lambda_{nx} \end{array}] \end{array}$$

These equations represent an evolution scheme of the temporal variable for extreme and internal nodes where, for each time step, the solution $U_{i}^{t}\Rightarrow u\left(x,t\right)$ is found in an explicit way. Δ*x* and Δ*t* are the steps in space and in time of the FDM discretization. Furthermore, the Courant-Friedrichs-Lewy (CFL) condition is the stability criterion that must be satisfied so that the system can converge to an accurate solution. This condition is described as follows [23]:

$$\begin{array}{c} \Delta t\leq \alpha \frac{\Delta x}{c_{max}}, \end{array}$$ (18)

where *c*_*max*_ = ‖**c**‖_∞_ is the maximum velocity of wave propagation in the geological environment (velocity model). For a stable solution, due to the approximations made, *α* = 1/6.

### Derivative-free optimization

Meta-heuristics are finite, step-by-step, well-defined DFO procedures that are capable of solving an optimization problem. Unlike heuristics, they are equipped with more sophisticated mechanisms and make use of different strategies to explore, in a more embracing and efficient way, the search space for an optimal solution. Most meta-heuristics are inspired by nature. Some are bio-inspired algorithms, like Genetic Algorithms (GA) [25] and Particles Swarm Optimization (PSO) [26, 27], while others are based on physical phenomena, such as Simulated Annealing (SA) [28]. Non-bio-inspired DFO techniques can be purely stochastic, as the Monte Carlo (MC) method [29]. Considering the usage of fundamental mathematical operations, such as geometric transformations in a Simplex (Convex polyhedron), there are several approaches as the Nelder-Mead Simplex algorithm [30, 31]. There are also methods that combined the two previous characteristics, such as the Controlled Random Search (CRS) method [32]. Furthermore, some meta-heuristics are population-based random optimization techniques, such as PSO and GA.

Most of the knowledge about these methods is of an empirical nature, which makes difficult a rigorous analysis of the convergence towards the global optimum. The methods are also relatively slow and scale poorly with the dimensionality of the problem. However, they are robust and easy to implement and can deal with complex search spaces, as it is the case of the FWI problems. According to Koduru, Das and Welch [7], the main advantages of bio-inspired stochastic optimization techniques (or meta-heuristics) are: they are immensely popular, do not get trapped easily in local minima, sample a wide region of the search space, can be customized to suit a specific problem and can be easily hybridized with other algorithms in order to improve their overall performance.

As previously mentioned, PSO and Nelder-Mead Simplex and the variants are the most important algorithms in the present context, as they are integrated for composing our hybrid algorithm. Therefore, they are described in more detail next. We also present the K-means algorithm developed by Hartigan and Wong [33], employed for better exploring the search space.

#### Particle swarm optimization

PSO is a population-based random optimization technique developed by Kennedy and Eberhart [26] and further improved by Shi and Eberhart [34]. Its inspiration came from the collective behavior of a system formed by flying birds or school of fish in nature, Kennedy [35]. These can be considered a decentralized self-organizing system. It can be considered an evolutionary algorithm, which population is initialized by feasible random solutions called particles, of dimension *N*. The technique maintains a swarm of *M* particles, with the position of each particle *i* in the feasible search space represented by the vector $X_{i}^{k}=\left(x_{i1}^{k},\cdots ,x_{iN}^{k}\right)$, or simply **X**_*i*_, with *k* the iteration number. In every step *k*, the position of each particle *i* is updated by adding to it an instantaneous velocity vector $V_{i}^{k}=\left(v_{i1}^{k},\cdots ,v_{iN}^{k}\right)$, or simply **V**_*i*_. For short, we can write the update equation for all particles of the swarm in the *k*-th iteration as follows:

$$\begin{array}{c} X^{k+1}=X^{k}+V^{k+1}. \end{array}$$ (19)

In order for the particle to move towards a better location, a less costly solution, the velocity is updated in each iteration. The velocity change occurs using the best previous position registered by the particle itself, as well as the current location of the other particles. It is given by Koduru, Das and Welch [7]:

$$\begin{array}{c} V^{k+1}=wV^{k}+C_{1}r_{1}\left(X_{kb}^{k}-X^{k}\right)+C_{2}r_{2}\left(X_{gb}^{k}-X^{k}\right), \end{array}$$ (20)

where **X** ∈ *Ω* ℜ^*N*^, and *Ω* is the feasible search space, defined by a subset

$$\begin{array}{c} \Omega =\left[L_{low\_1},L_{up\_1}\right]\times \left[L_{low\_2},L_{up\_2}\right]\times \cdots \times \left[L_{low\_N},L_{up\_N}\right], \end{array}$$ (21)

being *L*_*low*_*j*_ and *L*_*up*_*j*_ are, respectively, the lower and upper bounds of the search space what corresponds to a hyper-box *Ω*, along dimension *j* for *j* = 1, ⋯, *N*.

The description of the other terms of Eq (20) are: *C*_1_ and *C*_2_ are the acceleration coefficients, also called the *cognitive* and the *social* parameters, respectively, that reflect the weight of stochastic acceleration terms pulling each particle. The *r*_1_ and *r*_2_ parameters denote two uniformly distributed random numbers in the [0, 1] interval. *w* is called the inertial coefficient, that helps in maintaining convergence stability. A large value in *w* generally makes global exploration easier while a small value facilitates local search (exploitation) [11]. $X_{kb}^{k}$ is called the *individual best* and is the best recorded position of any particle, until the current iteration. $X_{gb}^{k}$ is called the *global best* and is the position of the best particle in the current iteration. In this way, the movement of the particles occurs in terms of inertia, memory and cooperation.

Some considerations about how the terms that appear in the Eq (20) influence the movement of the particles are presented now. The inertial term acts as a flight memory, preventing particles from drastically changing direction. It is a term for the momentum, necessary for the particles to travel the search space. The cognitive term quantifies the individual performance of each particle, causing each one to be attracted back to its best position. It is a term of individual memory (nostalgia). The social term quantifies the performance of each particle collectively in a given neighborhood, causing all particles to be attracted to the best position determined by that neighborhood. It is a term of collective memory.

The PSO uses an objective function $f(X_{i}^{k})$, in order to evaluate each solution $X_{i}^{k}$, in the *k*-th iteration. In this context, the fitness value of each particle *i* of the swarm is $fit\left(X_{i}^{k}\right)=f\left(X_{i}^{k}\right)$, where *fit*(.) is the generalized form to any objective function. In FWI case, *fit*(.) = *ϕ*(**m**).

As implied before, the PSO algorithm needs to maintain three vectors that characterize a particle *i*: its position $X_{i}^{k}=\left(x_{i1}^{k},\cdots x_{iN}^{k}\right)$, its velocity $V_{i}^{k}=\left(v_{i1}^{k},\cdots v_{iN}^{k}\right)$ and its best position experienced until the present moment, held in $X_{kb}^{k}$. Beyond the vector that features the swarm $X_{gb}^{k}$, the best position experienced by the swarm at the present moment and *w*, *C*_1_, and *C*_2_, inertia factor and acceleration coefficients, respectively. For a function *f*(**X**): ℜ^*N*^ → ℜ considering a minimization problem and a global neighborhood, the steps of the PSO algorithm for each iteration *k* can be listed as follows [34]:

0 Initialization: randomly initialize the positions **X**^*k*^ and velocities **V**^*k*^, for the swarm, and define *k* = 1;
1 Best positions: for each particle *i* of the swarm;
   Calculates the fitness value: $fit\left(X_{i}^{k}\right)=f\left(X_{i}^{k}\right)$;
   If $fit\left(X_{i}^{k}\right)<f\left(X_{kb}^{k}\right)$, do
    $X_{kb}^{k}=X_{i}^{k}$
   End If
   If $fit\left(X_{kb}^{k}\right)<f\left(X_{gb}^{k}\right)$, do
    $X_{gb}^{k}=X_{kb}^{k}$
   End If
2 Swarm update: for the entire swarm,
   update the velocity vector,
   
$V^{k+1}=wV^{k}+C_{1}r_{1}\left(X_{kb}^{k}–X^{k}\right)+C_{2}r_{2}\left(X_{gb}^{k}-X^{k}\right)$
   and update the position vector,
  **X**^*k*+1^ = **X**^*k*^ + **V**^*k*+1^
3 Make *k* = *k* + 1 and, if a stopping criterion is not satisfied, return to step 1 for starting a new iteration.

**Fig 3:**
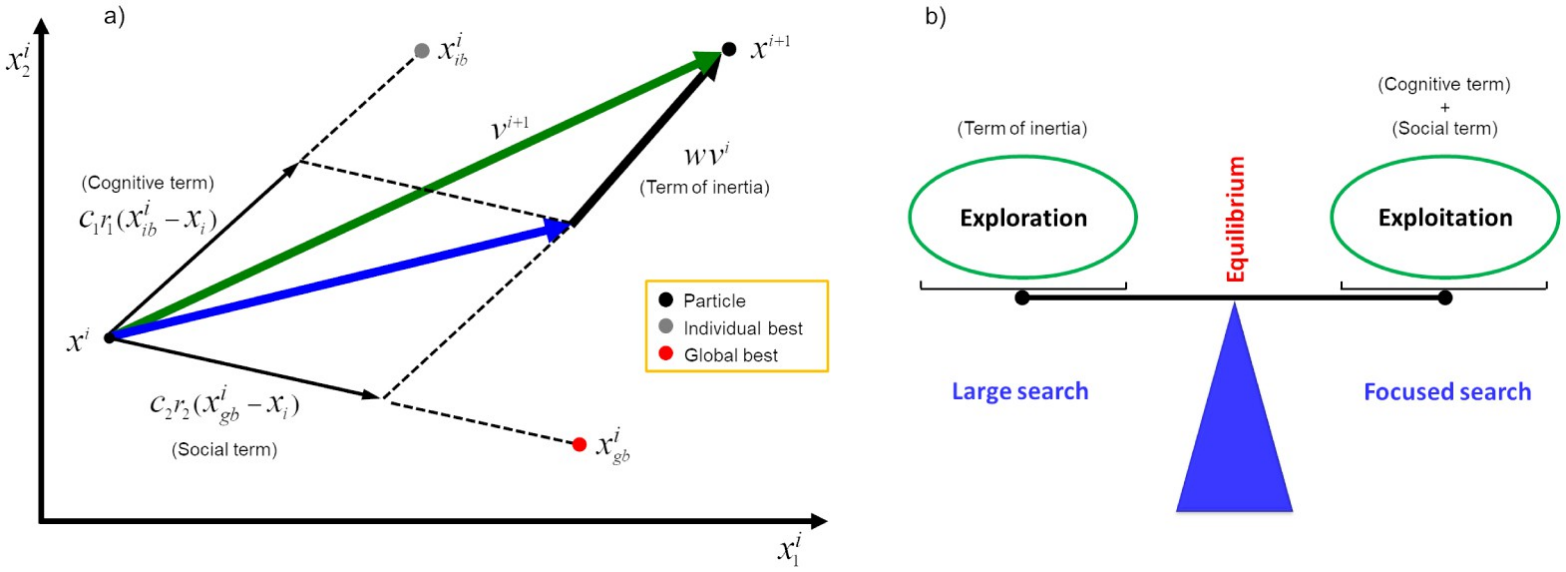
Exploration *versus* exploitation. (a) The three vectors associated with updating the velocity of particle in the original PSO—inertia, cognitive and social learning terms and (b) Balancing between exploration and exploitation mechanisms.

The usual stopping criterion for PSO is to reach a maximum number of iterations (*k* > *It*_*max*_). Fig 3a shows a summary of the vector operations on a particle *i* of the swarm in the *k*-th iteration. In this case, *N* = 2 and $X_{i}^{k}=\left(x_{i1}^{k},\;x_{i2}^{k}\right)$. We then have: the inertia term, that forces the particle to move in the same direction; the cognitive term, that forces the particle to follow its best position; and the term of social learning, that forces the particle to follow the direction of the best swarm position.

One of the great challenges of metaheuristics based on a swarm of particles is to find an ideal balance between the mechanisms of exploration (diversification) and exploitation (intensification). Exploration provides a larger search for new solutions, identifying regions with the potential for better solutions. On the other hand, exploitation provides a more focused search based on solutions already found, thus looking for the best solution in a region. Fig 3b illustrates the balance between these search mechanisms for better solutions.

According to Rana, Jasola and Kumar [6], the main advantages of the PSO algorithm are the few parameters to be optimized and to require little execution memory. However, there are disadvantages that limits its application such as: a very slow convergence rate when close to the global solution; and its fast and premature convergence at non-optimal points. The problem of slow convergence is related to particles converging to the global best, when all particles move to a single point between the best global position and the best individual position. The rapid flow of information between particles is another reason for this problem, resulting in the creation of similar particles (loss of diversity), which increases the risk of trapping them in a local minimum. The problem of premature convergence in multimodal functions is related to its weak local search capability [11].

We can divide the parameters that characterize the PSO method into two classes: initial and control parameters. The initial parameters are the swarm size *M*, the positions **X**^*k*^ of the particles, their velocities **V**^*k*^ and the maximum number of iterations allowed *It*_*max*_. The control parameters are: the inertial weight *w*, the acceleration coefficients *C*_1_ and *C*_2_, the maximum limited velocity *V*_*max*_, the swarm topology (neighborhood) and the stopping criterion. In addition to reaching a maximal iteration number, *It*_*max*_, the stopping criterion can be based on the precision of the update of the best solution. In general, all of these parameters are user-defined and need to be carefully chosen. There are significant literature helping how to understanding and to set them. Next, we present some considerations and analysis of admissible values for such parameters.

The inertial *w* controls the impact of the current velocity in updating the direction of the particle. As previously mentioned, a large inertia weight contemplates exploration (a global search), making the swarm divergent, while a small inertia contemplates exploitation (a local search), decelerating the particles. There are several possibilities for the value of *w*: a constant, a value multiplied by a damping ratio in every iteration, and a variable linearly decreased with iterations, among other options [36].

The acceleration coefficients *C*_1_ and *C*_2_ determine the tendency of search. If *C*_1_ = *C*_2_ = 0, then the particles move in the same direction to the limit of the search space; If *C*_1_ > 0 and *C*_2_ = 0, then each particle performs its local search towards $X_{kb}^{k}$; If *C*_1_ = 0 and *C*_2_ > 0, all particles are attracted to a single point $X_{gb}^{k}$; If *C*_1_ = *C*_2_, each particle is attracted to the barycenter between $X_{kb}^{k}$ and $X_{gb}^{k}$; If *C*_1_ ≫ *C*_2_, the particles are attracted towards their $X_{kb}^{k}$ positions, resulting in dispersion; And if *C*_1_ ≪ *C*_2_, the particles are attracted towards $X_{gb}^{k}$, resulting in premature convergence to local optima. With low values of *C*_1_ and *C*_2_, the result is a smooth movement of the particles. With high values of these parameters, the movements of the particles are abrupt.

#### Modified PSO

In the standard PSO, the parameters of inertia *w* and social behaviors, *C*_1_ and *C*_2_, are considered constant. Generally, *w* ∈ [0, 1] and *C*_1_ + *C*_2_ > 4. However, several studies have analyzed the ranges of values of these variables for a better performance of the algorithm. In his studies, Clerc [37] recommended *w* = 0.729 and *C*_1_ = *C*_2_ = 1.494. This set of parameters was tested by Eberhart and Shi [38], giving good results. Shi and Eberhart [34] found that *w* ∈ [0.8, 1.2] resulted in fast convergence. Zahara and Hu [39] suggested *C*_1_ = *C*_2_ = 2 and $w=0.5+\frac{rand}{2}$. Other experiments [40, 41] show that a linear decrease over time from 0.9 to 0.4 in *w* would provide good results. Those studies showed that, with the use of an adaptive inertial weight strategy, the PSO has the ability to quickly converge, its performance becomes insensitive to the population size, and the method scales well. According to Dai, Liu and Li [42], when *w* ∈ [0.5, 0.75], the success rate for optimal is very high, more than 80%. The same authors also affirm that, if *C*_2_ ∈ [0.5, 2.5], approximately, the algorithm shows a better performance. A version of the PSO with time-varying acceleration coefficients was considered by Ratnaweera, Halgamuge and Watson [43]. The best ranges suggested by them for *C*_1_ and *C*_2_ in most benchmark functions were [2.5, 0.5] and [0.5, 2.5], respectively. These values were gradually varied over the iterations. The procedure tended to increase the diversity at the beginning of the search and at the end, when the algorithm was usually converging to the optimal solution and therefore giving more importance to intensification (fine tuning of the solutions). In addition, Suganthan [44] used the strategy of adaptive parameters for both inertial weights and acceleration coefficients, citing advantages and disadvantages.

As adaptive particle swarm optimization (APSO) provides, in general, better search efficiency than a standard PSO, the coefficient of inertia, *w*, and the acceleration coefficients, *C*_1_ and *C*_2_, among other parameters, were considered dynamic in the present work, with linear variation over iterations. The parameters and coefficient above mentioned are updated using the Eq (22), where *S* identifies each of them.

$$\begin{array}{c} S=S_{initial}-(\frac{It}{It_{max}})(S_{initial}-S_{final}) \end{array}$$ (22)

The idea is that, initially, the individual experiences of each particle receive greater importance and then gradually, the collective experience is favoured. This allows the swarm to better explore the search space for solutions, giving the algorithm greater diversity, thus avoiding possible premature convergence. In addition, limits were adopted for the particles’ velocities. Velocity clamping is calculated as a percentage *η*, of the range of the search space along the coordinate axes, by the following equation [45]:

$$\begin{array}{c} V_{j,max}=\eta \times range_{j}\left(\Omega \right), \end{array}$$ (23)

where

$$\begin{array}{c} range_{j}\left(\Omega \right)=L_{up,j}-L_{low,j},\;j=1,\cdots ,N \end{array}$$ (24)

Then we make, *V*^*k*+1^ ∈ [−*V*_*j*,*max*_, *V*_*j*,*max*_]. The velocity clamping percentage is such that 0.1 < *η* < 0.5. In our studies we adopted *η* = 0.15.

As for the influence of population size *M*, Dai, Liu and Li [42] concludes that, when the number of particles is very small, the iteration is fast but the convergence rate is low and the global search performance is poor. When the number of particles increased to 10, there was a gain of 80% more. When the number of particles increased by more than 20, there was no improvement; even worse, it took more CPU time. The studies done by Shi and Eberhart [41] indicated that the PSO is not sensitive to the size of the population. However, in Carlisle and Dozier [46], it was found that this is generally true in terms of performance, but not in terms of computational cost. Furthermore, in studies with benchmark functions for optimization, it was concluded that a population of 30 particles appears to be a good choice.

The swarm topology defines the neighborhood of each particle, where communication between them occurs. This involves the number of neighboring particles that influence the movement of a given particle of the swarm. Different topologies have been used to control the flow of information between particles. Kennedy [47] and Medina *et al.* [48] designed four different topologies, including *circle, wheel, star and random edges*. The standard PSO uses the star topology as a communication structure. In this approach, all particles communicate globally, sharing the best position of a single particle. This can cause a premature convergence of the swarm. Therefore, it requires researching the neighborhood of individuals to improve the PSO’s performance over the course of iterations. For example, the neighborhood can be gradually expanded from an individual particle to include all particles of the swarm. In this research is not considered analysing neighborhood.

In Eberhart and Kennedy [49], the authors concluded that a local neighborhood is good to avoid local minimums, while a global neighborhood converges faster. In an experiment with 30 particles, Carlisle and Dozier [46] varied the neighborhood size from 2 to the global in steps of 2 and came to the following conclusion: a global neighborhood appears to be a better general choice, as it seems to achieve the same results with less work. Suganthan [44], in an experiment that gradually increased the neighborhood size to the global one, obtained an inconclusive analysis of the results. In Wang, Sun and Li [50], a hybrid PSO algorithm was proposed. It employed a diversity enhancing mechanism and reverse neighborhood search strategies to achieve a trade-off between exploration and exploitation abilities.

In our current work, we adopt the global topology strategy. Furthermore, following the ideas contained in the Attractive and Repulsive Particle Swarm Optimization (ARPSO) [51] and in the Opposition-based Particle Swarm Optimization (OPSO) [52], the present work defines a swarm composed of attractive and repulsive particles. The number of repulsive particles is dynamic, decreasing linearly with the iterations. It starts high and ends low. In addition, these repulsive particles are rebel, that is, sometimes they are repulsive, sometimes they are attractive, depending on whether a certain threshold *τ* is reached. The threshold also decays with iterations. The number of repulsive (possibly rebels) particles is a percentage *rr*_*p*_ of the swarm population. Again, the idea is to add greater diversity to the PSO algorithm. In the beginning, the swarm have a more adverse behavior and it tends towards a more collaborative behavior at the end. The update of the particle velocities is given by the following equation:

$$\begin{array}{cc} V^{k+1}=wV^{k}+dir[C_{1}r_{1}(X_{kb}^{k} & x2013;X^{k})+C_{2}r_{2}\left(X_{gb}^{k}-X^{k}\right)], \end{array}$$ (25)

where

$$\begin{array}{c} dir=\;\{\begin{array}{c} -1,\;if\;rand\leq \tau \\ \;\;1,\;if\;rand>\tau \end{array}\;, \end{array}$$ (26)

and *dir* (- 1 or 1) is used to define whether the rebel particles will expand or contract. The term *rand* is a random number in [0, 1] obtained for each iteration and *τ* ∈ [0, 1] is the *rebellion threshold*, previously defined.

Table 1 also shows the percentage of the population that will be rebel particles, *rr*_*p*_, and the values for *τ* that were used in the current research. The variations for these parameters, along the iterations, follow the same rule given by Eq (22).

**Table 1:** The initial and final values for each PSO parameter in all application. Value range for *w*, *C*_1_, *C*_2_, *rr*_*p*_ and *τ* used in this research.

Parameters	Values	
	*Initial*	*Final*
*w*	0.9	0.2
*C*_1_	2.5	0.5
*C*_2_	0.5	2.5
*rr*_*p*_(%)	80	20
*τ*	0.35	0.15

#### Adaptive Nelder-Mead Simplex line search

The Nelder-Mead Simplex (Nelder-Mead, downhill Simplex or amoeba) algorithm, published in 1965, belongs to a more general class of direct line search algorithms. It is among the steepest descent methods that do not use derivatives, that is, a non-linear optimization technique based on DFO. It can be employed to find the optimal point of an unconstrained objective function in a multidimensional space. Despite presenting convergence problems for high dimension problems, the Nelder-Mead Simplex is widely used in several low dimension optimization problems due to its simplicity. Basically, the Nelder-Mead Simplex consists of a Simplex method that minimizes a function of *N* variables, by evaluating this function on *N* + 1 vertices. In the Nelder-Mead Simplex approach, the Simplex is constructed by the replacement of the vertex with the highest value by another lesser value of the objective function. The Nelder-Mead Simplex process is adaptive, causing Simplex to be continually revised to better suit the nature of the response surface (search space topology). That is, the Simplex itself adapts to the local landscape and contracts to a minimum local solution. From a computational point of view, this method proved to be a compact and effective solution to many nonlinear problems [30, 53].

Four scalar coefficients are required for the Nelder-Mead Simplex algorithm: a reflection coefficient *ρ* > 0; an expansion coefficient *χ* > 1 and with *χ* > *ρ*; a contraction coefficient 0 < *γ* < 1; and a reduction coefficient 0 < *σ* < 1. In the standard version of the Nelder-Mead Simplex method, these coefficients are fixed: *ρ* = 1.0, *χ* = 2.0, *γ* = 0.5 and *σ* = 0.5. The adapted version, Adaptive Nelder-Mead Simplex (ANMS), is an implementation of the ANMS method in which these coefficients depend of the dimension of an *N*-dimensional optimization problem, as follows [54]:

$$\begin{array}{c} \rho =1.0,\;\chi =1+\frac{2}{N},\;\gamma =0.75-\frac{1}{2N},\;\sigma =1-\frac{1}{N}. \end{array}$$ (27)

This tries to overcome the convergence difficulties found by the Nelder-Mead Simplex for problems with high dimensions, *N* > 2. Note that when *N* = 2, the ANMS is identical to the standard Nelder-Mead Simplex. In this case, the vertices of the Simplex form a triangle.

The Nelder-Mead Simplex method considers four distinct operations that move the Simplex towards the centroid of the *N* best vertices and one operation that causes its shrinkage towards its best vertex. Let **X**_1_, **X**_2_, ⋯, **X**_*N* + 1_ be the vertices that define a Simplex in ℜ^*N*^ and *ρ*, *χ*, *γ* and *σ*, the coefficients of reflection, expansion, contraction and reduction, respectively. For an objective function *f*(**X**): ℜ^*N*^ → ℜ, the steps of the Nelder-Mead Simplex algorithm for each iteration *k* are as follows [31]:

0 Receive the *N* test points (randomized or from another stage, as a previous solution) and define *k* = 1;
1 Sort the vertices of the Simplex by increasing cost: *f*(**X**_1_) ≤ *f*(**X**_2_)⋯ ≤ *f*(**X**_*N* + 1_).
   Calculates the centroid **C** of the *N* best points;
  $$\begin{array}{c} C=\frac{1}{N}\sum_{j=1}^{N}X_{j} \end{array}$$ (28)
2 Reflection: calculate the reflection point **X**_*r*_ from;
  $$\begin{array}{c} X_{r}=\left(1+\rho \right)C-\rho X_{N+1} \end{array}$$ (29)
   If *f*(**X**_1_) ≤ *f*(**X**_*r*_) < *f*(**X**_*N*_), accept the point **X**_*r*_ and go to step 6.
3 Expansion: If *f*(**X**_*r*_) < *f*(**X**_1_), calculate the expansion point **X**_*e*_;
  $$\begin{array}{c} X_{e}=\left(1+\rho \chi \right)C-\rho \chi X_{N+1} \end{array}$$ (30)
   If *f*(**X**_*e*_) < *f*(**X**_*r*_), accept **X**_*e*_ and go to step 6. Otherwise (*f*(**X**_*e*_) ≥ *f*(**X**_*r*_)), accept **X**_*r*_ and go to step 6.
4 Contraction: If *f*(**X**_*r*_) ≥ *f*(**X**_*N*_), make a contraction;
   a) Outside: If *f*(**X**_*N*_) ≤ *f*(**X**_*r*_)<*f*(**X**_*N* + 1_), perform an outside contraction: calculate point **X**_*c*_;
  $$\begin{array}{c} X_{c}=\left(1+\rho \gamma \right)C-\rho \gamma X_{N+1} \end{array}$$ (31)
   If *f*(**X**_*c*_) ≤ *f*(**X**_*r*_), accept **X**_*c*_ and go to step 6. Otherwise, go to step 5.
   b) Inside: If *f*(**X**_*r*_) ≥ *f*(**X**_*N* + 1_), perform an inside contraction: calculate point **X**_*cc*_;
  $$\begin{array}{c} X_{cc}=\left(1-\gamma \right)C+\gamma X_{N+1} \end{array}$$ (32)
   If *f*(**X**_*cc*_) < *f*(**X**_*N* + 1_), accept **X**_*cc*_ and go to step 6. Otherwise, go to step 5.
5 Perform a shrink step. Calculate the vectors:
  $$\begin{array}{c} \nu_{j}=X_{1}+\sigma \left(X_{j}-X_{1}\right),\;j=2,\cdots ,N+1. \end{array}$$ (33)
   Thus, the the vertices of the Simplex (still out of order) for the next iteration are: **X**_1_, *ν*_2_, ⋯, *ν*_*N* + 1_.
6 Make *k* = *k* + 1 and return to step 1 to start a new iteration, or stop.

The stop criteria for Nelder-Mead Simplex usually evaluate the difference between the objective function value of the best and the worst solutions or the reduction in size of the Simplex, and compare them to a certain acceptable threshold. Another commonly adopted stop criterion is to reach a maximum number of permitted iterations of the algorithm.

In the current work, we compute the standard deviation of the objective function values calculated at the vertices of the Simplex. When it is less than a tolerance *α*_*s*_, the ANMS stops. The *α*_*s*_ parameter can be considered a shrinkage factor of the Simplex.

**Fig 4:**
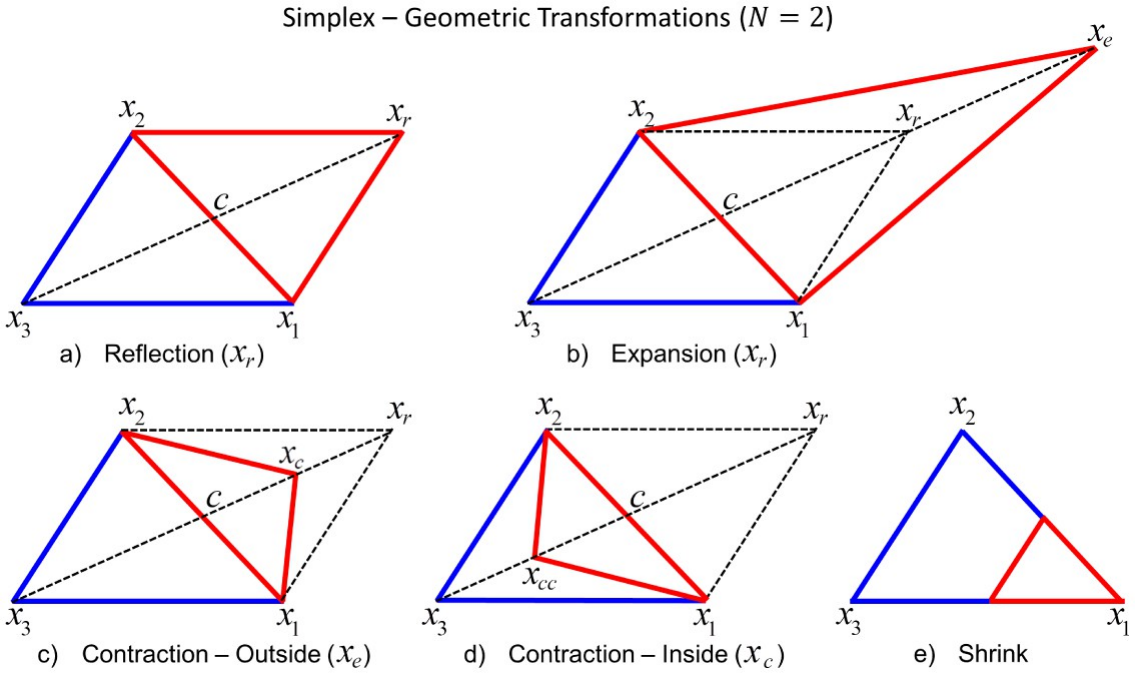
Movements defined in the original Nelder-Mead Simplex. (a) Reflection of **X**_3_, given by vertex *X*_*r*_, (b) Expansion, represented by vertex *X*_*e*_, (c) Outside contraction, given by vertex *X*_*c*_, (d) Inside contraction, defined by vertex *X*_*cc*_ and (e) shrinkage, represented by a smaller triangle of vertices. Figure adapted from: http://www.scholarpedia.org/article/Nelder-Mead_algorithm.

Fig 4 shows a summary of the five geometric operations performed on the Simplex. In the figure, *N* = 2, the vertices are **X**_1_, **X**_2_ and **X**_3_, *C* is the centroid, **X**_3_ is the worst vertex and **X**_1_ is the best vertex.

The Nelder-Mead Simplex is easy to implement and fast to run. Nevertheless, its standard version has some disadvantages: As previously mentioned, it becomes inefficient as the dimension of the problem grows, i.e., it scales badly with the dimension of the optimization problem; In some cases, the algorithm may converge to points that are neither the maximum nor the minimum of the objective function (in other words, the Simplex degenerates—flatten or become needle-shaped); Difficulties in choosing a good stopping criterion for the Nelder-Mead Simplex and its slow convergence for some types of problems were reported in many applications [55]; and, in addition to the already known drawbacks, it is a local optimization method.

Fortunately, there are many studies in the literature on variants of the Nelder-Mead Simplex search method: Lagarias *et al.* [31] and McKinnon [56] did convergence analysis; Gao and Han [54] and Mehta [57] proposed adaptive parameters; and Fajfar *et al.* [58] using the perturbed centroid to improve performance for problems with higher dimensions in Nelder-Mead Simplex method. Those studies contributed to make Nelder-Mead Simplex a more robust, reliable and competitive technique for solving nonlinear optimization problems.

There are several approaches to determine the vertices of the Initial Simplex: a random search of the initial Simplex in a hyper-box delimited by the restrictions of the problem (border or contour constraint); the usage of a relatively large starting Simplex; and building an initial Simplex in an N-dimensional space from a point **P**_1_ as a corner and N points, each marked at a distance of *β* × *range*_*j*_(*Ω*) from **P**_1_ along the coordinate axes, to obtain the remaining vertices of the Simplex [5]. If the intention is to use the ANMS as a local search method in combination with a global optimization algorithm (meta-heuristic), one can use a relatively smaller initial Simplex, as being an almost regular Simplex. Such procedure was adopted in the present work.

The step size coefficient or, simply, step size *β* is a problem dependent factor [5]. It depends on the limitation values for each dimension. This procedure forms a rectangular Simplex at **P**_1_ (almost regular Simplex), preserving the quality of the Simplex: the more equilateral the better.

$$\begin{array}{c} P_{j}=P_{1}+\left(0,\cdots ,\beta \times range_{j}\left(\Omega \right),\cdots ,0\right),\;j=1,\cdots ,N \end{array}$$ (34)

In the Eq (34), *N* is the dimension of the problem’s search space.

### K-means clustering algorithm

The clustering technique consists of the automatic grouping of similar instances. That is, it classifies data in homogeneous groups. Numerically, the similarity between the data can be measured by distance metrics. K-means is the most used algorithm for clustering and consists of a basic method with low computational effort. The purpose of the K-means clustering algorithm is to partition a set of *M* observations into the *N*-dimensional space in *n*_*c*_ clusters with their respective cluster center (centroids). Thus, minimizing the sum of the moments of inertia of each cluster. Mathematically, the K-means algorithm aims to minimize the sum of the quadratic error in the clusters, according to the following objective function which corresponds to the total moment of inertia *I*.

$$\begin{array}{c} I=\sum_{i=1}^{n_{c}}\sum_{X\in Cl_{i}}{∥X-C_{i}∥}_{2}^{2}, \end{array}$$ (35)

where {**X**_1_, **X**_2_, ⋯, **X**_*M*_} is the set of *M* observations, *n*_*c*_ number of clusters, designated by the set $\left\{Cl_{1},Cl_{2},\cdots ,Cl_{n_{c}}\right\}$ and ‖.‖_2_ is the Euclidean distance measure between a data point **X** ∈ *Cl*_*i*_ and the centroid **C**_*i*_ of the *i*-th cluster.

Initially the centroids are taken randomly in the *N*-dimensional space. To form the clusters take each observation from the data set and associate it with the nearest centroid. That is, the distance between it and the centroid of that cluster is minimal, when compared to the centroid of the remaining clusters. At this point, we need to update the new *n*_*c*_ centroids based on the observations in each cluster. A new association must be made between the same data set and the new nearest centroids. Then, we return to the previous step to update the new *n*_*c*_ centroids. A loop is formed that ends when updates are no longer possible. In summary, K-means receives the number of clusters *n*_*c*_, the initial positions for the *n*_*c*_ centroid and the data set to be analyzed. Then iteratively updates these positions until can no longer.

The K-means clustering algorithm could be written of the following steps [10]:

1 Place *n*_*c*_ points into the space. These points represent the initial centroids.
2 Assign each point of the swarm to the nearest centroid to build the *n*_*c*_ clusters.
3 When all points have been assigned, recalculate the positions of the *n*_*c*_ centroids.
4 Repeat steps 2 and 3 until the centroids no longer move. This produces a separation of the points into groups from which the metric to be minimized can be calculated.

**Fig 5:**
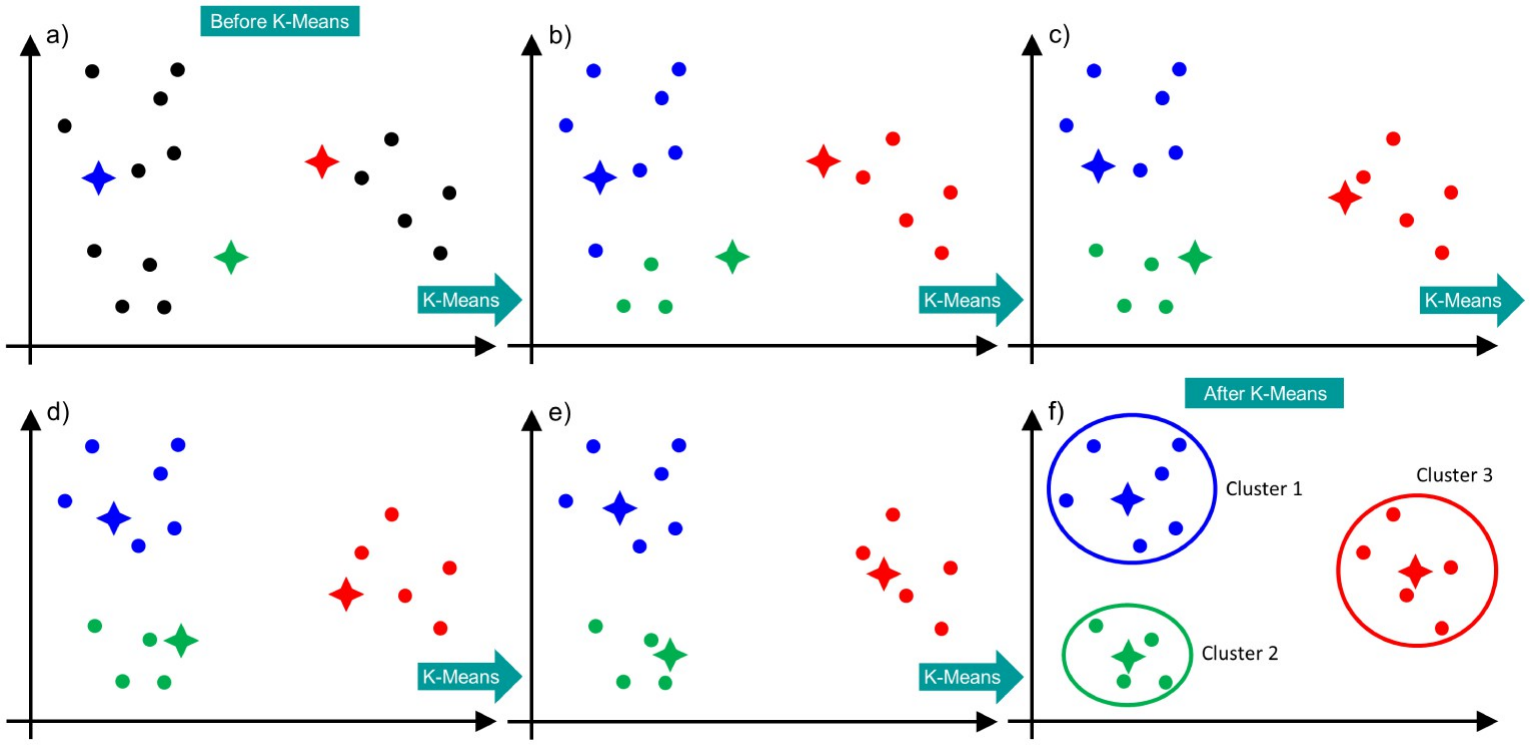
Schematic visualization of the work of the K-means algorithm. The data is in black. The stars mark the centroids. The clusters are represented by the colors blue, green and red.

The Fig 5 schematically shows how the K-means clustering algorithm works. In this example we have: *M* = 15, *N* = 2 and *n*_*c*_ = 3. A given disordered group of points is ordered into three clusters with their respective centroids by the K-means algorithm. Fig 5a presents the observed data (black dots) and the random choice of the initial centroids (colorful crosses). Fig 5b shows the assignment of each point to the nearest centroid. Fig 5c–5e present the updates of the assignment of each point to the nearest centroid. Finally, Fig 5f shows again the assignment of each point to the nearest centroid and from that moment on the centroids no longer move, ending the K-means algorithm.

In the practical applications of the K-means algorithm, two questions are important. One is the ideal number of cluster *n*_*c*_ and the second is initial centroids. Generally, the number of *n*_*c*_ clusters is human defined and the initial centroids taken at random in *N*-dimensional space. Then, in the process of optimizing the moment of total inertia *I* the centroids are reallocated in such a way that all points can be grouped correctly.

### Improved parameter space sampling

The construction of initial solutions is a very important part of heuristic methods. Improper sampling of the parameter space can severely impair the convergence and accuracy of most algorithms in this class. However, there are several techniques used to mitigate this problem. Stratified sampling is a widely used strategy to improve the convergence of algorithms based on the Monte Carlo method. The efficiency of a stratifying technique mainly depends on the coherence of the stratus. Steigleder & McCool [59] applied the Hilbert curve as a stratifying technique, where it is possible to draw an arbitrary number of stratified samples from N-dimensional spaces using only one-dimensional stratifying.

The Hilbert curve maps points with multidimensional coordinates to a straight line and vice versa with some proximity preservation, and can be used to reduce multidimensional to unidimensional problems [60]. This facilitates the sampling of values in the parameter space, ensuring a distribution that maps the entire search space, promoting greater sample diversity. This approach was used for all problems, algorithms and tests performed in this work.

#### The Hilbert curve

Hilbert curve, also known as a Hilbert space-filling curve, is a continuous curve that sequentially fills an entire *N*-dimensional space [61]. The Hilbert curve preserves vertices (points) proximity fairly well. This means that two data points that are close to each other in one-dimensional space are also close to each other after folding in the *N*-dimensional space. The converse is not always true [62]. The Hilbert curve has many applications, recently, Araújo, Gross and Xavier-de-Souza [63] applied to reorder mesh elements in memory for use with the Spectral Element Method, aiming to attain fewer cache misses, better locality of data reference and faster execution. Furthermore, it is applied as a heuristics base for the traveling salesman problem, which consists of discovering the route that minimizes the total journey.

**Fig 6:**
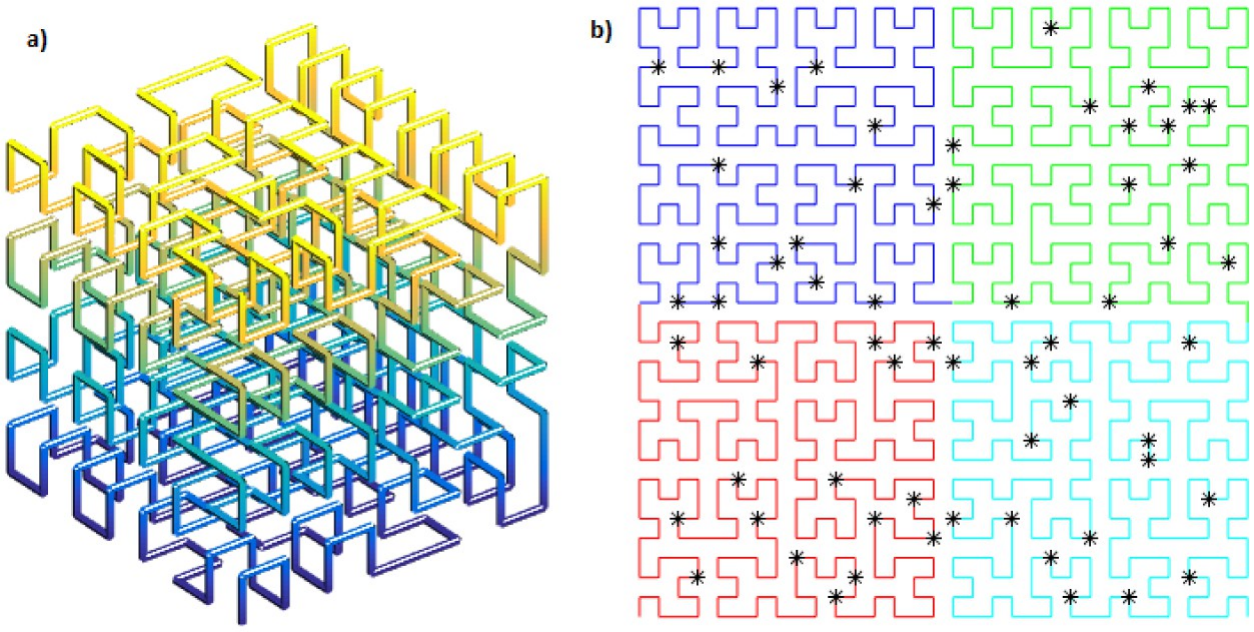
Examples of the Hilbert curves. (a) 3D Hilbert curve and (b) 2D Hilbert curve and particles randomly distributed in regions.

The space filling occurs in 2^*N*^ distinct regions, linked successively [60]. The number of points/vertices per region used by Hilbert curve to fill the space is given by $2^{N(n_{h}-1)}$, where *n*_*h*_ ≥ 2 is the *order of the Hilbert curve*, that is the degree of refinement or how close will the points be to each other. Fig 6a shows an example of the Hilbert curve in three-dimensions (3D). The 3D Hilbert curve presented is formed by 8 regions, distinguished by colors, with 64 points each. It’s easy to see that *N* = 3 and *n*_*h*_ = 3. The Fig 6b shows an example of the Hilbert curve in two-dimensions (2D). In this case we have *N* = 2 and *n*_*h*_ = 5, producing a curve in 4 regions, with different colors, and containing 256 vertices per region.

## The hybrid algorithm

The traditional PSO algorithm reaches more diversity when exploring the search space for a better global solution than several optimization methods. Nevertheless, it requires many particles in an ideal process which increases its computation time. Furthermore, it shows slow convergence when near the optimal solution, Kennedy [35]. On the other hand, the ANMS algorithm presents fast convergence but easily falls into a local optimum. Looking at the individual advantages of these two techniques leads us to believe that combining them can be a good hybrid optimization strategy. For example, the PSO could start as a global optimizer and then the ANMS would act as a local optimizer, directing the search process towards the optimum solution, as previously used by other authors [5, 7] and further explored here.

Thus, we now present a new approach, called PSO-Kmeans-ANMS, that consists of a hybrid algorithm with two stages, more precisely, a two-phase hybrid optimization technique. The first phase involves the PSO and the K-means algorithms. The particle swarm of the PSO is continuously supervised by the K-means algorithm, organizing the particles of the swarm in clusters. In this study, the swarm was divided in two clusters. As expected, at the end of this phase, the swarm (or a large part of it) will be attracted by an desired basin (attraction region), supposedly, containing the global optimum (minimum in our application). The second phase is due to the ANMS algorithm, in which a Simplex is built from the best points found by the PSO in the first phase. Again, it is expected, at this stage, a rapid convergence towards the optimum of that basin. Under these conditions, the ANMS technique is accurate and has one of the fastest convergence rates among the known direct search algorithms. We hope that the hybrid scheme presented here to be fast and accurate to converging to a desired optimal solution.

In this research, the K-means algorithm divides the swarm into *n*_*c*_ = 2 clusters at each iteration. The jump from Phase 1 (PSO) to Phase 2 (ANMS) takes place through the K-means and occurs when one of the clusters becomes dominant, i.e., much larger than the other. The size ratio measure between the clusters is given by *rel*_*s*_ and is a problem dependent factor. To avoid premature convergence of the PSO mod in Phase 1, the K-means only acts after 50% of the permitted iterations *It*_*max*_ have occurred. This is a restriction in terms of iteration (“time”), which, from there, allows the supervision by the K-means algorithm for a possible phase change of the hybrid optimization algorithm. The *T*_*Km*_, with *T*_*Km*_ = 0.5 × *It*_*max*_, is called the *K-means time*. Both 50% and *It*_*max*_ values are application dependent parameters.

Still in Phase 1 of the hybrid optimization, at some point of the iterative process after *T*_*Km*_, the swarm may achieve a very homogeneous particle distribution forming a “ball” that will contract during the next iterations. When this happens, the K-means algorithm may not be able to determine a dominant cluster, causing the PSO to go to the end of the permitted iterations. In an attempt to avoid this problem, a mechanism for monitoring the swarm homogeneity is triggered at each iteration. The monitoring is done by the ratio between the standard deviations of the particle fitness of the swarm at the current time, *std*(*A*), and that in the initial swarm, *std*(*AI*). If this ratio is less than or equal to a certain value, *rel* ≤ *rel*_*st*_, the change from Phase 1 to Phase 2 occurs. Where

$$rel=\frac{size\;of\;the\;largest\;cluster}{size\;of\;the\;smallest\;cluster}\;\text{or}\;rel=\frac{std(A)}{std(AI)},$$

and *rel*_*st*_ is a parameter that depends on the application. It was set, in the present research, to *rel*_*st*_ = 0.25.

In general terms, the PSO-Kmeans-ANMS iteratively tries to improve candidates’ solutions for a particular optimization problem, about a given objective function *f*(**X**), where each solution $X_{i}^{k}$ is represented by a particle *i* in the *N*-dimensional search space ℜ^*N*^ in the *k* order iteration. For Phase 1, the adaptive parameters (ranges) related to the PSO algorithm are inertia factor *w*, acceleration coefficients *C*_1_ and *C*_2_, the percentage of repulsive particles *rr*_*p*_, the limit allowed for the relationship between the standard deviations of the current, *rel*_*s*_, and the initial, *rel*_*st*_, swarms and the rebellion threshold *τ*. In addition, the fixed parameters *n*_*Pop*_, *It*_*max*_ and *η*, representing the size of the swarm population, the number of iterations allowed, and the percentage of velocity clamping, respectively, have to be set. For the K-means algorithm, it is necessary to define *n*_*c*_, the number of clusters in the particle swarm. For Phase 2, regarding the ANMS algorithm, the parameters are *ρ*, *χ*, *γ*, and *σ*, which correspond to the coefficients of reflection, expansion, contraction, and reduction, respectively. Other parameters in that phase are *β* and *α*_*s*_, with the first one being the step size to form the Simplex from a point *P*_1_, and the second, the tolerance for the stopping criterion. Finally, one should define *Ω*, *rel*_*s*_, and *T*_*Km*_, representing the hyper-box that defines the search space, the size ratio measure, and the K-means time, respectively.

The pseudocode of our PSO-Kmeans-ANMS algorithm, for optimizing an objective function *f*(**X**): ℜ^*N*^ → ℜ through iterations *k* = 1, 2, …, is presented below:

**Initial**
0 Initialization: Define *k* = 1; Use the Hilbert curve to randomly generate the position **X**^*k*^ and the velocity **V**^*k*^ = 0 for the entire swarm; Calculate the Velocity’s clamping,
  *V*_*j*,*max*_ = *η* × *range*_*j*_(*Ω*), *j* = 1, ⋯, *N*
**Phase 1**
1 Update *w*, *C*_1_, *C*_2_, *rr*_*p*_ and *τ* using the following equation adjusted to each one:

$S=S_{initial}-(\frac{It}{It_{max}})(S_{initial}-S_{final})$

2 Determine the number of rebel particles *n*_*reb*_:

$n_{reb}=\left(\frac{rr_{p}}{100}\right)\times n_{Pop}$

3 Determine the value for the variable *dir*:
   *dir* = 1
   If *rand* ≤ *τ*, do
    *dir* = −1
   End If
4 Best positions: for each particle *i* of the swarm,
   Calculates the value of fitness $fit\left(X_{i}^{k}\right)=f\left(X_{i}^{k}\right)$
   If $fit\left(X_{i}^{k}\right)<f\left(X_{kb}^{k}\right)$, do
    $X_{kb}^{k}=X_{i}^{k}$
   End If
   If $fit\left(X_{kb}^{k}\right)<f\left(X_{gb}^{k}\right)$, do
    $X_{gb}^{k}=X_{kb}^{k}$
   End If
5 Swarm update: for each particle of the swarm, update the velocity
   Considering the attraction between the particles by
   for *i* = 1, ⋯, (*n*_*Pop*_−*n*_*reb*_), do
    $V_{ij}^{k+1}=wV_{ij}^{k}+C_{1}r_{1}\left(X_{ib,j}^{k}–X_{ij}^{k}\right)+C_{2}r_{2}\left(X_{gb,j}^{k}-X_{ij}^{k}\right)$, *j* = 1, ⋯, *N*
   End For
   And, for the rebels particles, including repulsion or attraction forces as
   for *i* = (*n*_*Pop*_ − *n*_*reb*_) + 1, ⋯, *n*_*Pop*_, do
    $V_{ij}^{k+1}=wV_{ij}^{k}+dir\left[C_{1}r_{1}\left(X_{ib,j}^{k}–X_{ij}^{k}\right)+C_{2}r_{2}\left(X_{gb,j}^{k}-X_{ij}^{k}\right)\right]$, *j* = 1, ⋯, *N*
   End For
   Update the position of the all swarm using
    **X**^*k*+1^ = **X**^*k*^ + **V**^*k*+1^
6 Determine and select the *n*_*c*_ (two) clusters using the K-means algorithm:
   Apply K-means algorithm of the MATLAB Toolbox;
   Calculate $rel=\frac{std(A)}{std(AI)}$
   If *k* > *T*_*Km*_, do
    If *rel* ≥ *rel*_*s*_, do
     go to step 8 (stop phase 1 and go to phase 2)
    End If
    if *rel* ≤ *rel*_*st*_, do
     go to step 8 (stop phase 1 and go to phase 2)
    End If
   End If
   Otherwise, continue to step 7
7 Make *k* = *k* + 1 (the increment in iteration in phase 1). Then, do:
   if *k* ≤ *It*_*max*_, do
    return to step 1 (start a new iteration in phase 1)
   End If
   Otherwise, continue to step 8 (stop phase 1 and start phase 2).
**End of Phase 1**
**Phase 2**
8 Determine the *N* + 1 vertices of the Simplex **P**_*j*_ doing:
    $P_{1}=X_{gb}^{k}$
    **P**_*j* + 1_ = **P**_1_ + (0, ⋯, *β* × *range*_*j*_(*Ω*), ⋯, 0), *j* = 1, ⋯, *N*
9 Define *l* = 1 and apply the ANMS algorithm to the Simplex determined above until reaching the stopping criterion. I.e., for each iteration *l* of the ANMS, do:
   Calculate the standard deviation of fitness of the Simplex vertices, *std*(*fit*(**P**^*l*^))
   If *std* ≥ *α*_*s*_, do
    start a new iteration in ANMS in phase 2
    *l* = *l* + 1
   End If
   Otherwise, stop phase 2
**End of Phase 2**
**End Algorithm**

## Validation with benchmark functions

To validate the PSO-Kmeans-ANMS hybrid optimization algorithm, developed in this research, an individual analysis applied to 12 reference functions was performed. We use functions with many different aspects, for example, functions with many local minima (Ackley function, Rastrigin function), basin-shaped (Sphere function), plate-shaped (Zakharov function), valley-shaped (Rosenbrock function), with steep peaks/falls (Michalewicz function, F12), funnel-shaped (F22) and others such as Beale function, Styblinski-Tang function, and Peaks function. Functions F2, F12 and F22 are found in the work of Abualigah *et al.* [64]. The others are collected on internet sites, such as: Virtual Library of Simulation Experiments [65].

Next, we detail the validation results of the Rosenbrock and Rastrigin functions. Five cases were studied, each one with different numbers of particles in the swarm, specifically *n*_*Pop*_ = 8, 12, 20, 28, 36. Descriptions and results of the all functions are shown for 36 particles in the S1 Appendix.

The Rosenbrock function, also known as the valley function, with 2D dimension is a unimodal non-convex function characterized by the existence of a single global minimum in an extensive parabolic valley, whose convergence to the minimum is difficult to find. That is, it is a very complex function due to the sensitivity to the initial kick which, if it is far from the minimum, can cause the method to diverge. Furthermore, no matter how small the distance to the minimizer is, it makes a considerable difference to the image value. The Rastrigin function with two dimensions is a multimodal nonlinear function characterized by the existence of a single global minimum and several local minima very close to each other, making it difficult to scan and search for the global optimum. That is, it has several basins of attraction whose points converge to these minima (attractors). In both mentioned functions, the function *f*(**X**) = *fit*(**X**), that is, it is equal to the objective function of the problem, while in the FWI it is expressed by *ϕ*(.) given by Eq (2).

The parameters adopted for the PSO in Phase 1 with *n*_*Pop*_ = 36, were the following:

- Maximum number of iterations, *It*_*max*_ = 54, that is, *It*_*max*_ = 1.5 × *n*_*Pop*_;
- Maximum number of iterations in phase 1 *T*_*Km*_ = 27, that is, *T*_*Km*_ = 0.5 × *It*_*max*_.
- Maximum number of evaluations, *It*_*max*_ × *n*_*Pop*_ = 1944.

All algorithms developed for the current work were coded in MATLAB R2017a. The simulations were run on a machine with an Intel(R) Core(TM) i5–7200U 2.50GHz processor and RAM memory capacity of 8GB. Although possible, the codes were not parallelized.

The initial and final values for each PSO parameter in all application are in the Table 1. At the end of Phase 1, we adopted the value of *β* corresponding to 10% of the range of the dimensional variables of the search space of the problem for the construction of the equilateral Simplex that will be used by the ANMS algorithm in Phase 2 of the proposed algorithm.

We adopted two stopping criteria in Phase 1: one based on the relationship between cluster sizes, *rel* ≥ *rel*_*s*_ = 4, and another on the relationship between the standard deviations of the objective function values referring to the initial and current swarms, *rel* ≤ *rel*_*st*_ = 0.25. We employ two stopping criteria for the ANMS algorithm at the end of Phase 2: one based on the maximum number of evaluations, 1944, and another on tolerance, *α*_*s*_ = 10^−4^.

To avoid premature convergence in Phase 1, we chose to check the stopping criteria only after half of the total iterations allowed. Remembering that these parameters are of type dependent problems.

**Fig 7:**
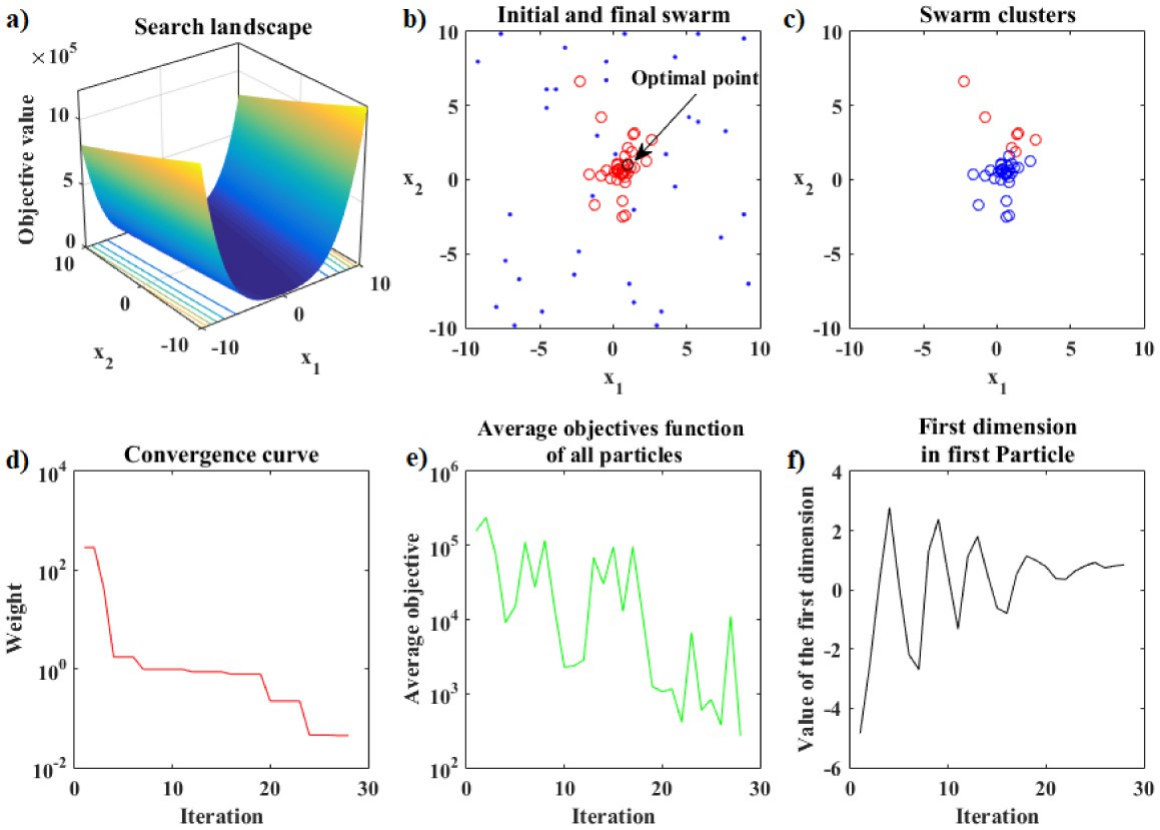
Rosenbrock function. Details of the optimization process of the PSO-Kmeans-ANMS algorithm in Phase 1 for the stopping criterion based on the ratio between the sizes of the clusters.

The performance analysis that follows refers to the Rosenbrook function. Fig 7 shows the details of the hybrid optimization process in Phase 1 of the PSO-Kmeans-ANMS algorithm. Fig 7a shows the appearance of the graph of the Rosenbrock function and the search space used. Fig 7b shows the initial swarm of particles, dots in blue, distributed by the Hilbert curve in the search space; and the final swarm, circles in red, found by the PSO. In addition, it also shows the level curves of the function and the exact optimal point, black circle. Fig 7c shows the end of Phase 1 with 28 of the 54 available iterations completed. At that moment, the K-means algorithm split the swarm into two clusters of particles. The largest cluster has 29 particles, blue circles, and the smallest has 7 particles, red circles. Therefore, the Phase 1 stopping criterion was met by the relationship between cluster sizes, that is, *rel*_*s*_ = 29/7 > 4. Fig 7d shows the convergence curve for the logarithm of the objective function value of the best particle in the swarm, at each iteration, given by the PSO in Phase 1. Fig 7e presents the curve for the logarithm of the means between the objective function values for all particles in the swarm, at each iteration, given by the PSO in Phase 1. Due to the rebellious nature of some particles, the graph shows significant up-and-down fluctuations. Fig 7f shows the values of the first coordinate of the first particle of the swarm granted by the PSO in Phase 1 at each iteration.

**Fig 8:**
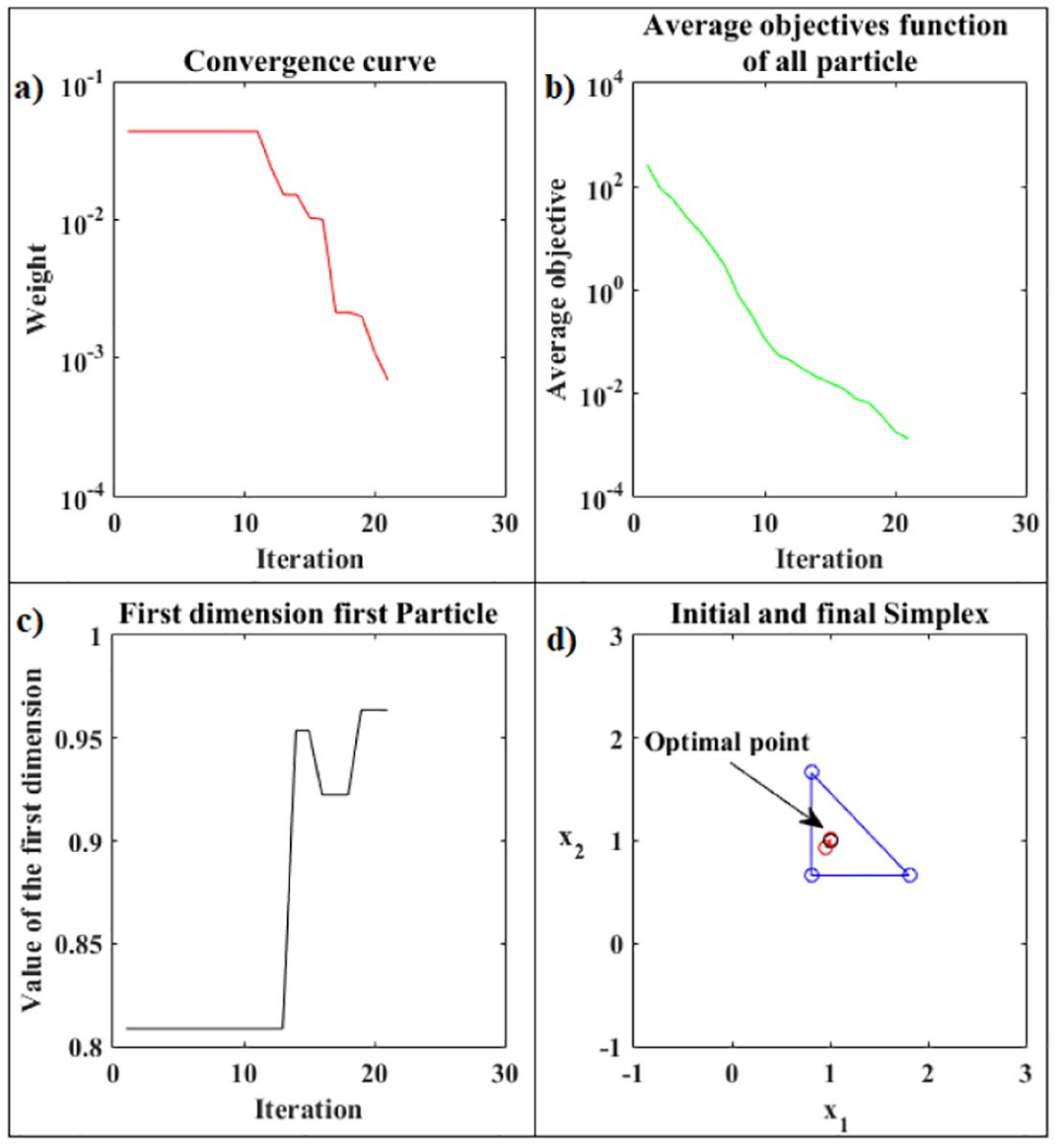
Rosenbrock function. Details of the optimization process of the PSO-Kmeans-ANMS algorithm in Phase 2.

Fig 8 presents the details of the hybrid optimization process proposed in Phase 2. Fig 8a shows the convergence curve for the logarithm of the objective function value of the best Simplex particle, at each iteration, obtained by ANMS in Phase 2. Fig 8b shows the curve for the logarithm of the means between the objective function values for all Simplex particles given by the ANMS in Phase 2 at each iteration. Fig 8c shows the values of the first coordinate of the first particle corresponding to the initial Simplex, at each iteration, given by the ANMS in Phase 2. Fig 8d shows the initial equilateral Simplex, blue circles, and final, red circles, found by the ANMS in Phase 2. Also shows the contour structure of the function and the exact optimal point, black circle. In Phase 2 the ANMS algorithm was performed with 64 of the 1944 available objective function evaluations.

Table 2 shows the details of the results found by the PSO-Kmeans-ANMS hybrid algorithm in the optimization of the Rosenbrock function. The most important part of the results is those concerning the reduction in the number of objective function evaluations and the precision of the optimal point. In the table we have a total of 1048 evaluations for the PSO-Kmeans-ANMS algorithm, being 1008 in Phase 1 and 40 in Phase 2, and good precision in the solution found. Compared with the 1944 evaluations available to be performed by the classic PSO, we have a reduction of 46.09% of the total evaluations. For objective functions that have a high computational cost, as in the case of FWI, this efficiency gain is good.

**Table 2:** Results of the PSO-Kmeans-ANMS algorithm for the Rosenbrock function. The number of objective function evaluations in Phases 1 and 2 corresponds, respectively, to 1008 and 40.

Parameters	Obtained	Exact
*x*_1_	1.0063	1.0
*x*_2_	1.0153	1.0
*f*(*x*_1_, *x*_2_)	6.96E-4	0.0

**Fig 9:**
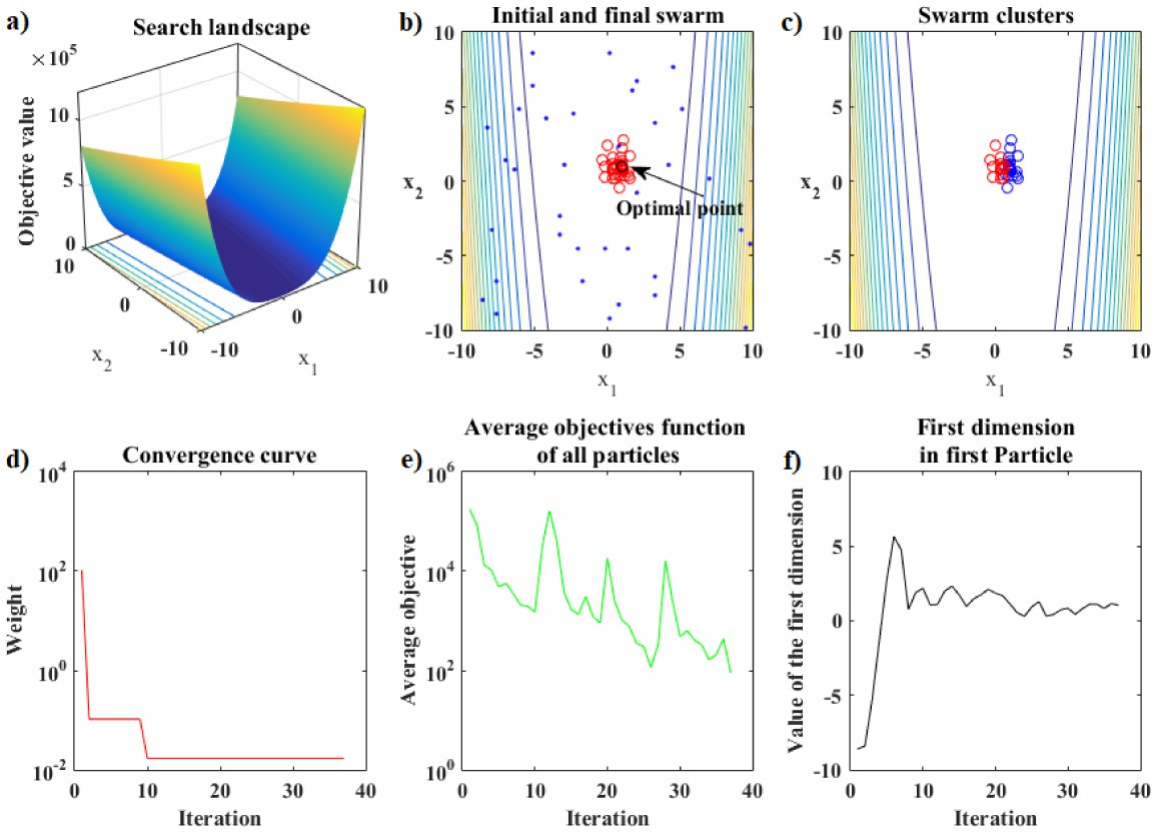
Rosenbrock function. Details of the hybrid optimization process in phase 1 for the stopping criterion based on the relationship between the standard deviations of the objective function values.

In Phase 1 of the hybrid algorithm, when the swarm forms a very homogeneous cluster of particles, the stopping criterion based on the ratio between the sizes of the clusters may fail. Fig 9 shows this situation. We can see in Fig 9c that the two clusters are similar and appear to be parts of a single cluster. In this case, it is difficult to decide on a dominant cluster. In this simulation, K-means divided the swarm into two clusters, one with 22 and the other with 14 particles that are represented, respectively, by the blue and red circles in Fig 9c. That is, the size criterion was not met, *rel*_*s*_ = 22/14 < 4. However, the stopping criterion based on the relationship between the standard deviations was reached, *rel*_*st*_ = (1.37 × 10^2^)/(2.90 × 10^5^) < 0.25.

The stopping criterion based on the ratio between the sizes of the clusters is more interesting for functions with local minima, such as the Rastrigin function. The criterion based on the relationship between standard deviations, for smoother functions that have only a local minimum, such as the Rosenbrock function.

In the optimization simulation of the Rastrigin function by the PSO-Kmeans-ANMS algorithm, the same parameters were used, but we adopted the value of *β* corresponding to 5%. This is because the range of dimensional variables has been reduced by half. As the function is multimodal, the stopping criterion in Phase 1 of the algorithm is, preferably, based on the ratio between the sizes of the clusters.

**Fig 10:**
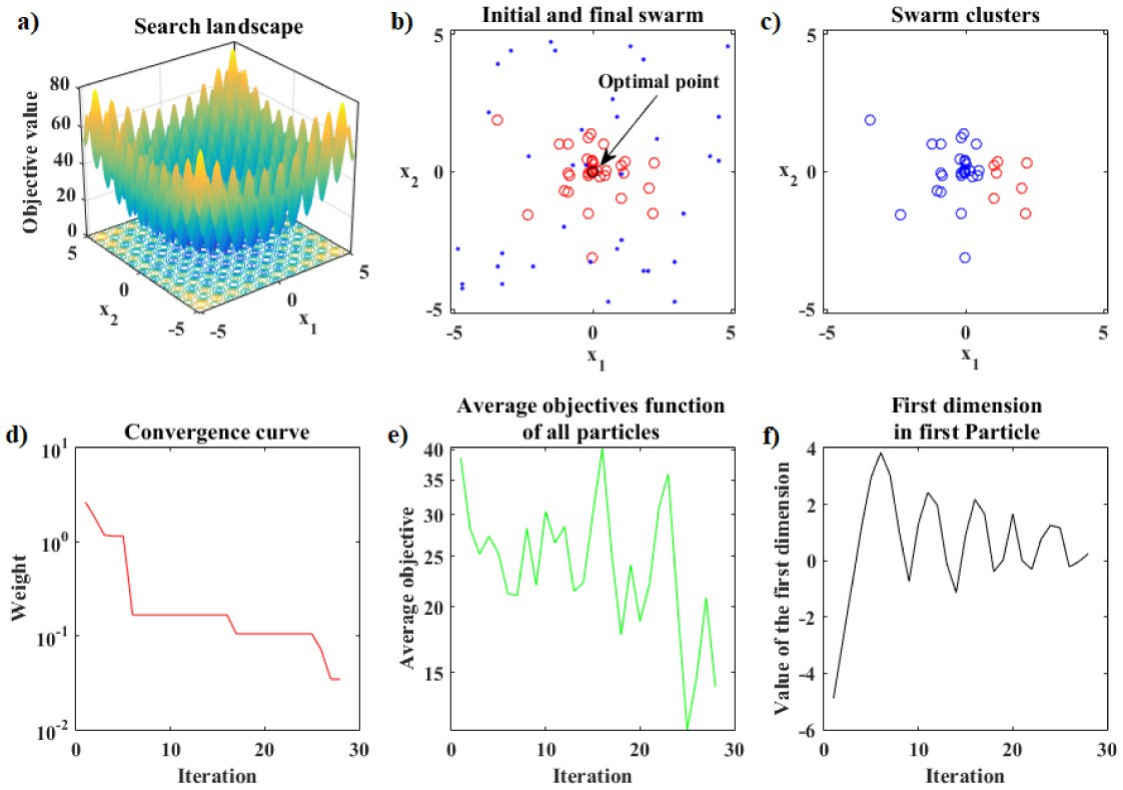
Rastrigin function. Details of the optimization process of the PSO-Kmeans-ANMS algorithm in Phase 1 for the stopping criterion based on the ratio between the sizes of the clusters.

**Fig 11:**
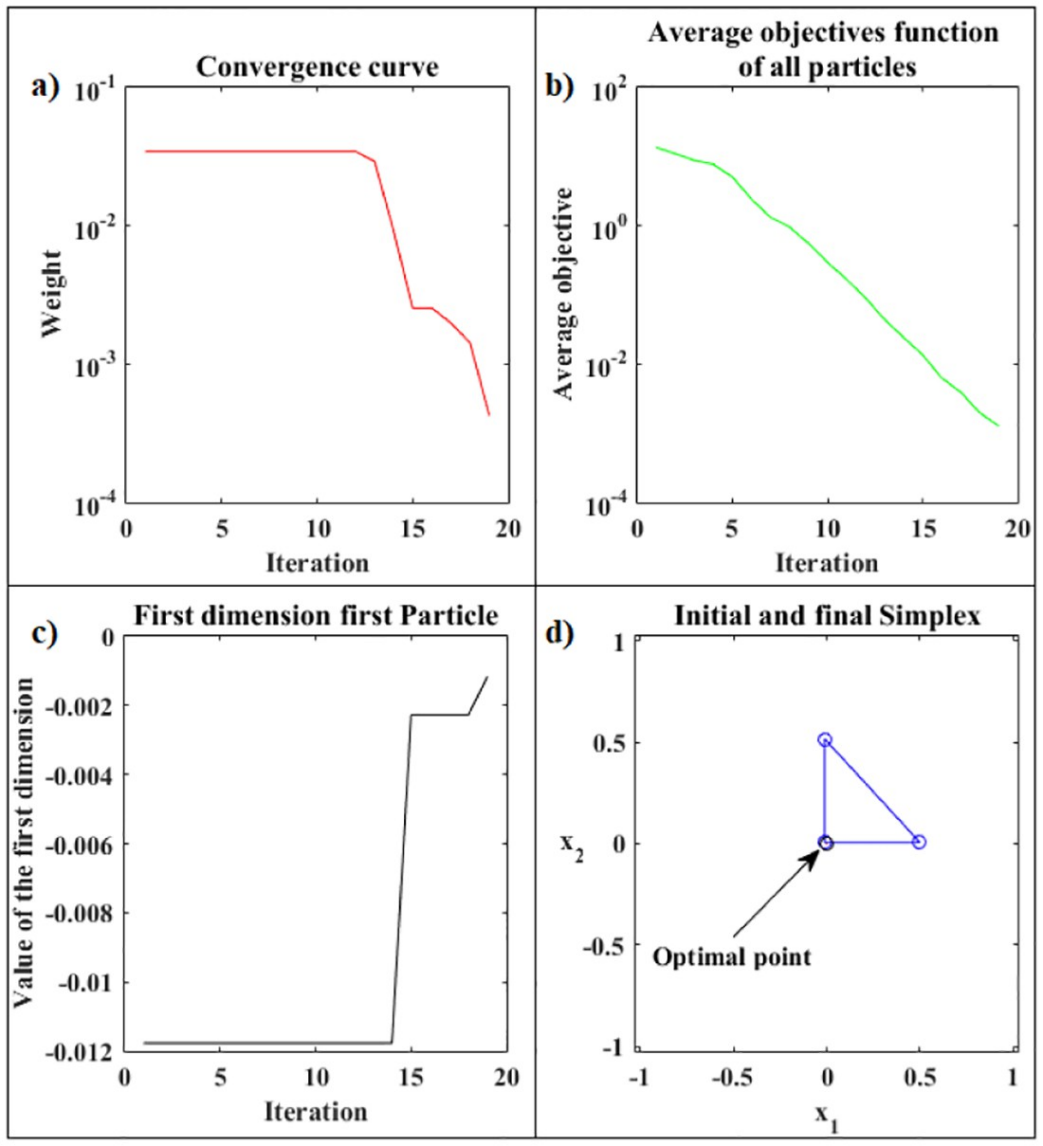
Rastrigin function. Details of the optimization process of the PSO-Kmeans-ANMS algorithm in Phase 2.

Figs 10 and 11 show, respectively, the details of the hybrid optimization process in Phase 1 and 2 of the PSO-Kmeans-ANMS algorithm, similar to those presented for the Rosenbrock function.

Table 3 presents the details of the results found by simulating in the optimization of the Rastrigin function by the PSO-Kmeans-ANMS hybrid algorithm. This table shows that we have a total of 1044 evaluations for the PSO-Kmeans-ANMS algorithm, with 1008 in Phase 1 and 36 in Phase 2, and a good precision in the solution found. Compared with the 1944 evaluations that should be carried out by the PSO classic, we have a reduction of 46.30% of the total evaluations. Therefore, a result similar to what was obtained when we used the Rosenbrock function in the optimization process.

**Table 3:** Results of the PSO-Kmeans-ANMS algorithm for the Rastrigin function. The number of objective function evaluations in Phases 1 and 2 corresponds, respectively, to 1008 and 36.

Parameters	Obtained	Exact
*x*_1_	-1.1609E-3	0.0
*x*_2_	8.9147E-4	0.0
*f*(*x*_1_, *x*_2_)	4.25E-4	0.0

Analysis of the influence of the *n*_*Pop*_ swarm size on the performance of the ANMS, PSO classic, PSO mod, and PSO-Kmeans-ANMS algorithms studied in this research for the two chosen reference functions: the Rosenbrock function, monomodal, and the Rastrigin function, multimodal. 100 simulations were performed for each algorithm. Tables 4 and 5 presents the percentage success rate for each algorithm referring to the swarm sizes with 8, 12, 20, 28, and 36 particles. The results show that all algorithms obtained a better response as the swarm size increased, with the exception of ANMS which showed adverse fluctuations in its results with respect to the multimodal Rastrigin function. Furthermore, the PSO-Kmeans-ANMS hybrid optimization algorithm, developed in this research, was the most successful in all situations considered here. The results also show that the hybrid approach becomes more competitive as the swarm shrinks in size. Which is a good result when dealing with high computational cost objective function problems, as in the case of FWI.

**Table 4:** Population *versus* success rate for the Rosenbrock function. Percentage success rate *sr*(%) for each algorithm referring to swarm sizes with 8, 12, 20, 28 and 36 particles.

*n*_*Pop*_	Algorithms			
	ANMS	PSO classic	PSO mod	PSO-Kmeans-ANMS
8	98	32	28	87
12	99	40	43	99
20	98	84	86	100
28	100	99	96	100
36	100	100	100	100

**Table 5:** Population *versus* success rate for the Rastrigin function. Percentage success rate *sr*(%) for each algorithm referring to swarm sizes with 8, 12, 20, 28 and 36 particles.

*n*_*Pop*_	Algorithms			
	ANMS	PSO classic	PSO mod	PSO-Kmeans-ANMS
8	4	17	25	77
12	40	40	31	99
20	60	79	58	100
28	9	96	92	100
36	9	98	99	100

**Fig 12:**
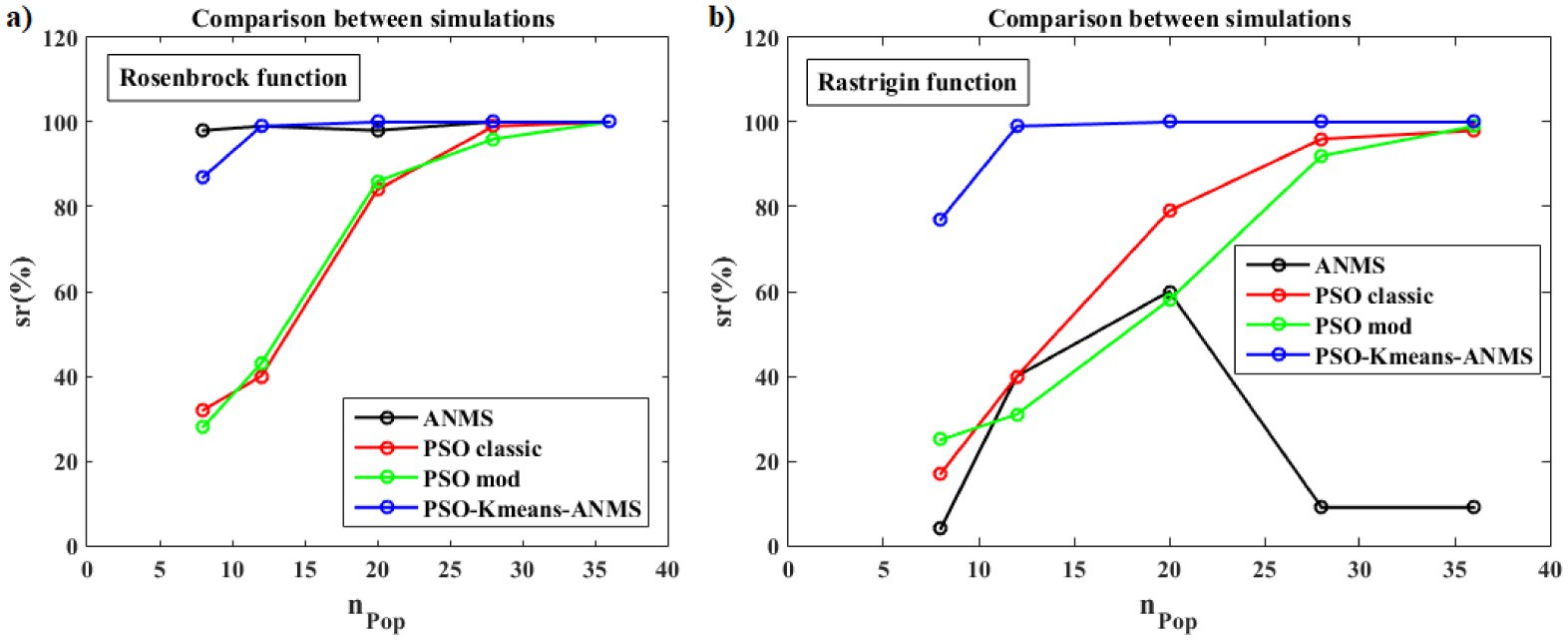
Comparison between simulations for all algorithms (success rate *versus* number of swarm particles). (a) Rosenbrock function and (b) Rastrigin function.

Fig 12 shows the graphic visualization of the results presented in Tables 4 and 5 for all analyzed algorithms.

**Fig 13:**
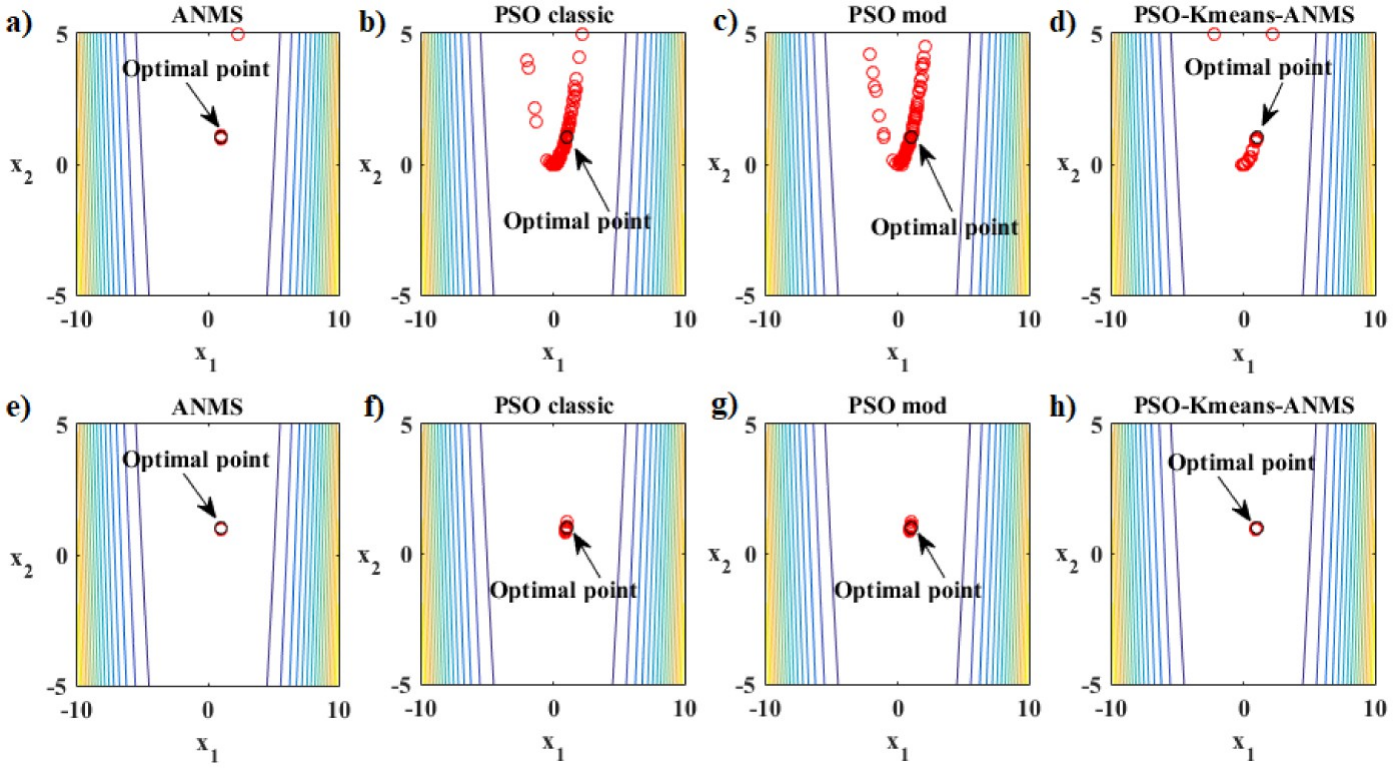
Simulations details for the Rosenbrock function. The 100 results obtained by the algorithms ANMS, PSO classic, PSO mod and PSO-Kmeans-ANMS for the swarms with 08 (upper) and 36 (lower) particles, respectively.

**Fig 14:**
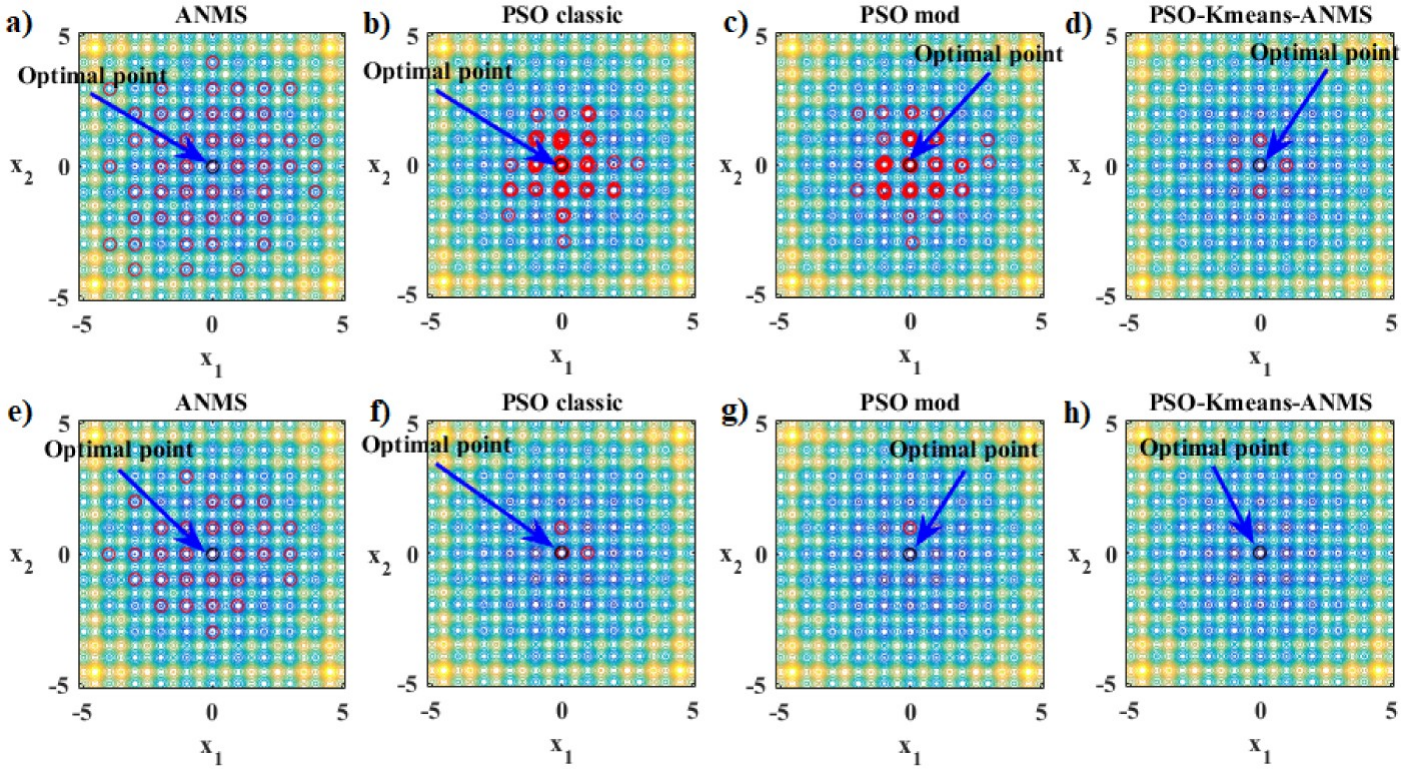
Simulations details for the Rastrigin function. The 100 results obtained by the algorithms ANMS, PSO classic, PSO mod and PSO-Kmeans-ANMS for the swarms with 08 (upper) and 36 (lower) particles, respectively.

Figs 13 and 14 show the final configurations of the particles, red circles, of the swarm after the optimization of the individually analyzed functions, Rosenbrock and Rastrigin, respectively. These settings correspond to the 100 results obtained by the algorithms ANMS, PSO, PSO mod and PSO-Kmeans-ANMS for the sizes of swarms with 08 and 36 particles.

## 1D FWI application

In order to demonstrate the efficiency and accuracy of the hybrid PSO-Kmeans-ANMS algorithm, we consider the non-linear problem of Full Waveform Inversion (FWI) of a simple synthetic model in one dimension with a profile consisting of only two layers. This may seem a trivial problem but, in the context of the inversion of mixed parameters, interface position (reflectors), and velocity of the layers, it is a considerable challenge to solve with high precision. A solution obtained by derivative-based optimization algorithms can, therefore, be costly, which motivates the usage of DFO-based optimization as an alternative.

The current section starts by describing the characteristics of the true model employed in the experiments and its parameterization strategy, focused on having just a few parameters. We then present the forward modeling and the seismic inversion process with their configuration parameters. Next, the parameters of the hybrid algorithm for this specific application, as well as the used hardware are discussed. Finally, we present the results of the experiments and comment on the main findings.

### Model parameterization

**Fig 15:**
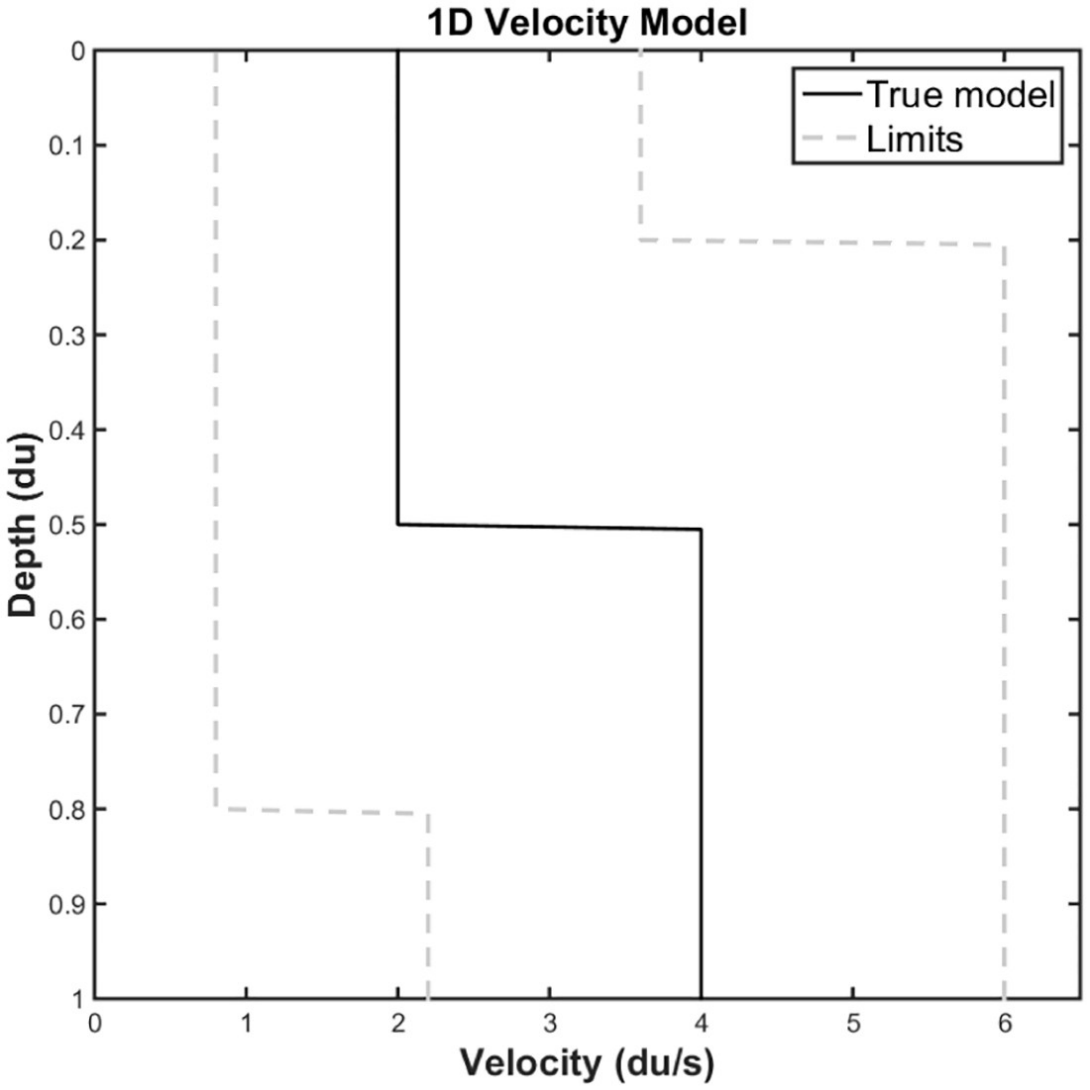
1D seismic velocity model. The solid black line is the true model, where the step indicates the position of the reflector. The dashed gray lines delimit the overall search space for the model parameters.

In an acoustic approximation of the seismic wave, where the density is considered constant, the kinematic property of the medium to be discovered in the inversion process is the velocity (variable *c* in Eq (5)). The *reference velocity model*, or *true model*, is a one-dimensional (1D) layered model represented by the profile of unit length *L*_*m*_ = 1.0 in a dimensionless unit (*du*). This is shown as a solid black line in Fig 15. The true model is formed by two layers with velocities of *V*_1_ = 2.0 and *V*_2_ = 4.0 of dimensionless unit per second (*du*/*s*). The interface between the two layers (the reflector) is located at *h*_*rf*_ = 0.5 *du*. We name this model just as *VH*, in reference to its velocity layers and the location of the interface. Given that each layer has its respective constant velocity and the position of the reflector is well-defined, we can parameterize the entire model with just these three values.

One should note that, due to the curse of dimensionality in global optimization, it is necessary to use parameterization strategies for reducing the number of parameters in the models under study [66]. A parameterization is capable of significantly reducing the total number of model parameters, and thus decreasing the dimension of the search space, what contributes to find a solution to the problem with less computational cost and in a more accurate manner. As investigated by Aguiar *et al.* [67] and Gomes *et al.* [68], a good parameterization can be obtained by focusing on layers. The parameterization described just above, in terms of the velocity of the layers and the position of the interface between them, is intuitive and provides a precise representation of a particular layered model.

Specializing the definition in Eq (4), we build a parameterization operator **Γ** that converts the VH model parameters **m** = (*m*_1_ = *V*_1_, *m*_2_ = *V*_2_, *m*_3_ = *h*_*rf*_) into a velocity model. That is, in a vector of size *nx* required by the forward modeling, as shown in Fig 2. In the limit case, when the parameters correspond to the reference model (correct values in Tabel 6), this operator leads to building the same true model, Fig 15.

**Table 6:** True model parameter and constraints. The exact values of the parameters *V*_1_, *V*_2_ and *h*_*rf*_ for true model and their lower (*L*_*low*_) and upper (*L*_*up*_) limits.

Values	Parameters		
	*V*_1_ (*du*/*s*)	*V*_2_ (*du*/*s*)	*h*_*rf*_ (*du*)
Exact	2.0	4.0	0.5
*L*_*low*_	0.8	2.2	0.2
*L*_*up*_	3.6	6.0	0.8

The possible values for a VH model can be constrained by some limits. Such limits depend on the nature of the problem and also represent parameters to be set. For this specific work, we defined those constraints as shown in Table 6, with the lower (*L*_*low*_) and upper (*L*_*up*_) limits imposed for each one of the parameters (*V*_1_, *V*_2_, *h*_*rf*_). In Fig 15, the dashed gray lines represent the possible models tested during the inversion process, that is, the limits imposed on the parameters in the search space.

These constraints restrict the parameter space for exploring possible models. The wider the range imposed by the limits is, the higher the computational effort for finding the optimal solution. However, if the limits are too narrow, there is a risk of leaving the desired optimal solution out of the search space. In terms of optimization, the model **m** can be considered a vector of decision variables.

Despite defining restrictions for the possible values in the parameter space, we can allow infeasible solutions to be generated. In that case, infeasible solutions (outside the limits) should be brought to the edges of the hyper-box defined by the restrictions. This tends to increase search efficiency with reduced computational cost (CPU time).

### Synthetic seismic data and modeling

The synthetic seismic experiment was designed to obtain a reflection data with a recording time of *t*_*max*_ = 1.5 seconds. We used a seismic source located near to the surface of the model, at position *x*_*s*_ = 0.10 *du*, and a single receiver located at position *x*_*r*_ = 0.15 *du*. The signature of the seismic source was given by a Ricker wavelet [22] with peak frequency equal to 10 Hz. For a forward modeling, a discretization process was made using the Finite Difference Method (FDM). In the spatial domain, the FDM mesh had *nx* = 201 nodal points and an interval Δ*x* ≅ 0.005. In the time domain, the parameters of the FDM discretization (Δ*t* and *nt*) obey the CFL condition, Eq (18). Our Absorbing Boundary Conditions (ABC), modified as described previously in this paper, were employed to suppress unwanted reflections on the edges of the model.

**Fig 16:**
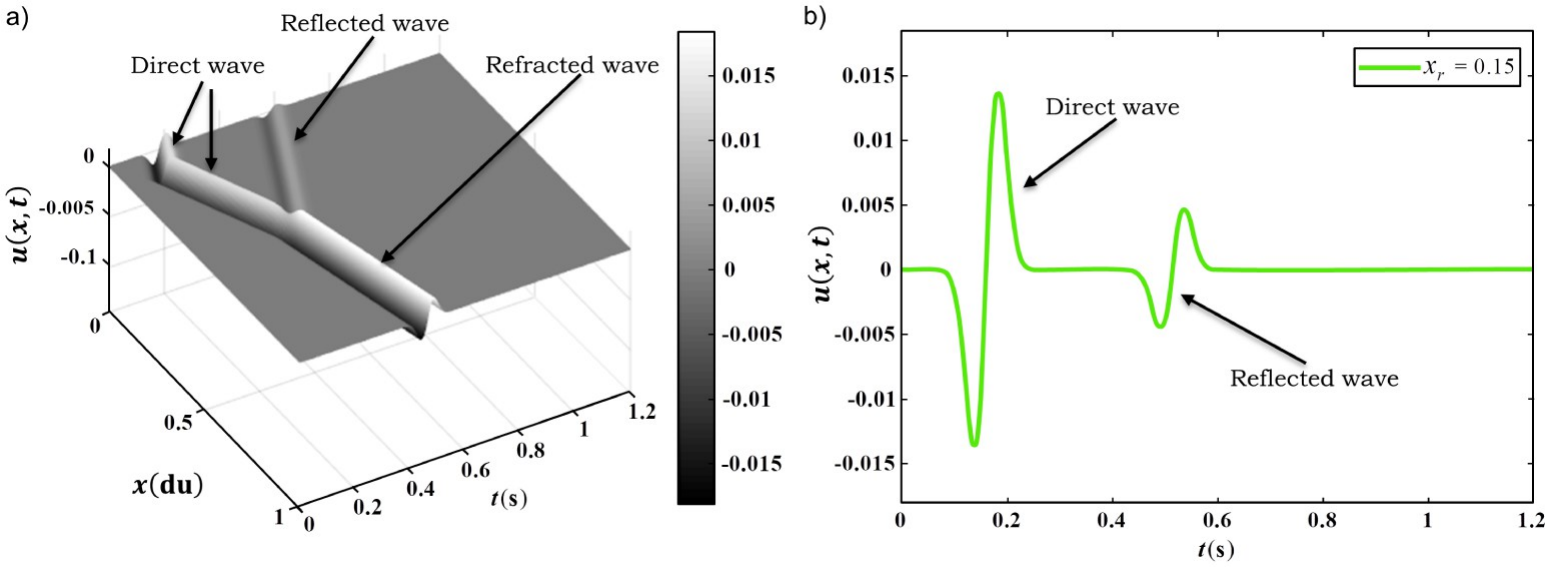
Wavefield propagation and observed data. (a) Evolution of wavefield propagation *u*(*x*, *t*) and (b) The observed data **d**^*obs*^ or synthetic seismic trace recorded at *x* = 0.15.

Fig 16a shows the wave propagation, that is, the evolution of the wavefield *u*(*x*, *t*) in function of time *t* and space *x*. Fig 16b presents the seismic trace (the seismogram) recorded by the receiver. In both images, it is possible to see two distinct events: (1) a large one, with the greatest amplitude, representing the wave signal that leaves the source and reaches directly the receiver, known as the direct wave; and (2) a more attenuated event, related to the reflection of the direct wave on the interface in the velocity model (according to Fig 15). In addition, we can see that the ABC treatment was highly precise, resulting in a recorded wavefield free of unwanted reflections at the edges.

### Hybrid optimization parameters, stop criteria and computational resources

In order to control the evolution of the PSO-Kmeans-ANMS algorithm towards the optimal solution, we carefully defined the maximum number of iterations allowed for simulation, *It*_*max*_, for both the PSO in Phase 1 and the NM in Phase 2. In addition, we set the number of clusters in which the K-means algorithm could divide the swarm of particles.

In this investigation, the K-means could divide the swarm of particles into two clusters at each iteration of the PSO in Phase 1. The jump from Phase 1 (PSO) to Phase 2 (ANMS) occurred when one of the clusters became dominant (four times larger than the other) or when *It*_*max*_ iterations were reached. For the NM method, we adopted a stop criterion of reaching either a Simplex shrinkage factor *α*_*s*_ = 10^−2^ or *It*_*max*_ iterations. We recall that *α*_*s*_ is based on the standard deviation of objective values of the solutions in the Simplex. The K-means only acted after 50% of the permitted iterations have occurred. Thus, the jump time was *T*_*Km*_ = 0.5 × *It*_*max*_. The value for *It*_*max*_ depends on the application and was set to 54. Furthermore, the step size coefficient of the ANMS was *β* = 0.5.

All algorithms developed for the current work were coded in MATLAB R2015a. The simulations were run on a machine with an Intel(R) Core(TM) i7 processor and RAM memory capacity of 16GB. Although possible, the codes were not parallelized.

The initial and final values for each PSO parameter are the same in validation (Table 1).

### Results and discussion

In this section, we present the results of the hybrid algorithm, PSO-Kmeans-ANMS and also its comparison to the PSO classic, PSO mod and the ANMS algorithms for the optimization of the misfit function between the observed seismic data (**d**^*obs*^) and the calculated seismic data (d*) in the context of the 1D FWI problem.

Remember that a solution to the FWI problem is described by the parameters of a velocity model, that is: **m** = (*m*_1_ = *V*_1_, *m*_2_ = *V*_2_, *m*_3_ = *h*_*rf*_). We studied three cases: Case 1, here referred to as 100 × 20, which performed 100 with 20 initial models (particles) each, therefore, composing 100 samples for analysis; Case 2, labeled 50 × 40, containing 50 samples with 40 initial models in each simulation; and Case 3, 100 × 10, with 100 samples and 10 initial models in each simulation. That is, we start with a swarm of 20 particles, double it to 40, and then divide it in half, 10. The intention for having such cases was to analyze the effect of the swarm size on the inversion process, FWI. We used the same strategy adopted in the numerical experiment with the benchmark functions to randomly generate the initial models in the simulations.

**Fig 17:**
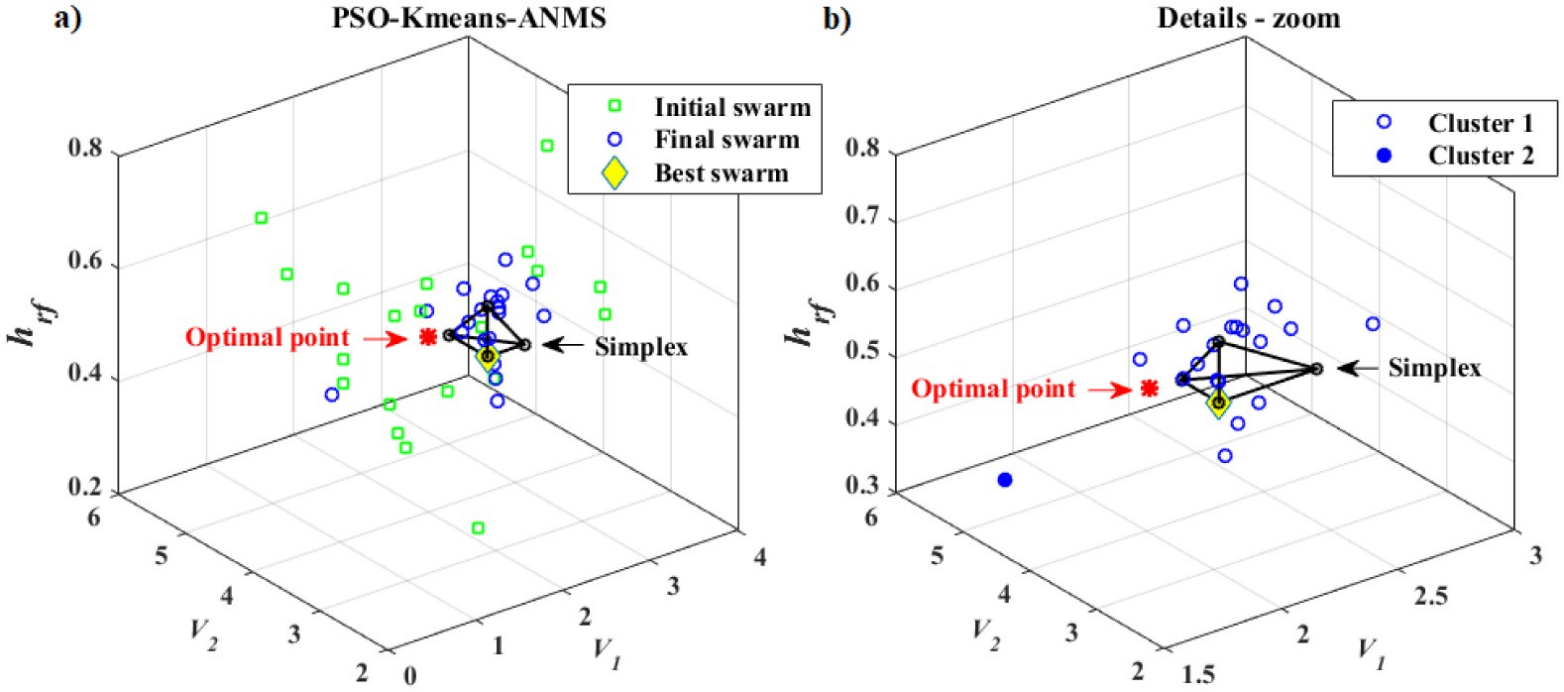
Case 1 (100 × 20). (a) The jumping moment from Phase 1 (PSO-Kmeans) for Phase 2 (ANMS). Represented by the color green (initial swarm), blue (final swarm), yellow (best solution for PSO) and red (optimal for ANMS) and (b) Details of Cluster 1 (empty blue circles), Cluster 2 (filled blue circles) and of the construction of the initial Simplex (black tetrahedron).

In order to illustrate how the PSO-Kmeans-ANMS algorithm works in the FWI problem, we take an example from the Case 1. The Fig 17 shows the evolution of the algorithm along its iterations. Precisely, in Fig 17a, we see the moment of jumping from Phase 1 (PSO-Kmeans) to Phase 2 (ANMS). In that figure, it is noticeable the shrinkage of the initial swarm (circles green) to the final swarm (circles blue). After the PSO-Kmeans-ANMS algorithm completing Phase 1, the K-means divided the final swarm (in blue) into two clusters, represented in the figure by empty and filled circles. In Phase 2, the ANMS built a quasi-regular initial Simplex (a tetrahedron in black) from the optimum point (in yellow) found by the PSO in the previous phase. Then, the tetrahedral Simplex was modified and shrunken by the ANMS algorithm allowing it to reach the global optimum (in red). This optimum is the model parameters **m** of the true model used in the FWI problem. Fig 17b shows, in detail, the Cluster 1, larger, with 19 particles, and the Cluster 2, smaller, with only 1 particle. The ratio between the cluster sizes is *rel* = 19/1 ≥ *rel*_*s*_ = 4, within the previously established criteria. Still, the dominant cluster is close to the optimum, making it easier for the ANMS to find that optimum.

**Fig 18:**
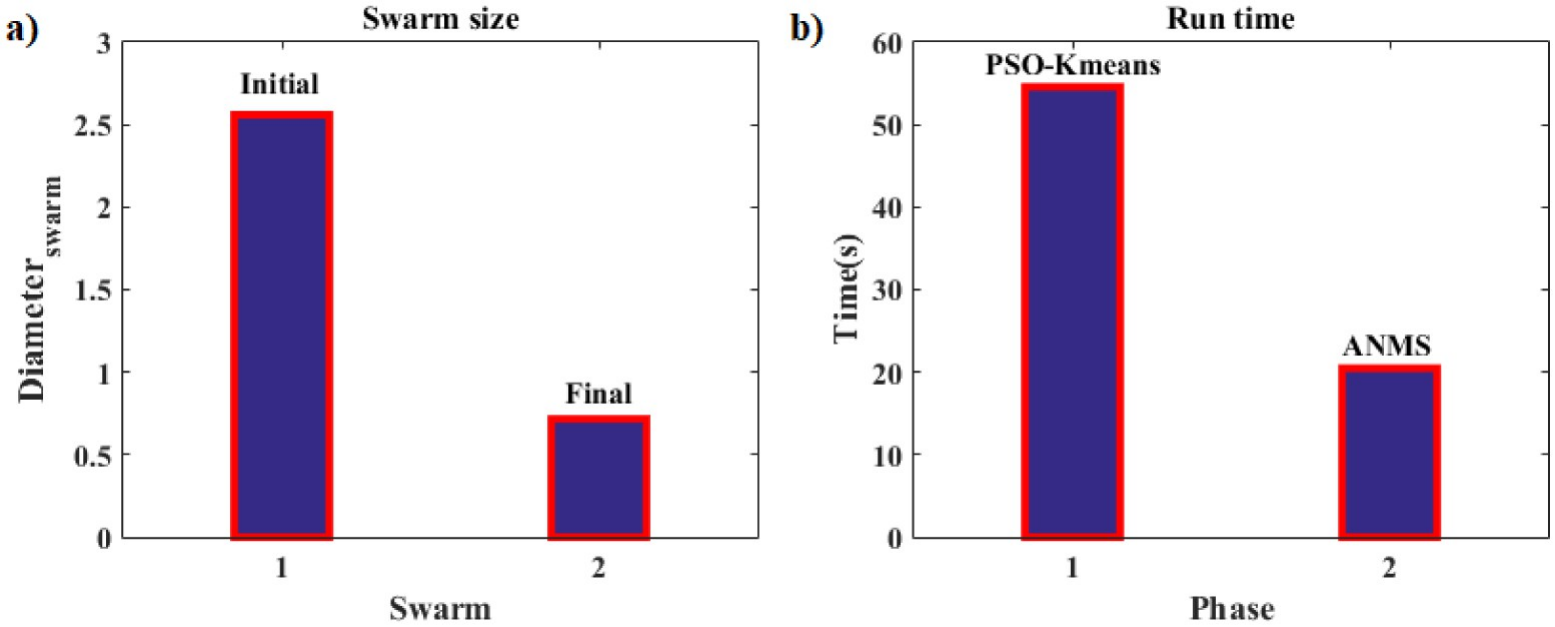
Case 1 (100 × 20). (a) Diameters of the initial and final swarms and (b) Run time of Phases 1 and 2.

Fig 18a shows how the swarm was shrunken in Phase 1. The shrinkage rate was 72%. The diameter of the swarm was calculated by averaging the distances of all particles to the swarm centroid. Fig 18b presents the run time (CPU time) spent in Phases 1 and 2. The ratio between them was 37%.

Now, we analyze the computer simulations for the three cases (100 × 20, 50 × 40, and 100 × 10) and discuss the most relevant information of the obtained samples (simulation results). Four approaches were considered, in all cases, to compare the optimization strategies, namely: a) only with the ANMS algorithm; b) only with the classic PSO algorithm; c) only with the modified PSO algorithm (with dynamic parameters), and d) with the hybrid PSO-Kmeans-ANMS algorithm. The algorithms are compared in terms of success rate (*sr*), the average execution time (*rt*), and the best result obtained for the objective function *ϕ*(**m**). As well as the average values of the model parameters (*V*_1_, *V*_2_ and *h*_*rf*_). For all samples, the solutions found by the optimization algorithms that varied from the real (exact) model in a range of ±4%, about their model parameters, were accepted as the global optimum.

First, we consider Case 1 (100 × 20), with its results presented in Table 7 for the four optimization algorithms studied. We can observe that the hybrid optimization approach obtained the highest success rate, 100%. Regarding the average execution time, the PSO-Kmeans-ANMS is in an intermediate position among the other algorithms. These results were already expected because the ANMS, a local search algorithm, converges faster than the PSO (classic or modified), a global search algorithm.

**Table 7:** Case 1 (100 × 20). Results of the success rate (*sr*) and average execution time (*rt*) of each optimization algorithm.

Values	Algorithm			
	ANMS	PSO classic	PSO mod	PSO-Kmeans-ANMS
*sr*(%)	48	99	99	100
*rt*(s)	72.3	148	157.2	106.6

Table 8 shows the result of the best model (*V*_1_, *V*_2_, *h*_*rf*_) obtained by each analyzed algorithm. We can see that the hybrid optimization approach obtained the best model with a misfit that is compatible with the other algorithms. The variables *V*_1_, *V*_2_, and *h*_*rf*_ seem to be less sensitive to the optimization process than the misfit.

**Table 8:** Case 1 (100 × 20). Results of model parameters and objective function (misfit) for the best solution obtained in each optimization algorithm.

Algorithm	Model Parameters			Objective Function
	*V*_1_ (2.0)	*V*_2_ (4.0)	*h*_*rf*_ (0.5)	*ϕ*(**m**) (0.0)
ANMS	2.0000	4.0000	0.5025	1.49×10^−4^
PSO classic	2.0000	4.0016	0.5016	3.98×10^−3^
PSO mod	2.0000	4.0000	0.5043	6.71×10^−5^
PSO-Kmeans-ANMS	2.0000	4.0000	0.5006	1.28×10^−4^

Table 9 shows the mean values obtained and their respective standard deviations for each parameter of the model. The average values of the CPU run time and of the objective function, misfit *ϕ*(**m**), obtained by each algorithm are presented in Table 10. Only successful models were computed in this analysis. We can see that all optimization algorithms achieved similar and very satisfactory results for variables *V*_1_, *V*_2_, *h*_*rf*_, and the misfit value *ϕ*(**m**). As for CPU time, the hybrid PSO-Kmeans-ANMS algorithm achieved a reduction of approximately 28% compared to the PSO classic and 32% compared to the modified PSO. This represents a significant gain when dealing with an optimization problem with a high computational cost objective function.

**Table 9:** Case 1 (100×20). Results of the mean value and the respective standard deviation for the parameters of the successful models.

Values	Algorithm		
	*V*_1_ (2.0)	*V*_2_ (4.0)	*h*_*rf*_ (0.5)
ANMS	2.01±4.08×10^−2^	4.01±5.73×10^−2^	0.504±8.03×10^−3^
PSO classic	2.00±9.34×10^−3^	4.00±3.35×10^−2^	0.502±2.31×10^−3^
PSO mod	2.00±1.40×10^−2^	4.00±2.48×10^−2^	0.503±3.37×10^−3^
PSO-Kmeans-ANMS	2.01±1.96×10^−2^	4.01±2.57×10^−2^	0.504±4.12×10^−3^

**Table 10:** Case 1 (100 × 20). Results of the mean value and the respective standard deviation for the CPU time and the objective function.

Algorithm	Run time	Objective function
ANMS	72.30±17.18	0.442±4.00×10^−1^
PSO classic	147.99±10.15	0.111±1.34×10^−1^
PSO mod	157.20±16.45	0.124±1.69×10^−1^
PSO-Kmeans-ANMS	106.58±11.80	0.194±2.28×10^−1^

**Fig 19:**
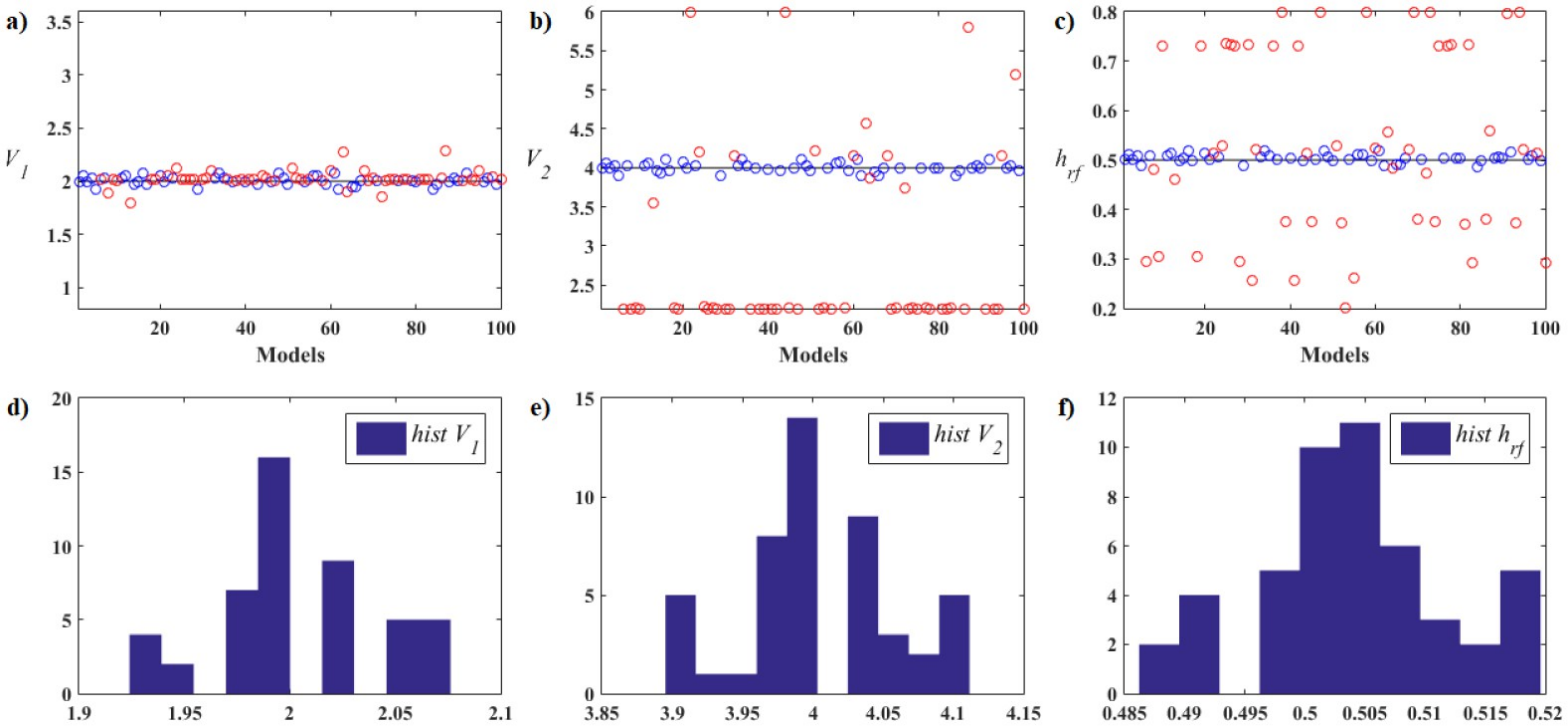
Case 1 (100 × 20). (a), (b), and (c) are all results obtained by the ANMS algorithm for variables *V*_1_, *V*_2_, and *h*_*rf*_, respectively. Successful models are highlighted in blue and unsuccessful ones are in red. (d), (e), and (f) are their respective histograms.

Fig 19a–19c show the results for the parameters of the velocity models computed by the ANMS optimization algorithm, distributed around the exact values *V*_1_ = 2.0, *V*_2_ = 4.0, and *h*_*rf*_ = 0.5, respectively. The blue circles represent the successful models, with values within the range defined for the optimal solution. The red circles indicate the models that were not successful. Fig 19d–19f show the corresponding histograms for each model parameter.

**Fig 20:**
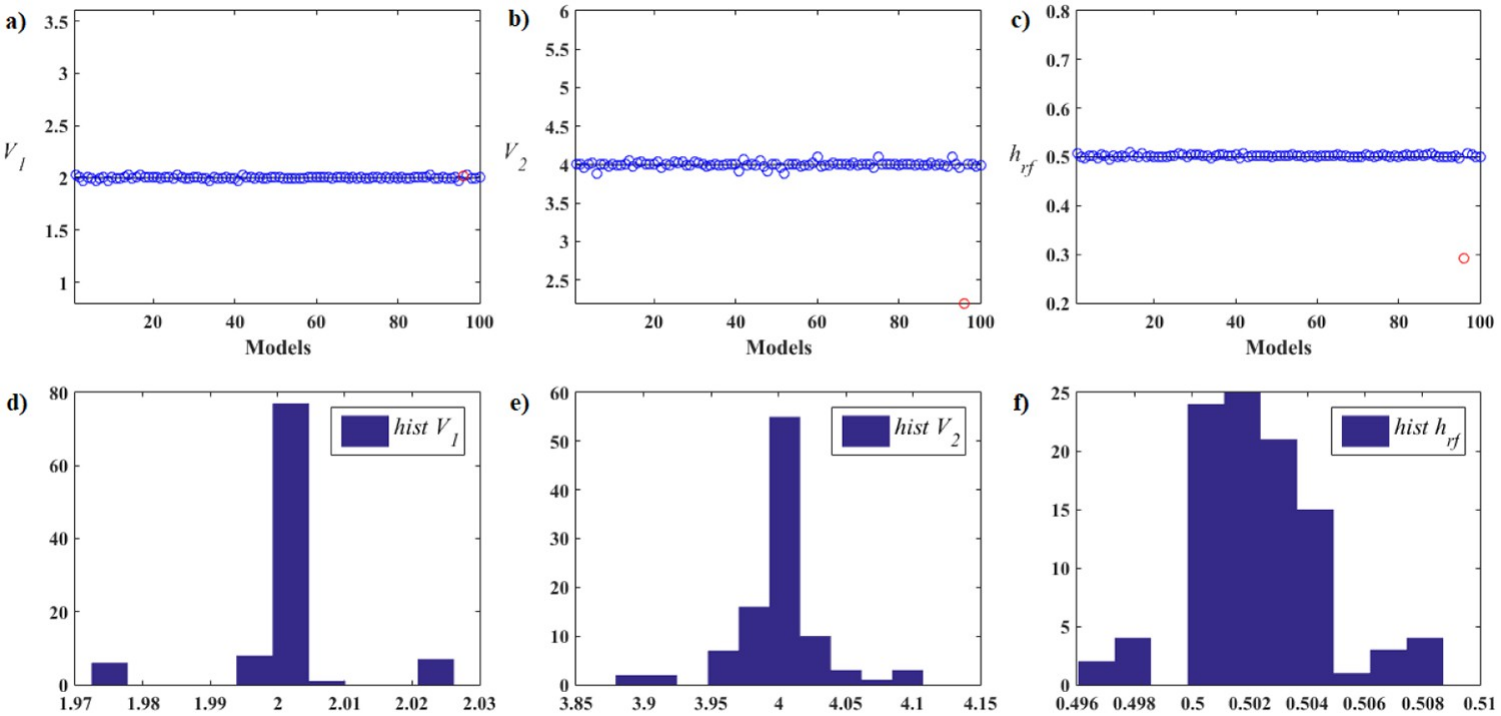
Case 1 (100 × 20). (a), (b), and (c) are all results obtained by the PSO classic algorithm for variables *V*_1_, *V*_2_, and *h*_*rf*_, respectively. (d), (e), and (f) are their respective histograms.

**Fig 21:**
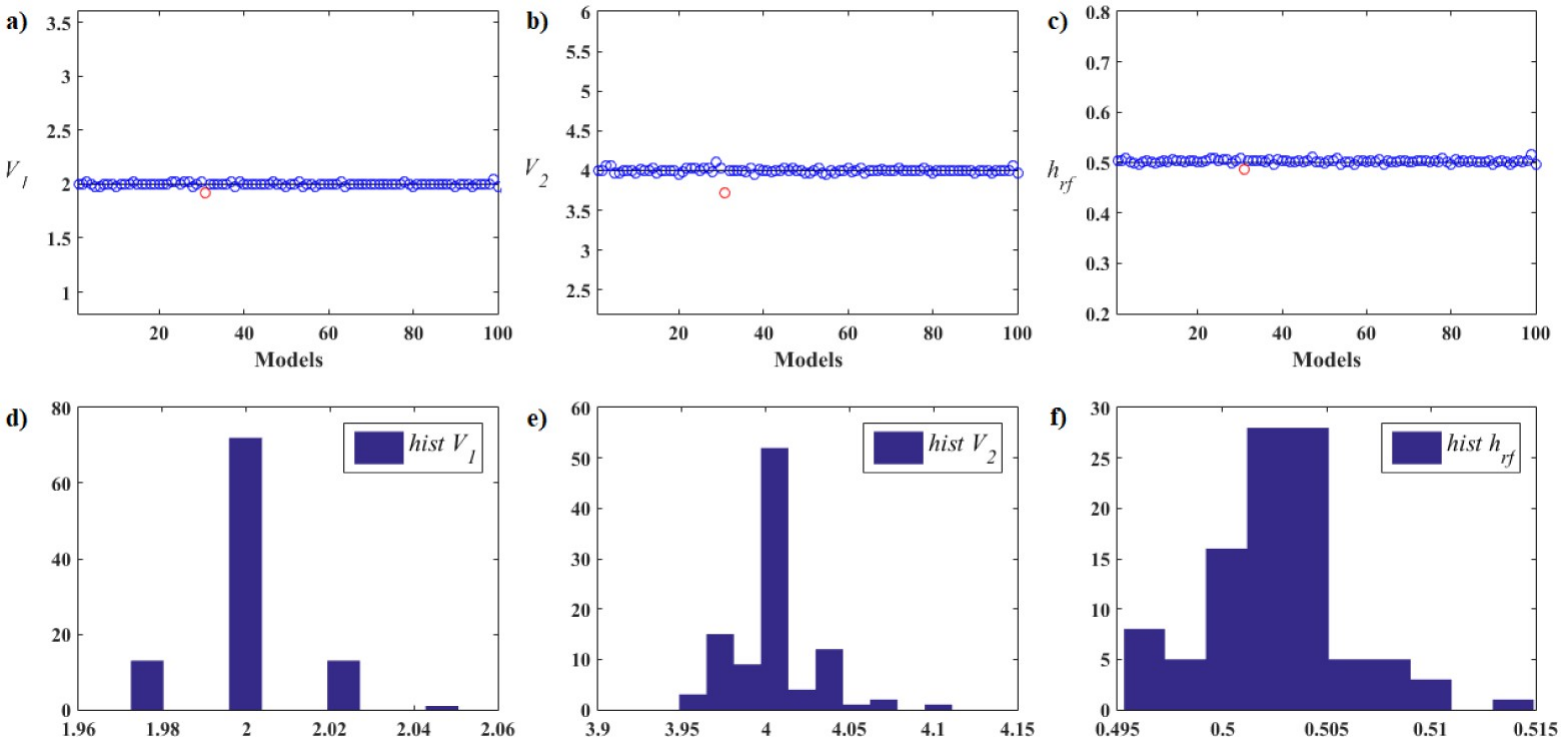
Case 1 (100 × 20). (a), (b), and (c) are all results obtained by the PSO mod algorithm for variables *V*_1_, *V*_2_, and *h*_*rf*_, respectively. (d), (e), and (f) are their respective histograms.

**Fig 22:**
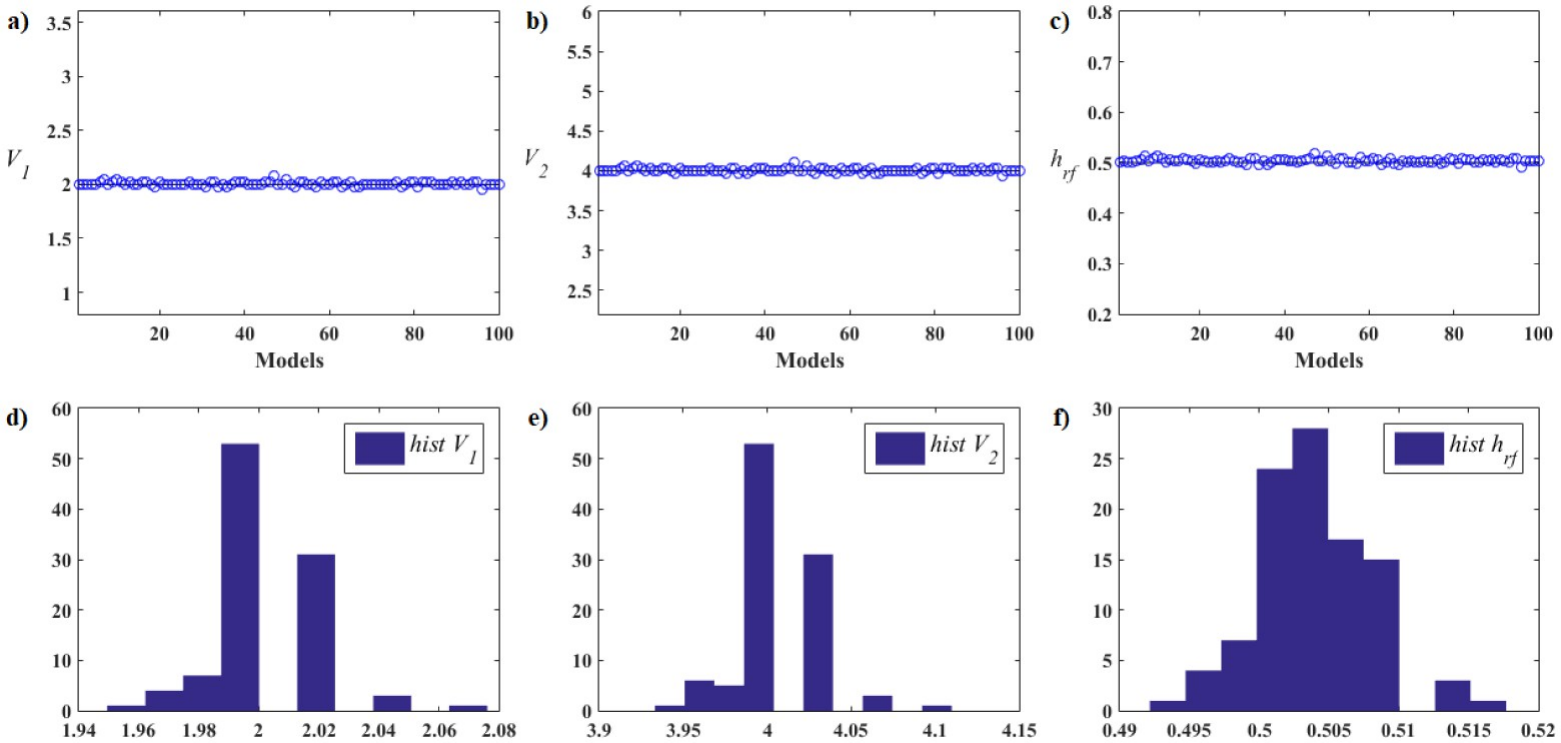
Case 1 (100 × 20). (a), (b), and (c) are all results obtained by the PSO-Kmeans-ANMS algorithm for variables *V*_1_, *V*_2_, and *h*_*rf*_, respectively. (d), (e), and (f) are their respective histograms.

Similarly, Figs 20–22 show the corresponding results for the parameters of the velocity models computed for the other PSO classic, PSO mod, and PSO-Kmeans-ANMS optimization algorithms, respectively.

**Fig 23:**
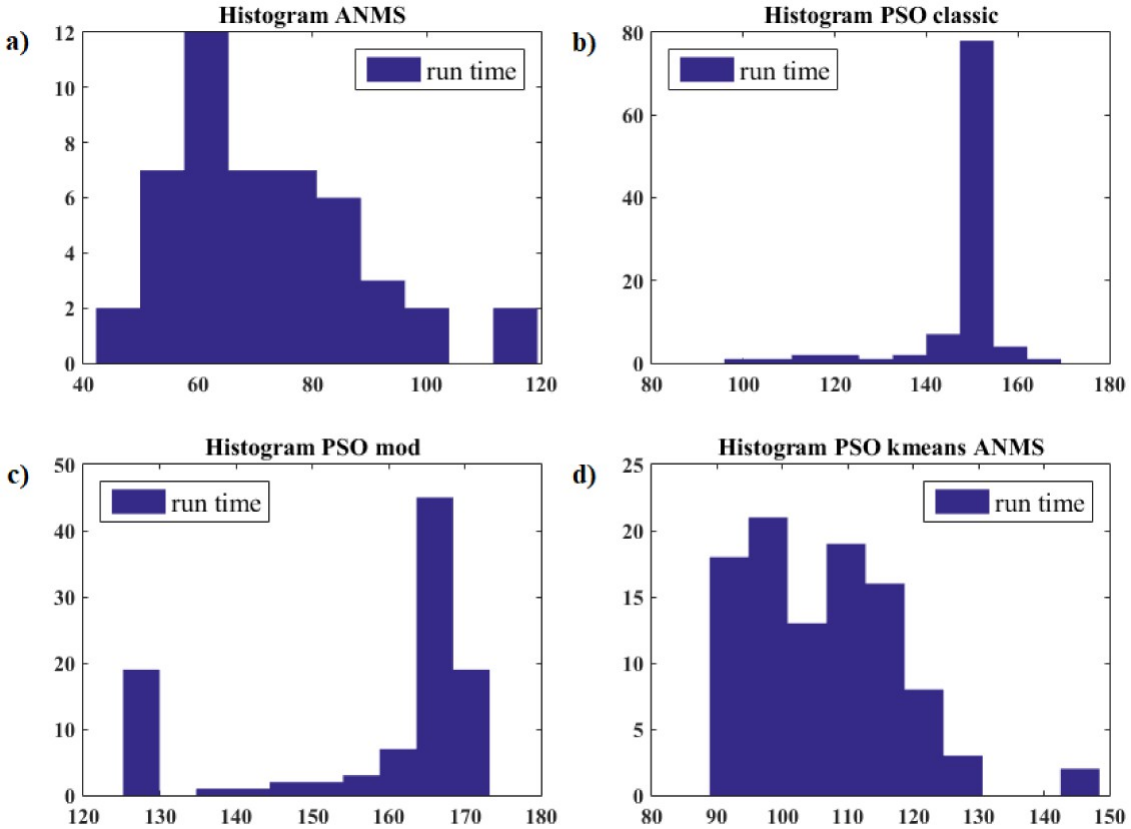
Case 1 (100 × 20). Histograms of the CPU time spent by the four optimization algorithms, ANMS, PSO classic, PSO mod, and PSO-Kmeans-ANMS, respectively. Only applied to successful models.

In the sequence, Fig 23 presents the run time histogram for each optimization approach. All histograms were constructed based on the analysis of successful models. And in them, it is possible to visualize the dispersion of values relative to the average value, shown in Tables 9 and 10.

**Fig 24:**
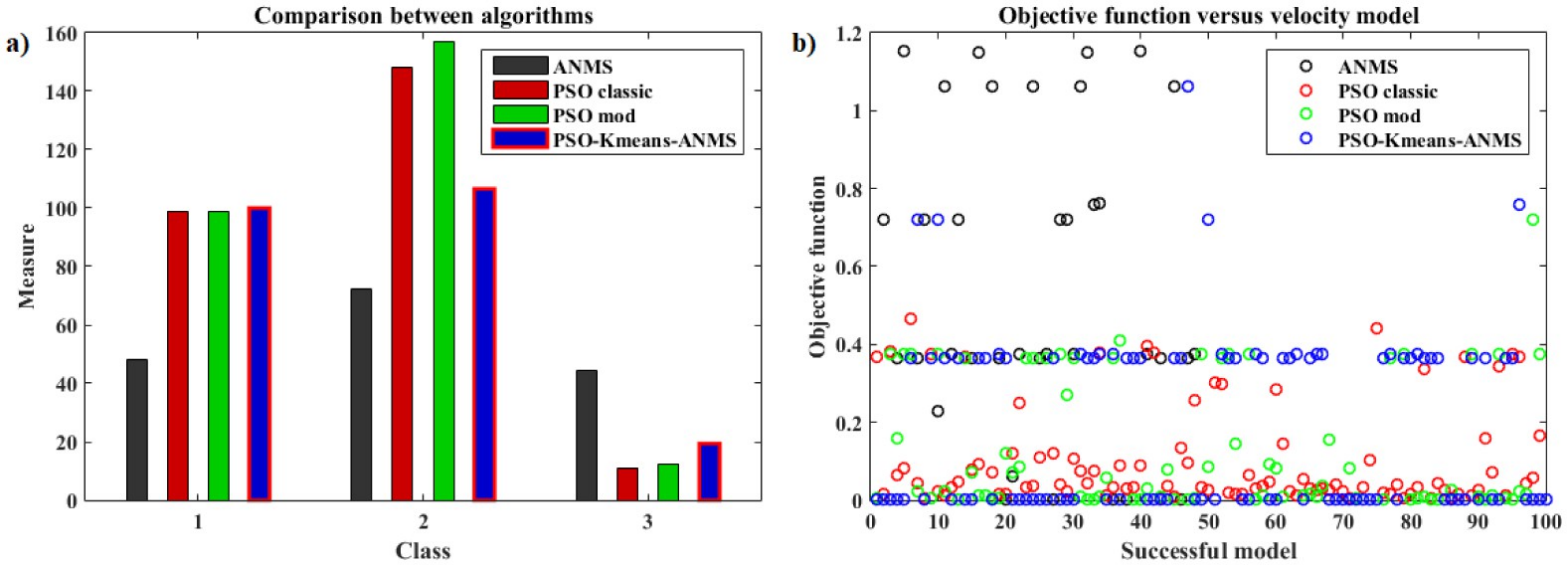
Case 1 (100 × 20). (a) Comparison between algorithms divided into: Class 1, success rates; Class 2, average CPU times and Class 3, average objective function values (*ϕ*(**m**) × 10^2^). (b) Objective function values for successful models.

Fig 24a presents a graphic summary referring to case 1 (100 x 20) for the FWI. This summary with the results obtained in the simulations corresponding to each optimization approach was grouped into value classes. Being Class 1, success rates (%); Class 2, average CPU times (in seconds) and Class 3, average misfits (*ϕ*(**m**) × 10^2^). Fig 24b shows the values obtained from the objective function only for the successful models.

From Figs 19, 20, 21 and 22, we can say that the successful models, in blue, are more concentrated around the exact solutions for the variables *V*_1_, *V*_2_, and *h*_*rf*_, but to a lesser extent for the ANMS algorithm. Such behavior is because ANMS is an efficient local optimizer, while PSO is a global optimizer that increases the chance of escaping from local minimums. As for the histograms, all of them presented little significant dispersions around the mean.

We can see in Fig 24a that the hybrid optimization approach, PSO-Kmeans-ANMS, achieved a success rate of 100% and managed to reduce the CPU time with the decrease of the PSO performance time. Thus, it reached a very acceptable objective function value. In Fig 24b we have a more detailed view of the values of these misfits for the successful models. According to the data, we can see that all algorithms presented values close to the minimum value of the objective function.

Now, we analyze the simulations for Case 2 (50 × 40), extracting more relevant data to compare with Case 1 (100 × 20). Similar conditions as in Case 1 were applied. The difference is in the size of the swarm, which has twice as many particles for Case 2, and in the reduction by half of the number of simulations. In Table 11, we have the success rate, *sr*; and the average execution time, *rt*, for each optimization approach used in this research.

**Table 11:** Case 2 (50 × 40). Results of the success rate (*sr*) and the average execution time (*rt*) for each optimization algorithm.

Value	Method			
	ANMS	PSO classic	PSO mod	PSO-Kmeans-ANMS
*sr*(%)	62	100	100	100
*rt*(s)	66.9	231	227.3	166.4

As shown in Table 11, for Case 2 (50 × 40), all optimization algorithms achieved a success rate of 100% except the ANMS algorithm. The PSO-Kmeans-ANMS maintained its success rate and the other approaches increased their values. In terms of execution time, PSO-Kmeans-ANMS was in an intermediate position concerning ANMS and the algorithms that make use of PSO. The hybrid algorithm maintained the reduction of 28% about the PSO, and as for the modified PSO, it presented an approximate decrease from 32% in Case 1 to 27% in Case 2. Table 12 shows the best results achieved by each optimization approach. Little significant changes are observed in the results obtained for these parameters. We also observed that with a larger swarm the four optimization approaches had better results. This seems to be intuitive, that is, the more particles we have, the greater the chance of the algorithm falling into a basin of attraction (converging). However, the run time grows significantly due to the increase in the number of objective function evaluations. This makes the simulation experiment more computationally expensive.

**Table 12:** Case 2 (50 × 40). Results of model parameters and the respective objective function (misfit) for the best solutions obtained in each optimization algorithm.

Algorithm	Model Parameters		Objective Function	
	*V*_1_ (2.0)	*V*_2_ (4.0)	*h*_*rf*_ (0.5)	*ϕ*(**m**) (0.0)
ANMS	2.0000	4.0000	0.5025	1.49 × 10^−4^
PSO classic	2.0000	4.0006	0.5017	1.83 × 10^−3^
PSO mod	2.0000	4.0000	0.5020	3.66 × 10^−5^
PSO-Kmeans-ANMS	2.0000	4.0000	0.5018	1.33 × 10^−4^

**Fig 25:**
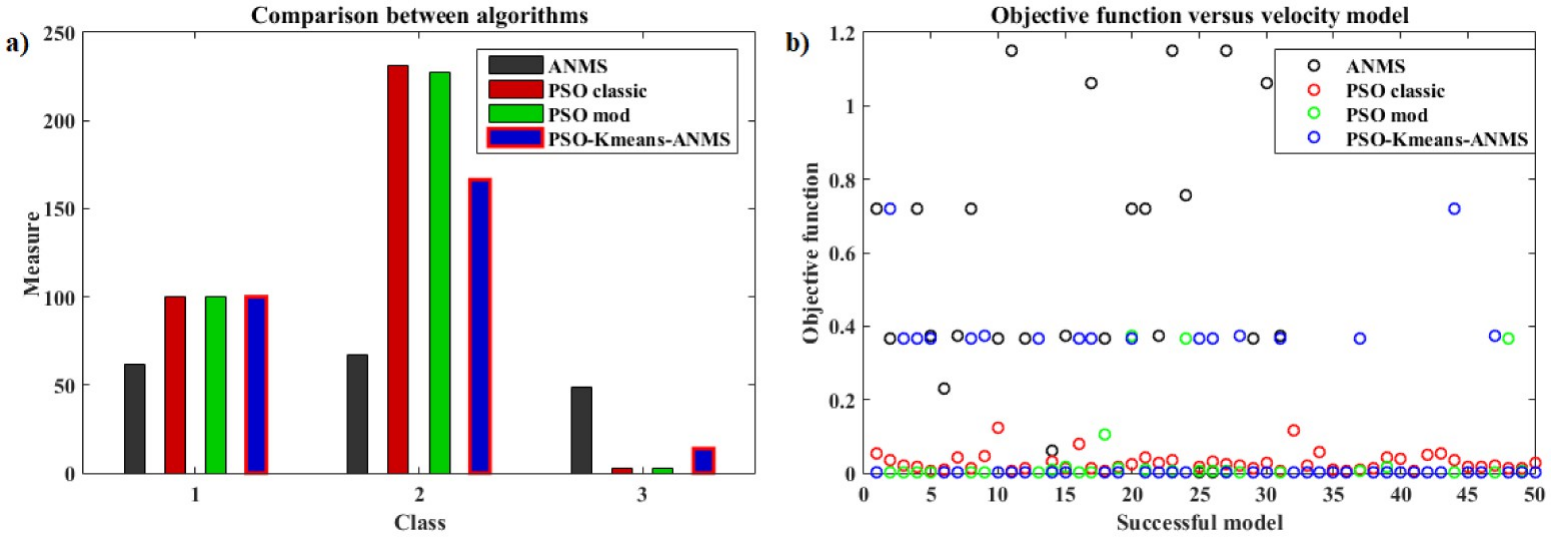
Case 2 (50 × 40). (a) Comparison between algorithms divided into: Class 1, success rates; Class 2, average CPU times and Class 3, average objective function values (*ϕ*(**m**) × 10^2^). (b) Objective function values for successful models.

Finally, Fig 25a shows the graphic summary referring to Case 2 (50 × 40) for the FWI. This summary of each optimization approach has been grouped into value classes. Fig 25b presents the values obtained by the objective function only for the successful models.

Also, based on Fig 25a similarly to Case 1 (100 × 20), the PSO-Kmeans-ANMS hybrid optimization algorithm maintained a success rate of 100% and was able to reduce CPU time with an objective function value very close to the exact minimum. In Fig 25b, although all the values of the misfits were kept in the same range of values, the PSO algorithms (classic or modified) presented a more uniform distribution and were very close to the exact value. Generally speaking, there was a gain when the swarm size was doubled, particularly for the PSO (classic or modified). However, there was a certain cost given by the significant increase in the run time of the optimization algorithms.

Now, we analyze the simulations of Case 3 (100 × 10) to obtain more relevant data to compare with Case 1 (100 × 20). Similar conditions to Case 1 were adopted. The only difference is in the size of the swarm which has half the particles for Case 1. In Table 13, we have the success rate, and the average execution time for each optimization approach used in this research.

**Table 13:** Case 3 (100 × 10). Results of the success rate (*sr*) and the average execution time (*rt*) for each optimization algorithm.

Value	Method			
	ANMS	PSO classic	PSO mod	PSO-Kmeans-ANMS
*sr*(%)	40	77	76	96
*rt*(s)	73	56.6	56.2	77.5

As shown in Table 13, for Case 3 (100 × 10), all optimization algorithms obtained a reduction in the success rate. However, the PSO-Kmeans-ANMS showed the smallest reduction, thus remaining in the lead. Regarding the run time, all optimization algorithms obtained a considerable reduction except ANMS, which practically maintained its value. The performance of PSO-Kmeans-ANMS in Phase 2 was impaired because of its dependence on ANMS.

Therefore, with fewer particles in the swarm, the hybrid algorithm managed to remain in a very comfortable situation regarding the success rate, although it lost CPU time. That is, there was a trade-off between the reduction in swarm size and CPU time.

Table 14 shows the best results achieved by each optimization approach. Little significant changes are observed in the results obtained for these parameters.

**Table 14:** Case 3 (100 × 10). Results of model parameters and the respective objective function (misfit) for the best solutions obtained in each optimization algorithm.

Algorithm	Model Parameters		Objective Function	
	*V*_1_ (2.0)	*V*_2_ (4.0)	*h*_*rf*_ (0.5)	*ϕ*(**m**) (0.0)
ANMS	2.0000	4.0000	0.5025	1.49×10^−4^
PSO classic	1.9999	4.0022	0.5043	7.38×10^−3^
PSO mod	2.0000	4.0002	0.5013	1.07×10^−3^
PSO-Kmeans-ANMS	2.0000	4.0000	0.5022	9.86×10^−5^

**Fig 26:**
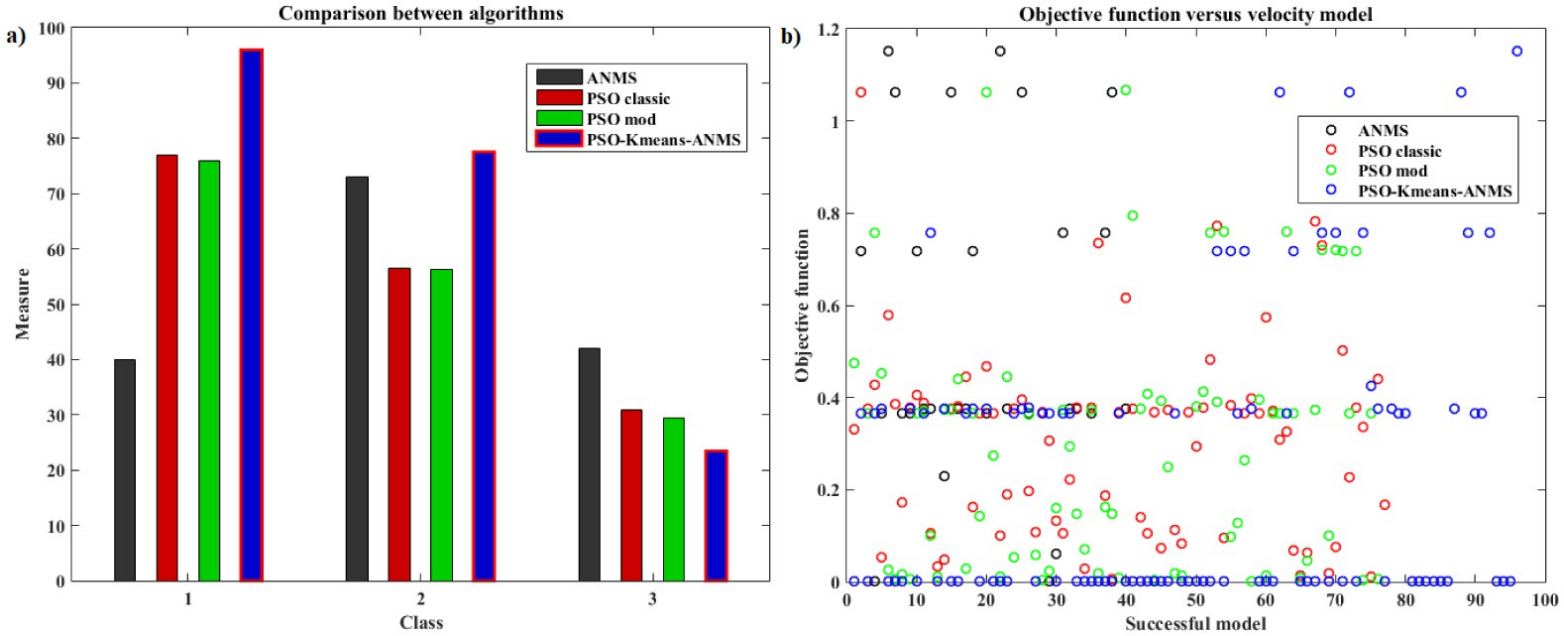
Case 3 (100 × 10). (a) Comparison between algorithms divided into: Class 1, success rates; Class 2, average CPU times and Class 3, average objective function values (*ϕ*(**m**) × 10^2^). (b) Objective function values for successful models.

Finally, Fig 26a shows the graphic summary referring to Case 3 (100 × 10) for the FWI. This summary of each optimization approach has been grouped into value classes. Fig 26b presents the values obtained by the objective function only for the successful models.

Also, based on Fig 26a similar to Case 1 (100 × 20), the PSO-Kmeans-ANMS hybrid optimization algorithm maintained a higher success rate with the objective function value very close to the exact minimum, despite not being able to reduce CPU time. In Fig 26b, the algorithms showed a less uniform distribution and were very close to the exact value, although all misfit values remained in the same range of values. Regarding the CPU time for the PSO (classic or modified), there was a gain when the swarm size was reduced by half, resulting in a significant decrease in its success rate.

**Fig 27:**
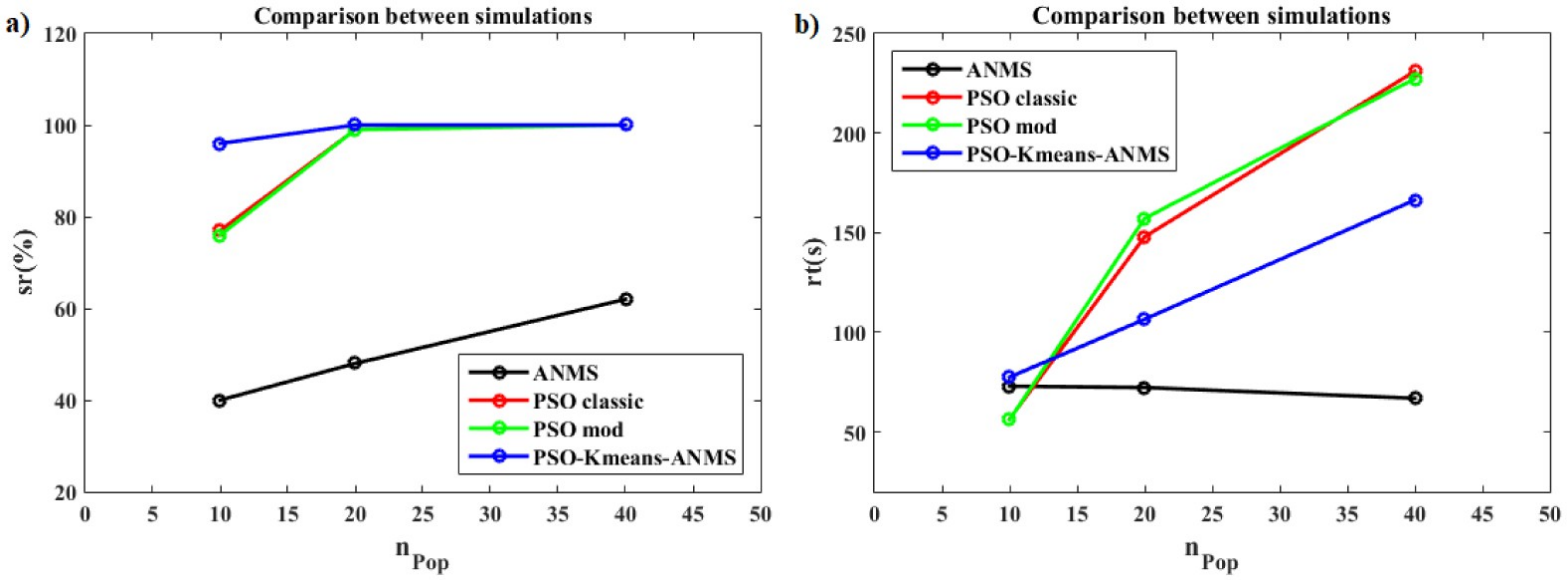
Comparison between simulations for all algorithms and for all cases (1, 2 and 3). (a) success rate, *sr*(%) *versus* population and (b) average execution time, *rt*(s) *versus* population for the ANMS, PSO classic, PSO mod and PSO-Kmeans-ANMS algorithms.

Now let’s do an analysis summarizing the data obtained for the three cases studied in this research, in terms of success rate, *sr*, and average execution time, *rt*, for the optimization algorithms used. Therefore, Fig 27 gives us a graphical view of the data presented in Tables 7, 11 and 13, for Case 1 (100 × 20) with 20 particles, Case 2 (50 × 40) with 40 particles, and Case 3 (100 × 10) with 10 particles, respectively. Cases are identified by the number of particles in the swarm.

From Fig 27a, we can see that the ANMS algorithm presents an increasing and approximately linear behavior for *sr*(%) with the size of the swarm, *n*_*Pop*_. The other algorithms showed an asymptotic increasing behavior. In Fig 27b, the classic PSO, PSO mod, and PSO-Kmeans-ANMS algorithms remained with increasing behavior, being approximately linear for the latter. The ANMS algorithm kept its linear behavior, but practically constant, for the *rt*(s) with the size of the swarm, *n*_*Pop*_. Therefore, based on these graphs, we can conclude that the PSO-Kmeans-ANMS hybrid optimization algorithm presents an increasing and asymptotic behavior for *sr*(%) and linearly increasing behavior for *rt*(s). We also observe that a swarm population equal to 20 constitutes an equilibrium situation for its behavior, that is, at this point, the algorithm has its best performance.

## Conclusion

In the present work, we developed a new two-phase hybrid optimization algorithm based on Derivative-Free Optimization (DFO) and clustering techniques, called PSO-Kmeans-ANMS algorithm. In Phase 1, a modified version of the Particle Swarm Optimization (PSO) global optimizer is used, and at each iteration, the “K-means” clustering algorithm divides the swarm into two groups. When one of the groups becomes dominant, Phase 1 ends. In Phase 2, the proposed optimization approach makes use of a local optimizer, the Nelder-Mead algorithm (ANMS). The Hilbert curve is used to linearize the search space for the optimal solution and divide it into equal regions. This allows to randomly generate the initial population of the “PSO” with a greater diversity for the swarm efficiently and easily. The concept of adaptive parameters together with the use of rebel particles was widely used, offering greater dynamics to the PSO algorithm. Using the same group of particle swarms, the hybrid optimization algorithm was compared with each of the ANMS, classic PSO, and modified PSO algorithms, to analyze their performance in terms of accuracy, efficiency, and robustness. The proposed algorithm was validated through the minimization of 12 test functions, of 2D reference, of the most varied types and complexities; and then applied to a Full Waveform Inversion (FWI) problem. Mathematically, the FWI is an optimization problem, whose objective function (misfit function) requires solving the seismic wave equation in one dimension. We solve the direct modeling of the wave equation by the Finite Difference Method (FDM) using Absorbing Boundary Conditions (ABC) that make the data free of spurious reflections at the domain boundaries. Therefore, FWI is a problem with high computational costs and is difficult to solve.

In general, the PSO-Kmeans-ANMS algorithm proved to be quite efficient and accurate when compared to the other techniques used here. Its robustness was guaranteed by having managed to solve problems of the most varied types. But its main contribution to this research was to have reduced the computation time for the FWI problem. The results found in this research show a good fit between the true parameters and those calculated by numerical simulations.

In the next works, we intend to scale the degree of difficulty of the FWI, with 1D-3D models of the elastic properties much more complex (more parameters) and compare with other algorithms, such as the improved gray wolf optimizer (I-GWO).

## Supporting information

S1 Appendix (PDF)
