# Supplementary material for: A new hybrid optimization approach using PSO, Nelder-Mead Simplex and Kmeans clustering algorithms for 1D Full Waveform Inversion
Source: PLoS One. 2022 Dec 14;17(12):e0277900. doi: 10.1371/journal.pone.0277900 (PMC9750027; doi:10.1371/journal.pone.0277900)
Supplement: S1 Appendix — (PDF) [file pone.0277900.s001.pdf]

Appendix

For the benchmark functions presented in this text, the standard notation can be understood as $\mathbf{X} = x_i = (x_1, \cdots, x_N)$, where N is the number of spatial dimensions of the function.

Ackley Function

The N-dimensional Ackley function, $f(\mathbf{X}) : \Re^N \to \Re$, is given by

$$f(\mathbf{X}) = -A \exp\left(-B\sqrt{\frac{1}{N}\sum_{i=1}^{N} x_i^2}\right) - \exp\left[\frac{1}{N}\sum_{i=1}^{N}\cos\left(Cx_i\right)\right] + A + \exp\left(1\right), \qquad (36)$$

where in this paper were defined for the 2D case, that is, $N = 2$, in which $x_1 = x$ and $x_2 = y$ included in the interval $x_i \in [-32.768, 32.768]$. Recommended variable values are: A = 20, B = 0.2 and C = 2π. The optimal values of the coordinates and the misfit function are $x_1 = 0.0, x_2 = 0.0$ and $f(\mathbf{X}) = 0.0$.

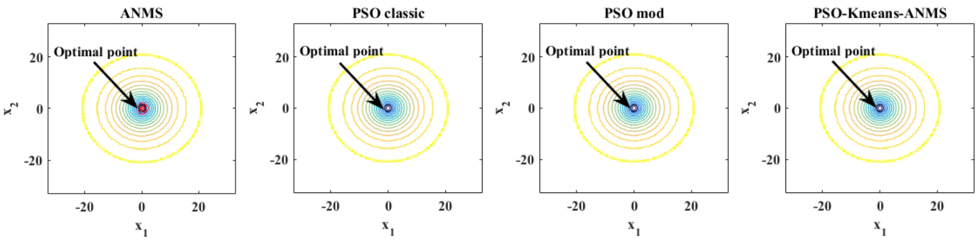

S1 Fig. Simulations details for the Ackley function. The 100 results obtained by the algorithms ANMS, PSO classic, PSO mod and PSO-Kmeans-ANMS for the swarms with 36 particles.

Rastrigin Function

The N-dimensional Rastrigin function, $f(\mathbf{X}) : \Re^N \to \Re$, is given by

$$f(\mathbf{X}) = A \times N + \sum_{i=1}^{N}\left[x_i^2 - A\cos(2\pi x_i)\right], \qquad (37)$$

where in this paper were defined for the 2D case, that is, $N = 2$, in which $x_1 = x$ and $x_2 = y$ included in the interval $x_i \in [-5.12, 5.12]$ and $A = 10$. The optimal values of the coordinates and the misfit function are $x_1 = 0.0, x_2 = 0.0$ and $f(\mathbf{X}) = 0.0$.

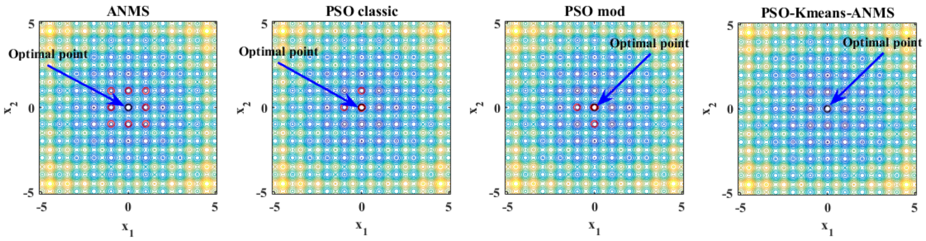

S2 Fig. Simulations details for the Rastrigin function. The 100 results obtained by the algorithms ANMS, PSO classic, PSO mod and PSO-Kmeans-ANMS for the swarms with 36 particles.

Beale Function

The bidimensional Beale function, $f(\mathbf{X}) : \Re^2 \rightarrow \Re$, is given by

$$f(\mathbf{X}) = (1.5 - x_1 + x_1 x_2)^2 + (2.25 - x_1 + x_1 x_2^2)^2 + (2.625 - x_1 + x_1 x_2^3)^2, \qquad (38)$$

in which $x_1 = x$ and $x_2 = y$ included in the interval $x_i \in [-4.5, 4.5]$. The optimal values of the coordinates and the misfit function are $x_1 = 3.0, x_2 = 0.5$ and $f(\mathbf{X}) = 0.0$.

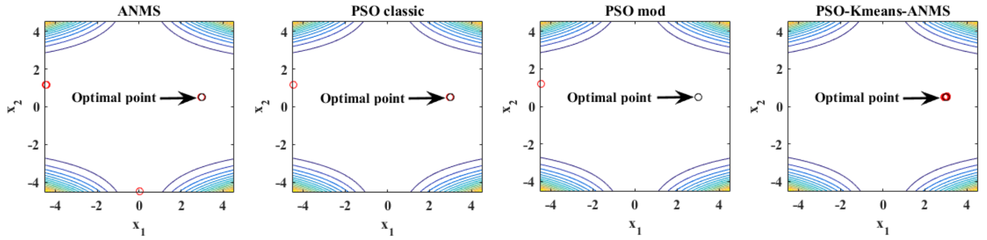

S3 Fig. Simulations details for the Beale function. The 100 results obtained by the algorithms ANMS, PSO classic, PSO mod and PSO-Kmeans-ANMS for the swarms with 36 particles.

Rosenbrock Function

The N-dimensional Rosenbrock function, $f(\mathbf{X}) : \Re^N \rightarrow \Re$, is given by

$$f(\mathbf{X}) = \sum_{i=1}^{N-1} 100 \left[(x_{i+1} - x_i^2)^2 + (x_i - 1)^2 \right], \qquad (39)$$

where in this paper was defined $N = 2$, that is, two dimensions in which $x_1 = x$ and $x_2 = y$ included in the interval $x_i \in [-10, 10]$. The optimal values of the coordinates and the misfit function are $x_1 = 1.0, x_2 = 1.0$ and $f(\mathbf{X}) = 0.0$.

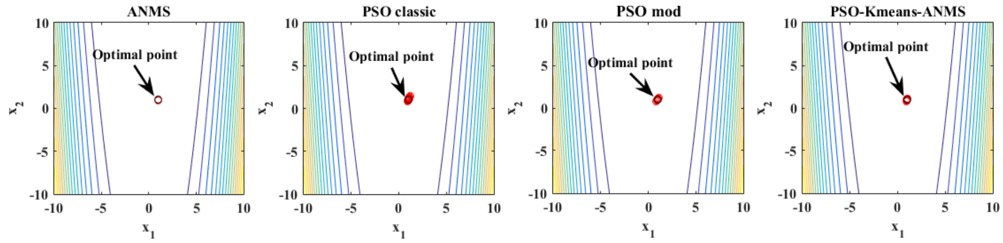

S4 Fig. Simulations details for the Rosenbrock function. The 100 results obtained by the algorithms ANMS, PSO classic, PSO mod and PSO-Kmeans-ANMS for the swarms with 36 particles.

Sphere Function

The N-dimensional Sphere function, $f(\mathbf{X}) : \Re^N \rightarrow \Re$, is given by

$$f(\mathbf{X}) = \sum_{i=1}^{N} x_i^2, \qquad (40)$$

where in this paper were defined for the 2D case, that is, $N = 2$, in which $x_1 = x$ and $x_2 = y$ included in the interval $x_i \in [-5.12, 5.12]$. The optimal values of the coordinates and the misfit function are $x_1 = 0.0, x_2 = 0.0$ and $f(\mathbf{X}) = 0.0$.

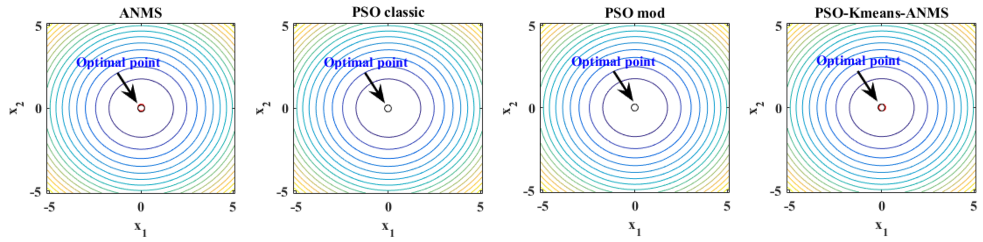

S5 Fig. Simulations details for the Sphere function. The 100 results obtained by the algorithms ANMS, PSO classic, PSO mod and PSO-Kmeans-ANMS for the swarms with 36 particles.

Zakharov Function

The N-dimensional Zakharov function, $f(\mathbf{X}) : \Re^N \rightarrow \Re$, is given by

$$f(\mathbf{X}) = \sum_{i=1}^{N} x_i^2 - \left(\sum_{i=1}^{N} 0.5 i x_i \right)^2 + \left(\sum_{i=1}^{N} 0.5 i x_i \right)^4, \tag{41}$$

where in this paper were defined for the 2D case, that is, $N = 2$, in which $x_1 = x$ and $x_2 = y$ included in the interval $x_i \in [-10, 10]$. The optimal values of the coordinates and the misfit function are $x_1 = 0.0, x_2 = 0.0$ and $f(\mathbf{X}) = 0.0$.

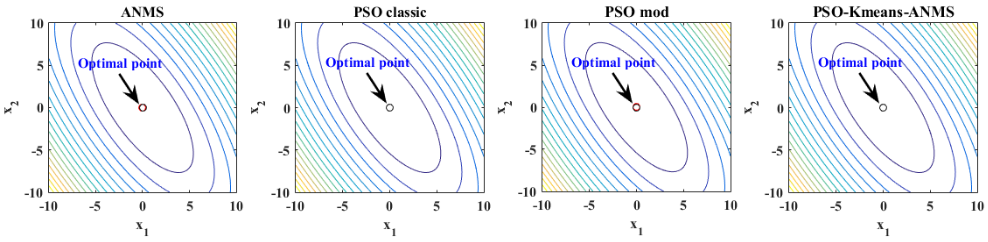

S6 Fig. Simulations details for the Zakharov function. The 100 results obtained by the algorithms ANMS, PSO classic, PSO mod and PSO-Kmeans-ANMS for the swarms with 36 particles.

Michalewicz Function

The N-dimensional Michalewicz function, $f(\mathbf{X}) : \Re^N \rightarrow \Re$, is given by

$$f(\mathbf{X}) = -\sum_{i=1}^{N} \sin(x_i) \sin^{2m}\left(\frac{i x_i^2}{\pi} \right), \tag{42}$$

where in this paper were defined for the 2D case, that is, $N = 2$, in which $x_1 = x$ and $x_2 = y$ included in the interval $x_i \in [0, \pi]$. The optimal values of the coordinates and the misfit function are $x_1 = 2.20, x_2 = 1.57$ and $f(\mathbf{X}) = -1.8013$.

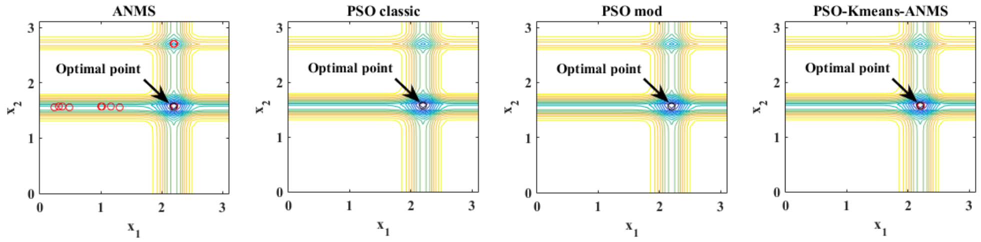

S7 Fig. Simulations details for the Michalewicz function. The 100 results obtained by the algorithms ANMS, PSO classic, PSO mod and PSO-Kmeans-ANMS for the swarms with 36 particles.

Styblinski-Tang Function

The N-dimensional Styblinski-Tang function, $f(\mathbf{X}) : \Re^N \to \Re$, is given by

$$f(\mathbf{X}) = \frac{1}{2}\sum_{i=1}^{N}\left(x_i^4 - 16x_i^2 + 5x_i\right), \tag{43}$$

where in this paper were defined for the 2D case, that is, $N = 2$, in which $x_1 = x$ and $x_2 = y$ included in the interval $x_i \in [-5, 5]$. The optimal values of the coordinates and the misfit function are $x_1 = -2.903534, x_2 = -2.903534$ and $f(\mathbf{X}) = -78.3320$.

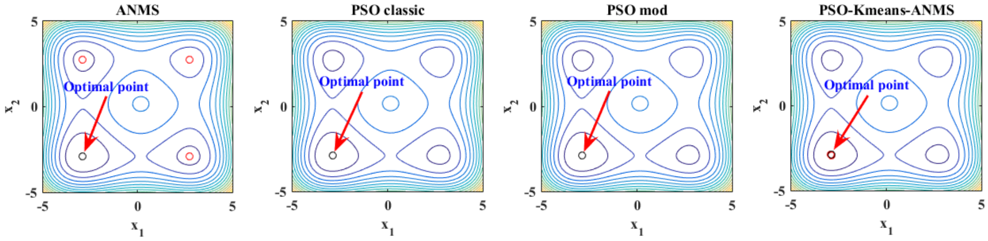

S8 Fig. Simulations details for the Styblinski-Tang function. The 100 results obtained by the algorithms ANMS, PSO classic, PSO mod and PSO-Kmeans-ANMS for the swarms with 36 particles.

F12 Function

The N-dimensional F12 function, $f(\mathbf{X}) : \Re^N \to \Re$, is given by

$$f(\mathbf{X}) = \frac{\pi}{N}\left[10\sin(\pi g_1) + \sum_{i=1}^{N-1}(g_i - 1)^2\left[1 + 10\sin^2(\pi g_{i+1})\right] + (g_N - 1)^2\right] + \sum_{i=1}^{N} u(x_i, 10, 100, 4), \tag{44}$$

$$g_i = 1 + \frac{x_i + 1}{4}, \tag{45}$$

$$u(x_i, a, k, m) = \begin{cases} k(x_i - a)^m, & x_i > a \\ 0, & -a < x_i < a \\ k(-x_i - a)^m, & x_i < -a \end{cases} \tag{46}$$

where in this paper were defined for the 2D case, that is, $N = 2$, in which $x_1 = x$ and $x_2 = y$ included in the interval $x_i \in [-10, 10]$. The optimal values of the coordinates and the misfit function are $x_1 = -1.0, x_2 = -1.0$ and $f(\mathbf{X}) = 0.0$.

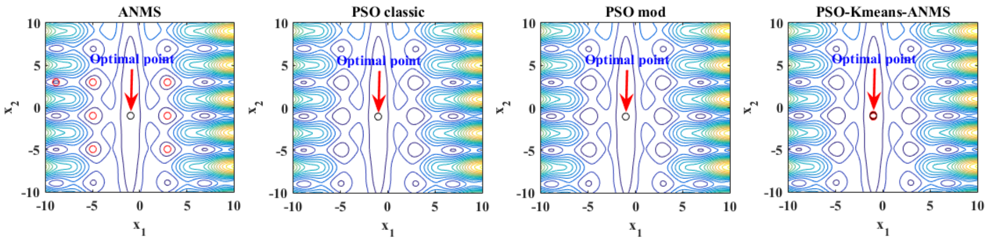

S9 Fig. Simulations details for the F12 function. The 100 results obtained by the algorithms ANMS, PSO classic, PSO mod and PSO-Kmeans-ANMS for the swarms with 36 particles.

F22 Function

The N-dimensional F22 function, $f(\mathbf{X}) : \Re^N \to \Re$, is given by

$$f(\mathbf{X}) = -\sum_{j=1}^{7} \left[(x_i - a_{j,i}) (x_i - a_{j,i})^T + c_j \right]^{-1}, i = 1, \cdots, N \tag{47}$$

with

$$a_{j,i} = \begin{bmatrix} 4 & 1 & 8 & 6 & 3 & 2 & 5 & 8 & 6 & 7 \\ 4 & 1 & 8 & 6 & 7 & 9 & 5 & 1 & 2 & 3.6 \\ 4 & 1 & 8 & 6 & 3 & 2 & 3 & 8 & 6 & 7 \\ 4 & 1 & 8 & 6 & 7 & 9 & 3 & 1 & 2 & 3.6 \end{bmatrix}$$

and $c_j = [0.1, 0.2, 0.2, 0.4, 0.4, 0.6, 0.3, 0.7, 0.5, 0.5]$, where in this paper were defined for the 2D case, that is, $N = 2$, in which $x_1 = x$ and $x_2 = y$ included in the interval $x_i \in [-5, 5]$. The optimal values of the coordinates and the misfit function are $x_1 = 1.02, x_2 = 1.02$ and $f(\mathbf{X}) = -0.524$.

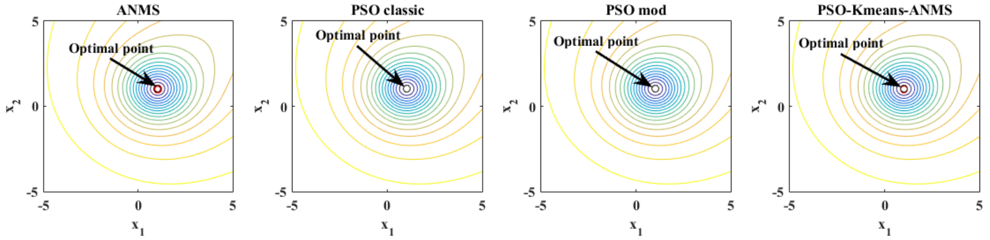

S10 Fig. Simulations details for the F22 function. The 100 results obtained by the algorithms ANMS, PSO classic, PSO mod and PSO-Kmeans-ANMS for the swarms with 36 particles.

F2 Function

The N-dimensional F2 function, $f(\mathbf{X}) : \Re^N \to \Re$, is given by

$$f(\mathbf{X}) = \sum_{i=1}^{N} |x_i| + \prod_{i=1}^{N} |x_i|, \tag{48}$$

where in this paper were defined for the 2D case, that is, $N = 2$, in which $x_1 = x$ and $x_2 = y$ included in the interval $x_i \in [-10, 10]$. The optimal values of the coordinates and the misfit function are $x_1 = 0.0, x_2 = 0.0$ and $f(\mathbf{X}) = 0.0$.

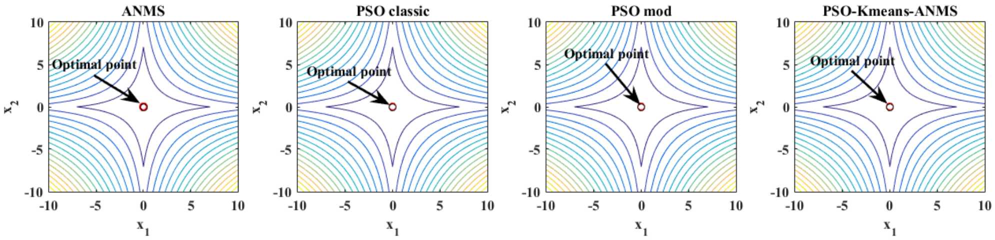

S11 Fig. Simulations details for the F2 function. The 100 results obtained by the algorithms ANMS, PSO classic, PSO mod and PSO-Kmeans-ANMS for the swarms with 36 particles.

Peaks Function

The N-dimensional Peaks function, $f(\mathbf{X}) : \Re^2 \rightarrow \Re$, is given by

$$f(\mathbf{X}) = 3(1-x_1)^2 e^{\left[-x_1^2-(x_2+1)^2\right]} - 10\left(\frac{x_1}{5} - x_1^3 - x_2^5\right) e^{(-x_1^2-x_2^2)} - \frac{1}{3} e^{\left[-(x_1+1)^2-x_2^2\right]}, \quad (49)$$

where $x_1 = x$ and $x_2 = y$ included in the interval $x_i \in [-4, 4]$. The optimal values of the coordinates and the misfit function are $x_1 = 0.2283, x_2 = -1.6255$ and $f(\mathbf{X}) = -6.5511$.

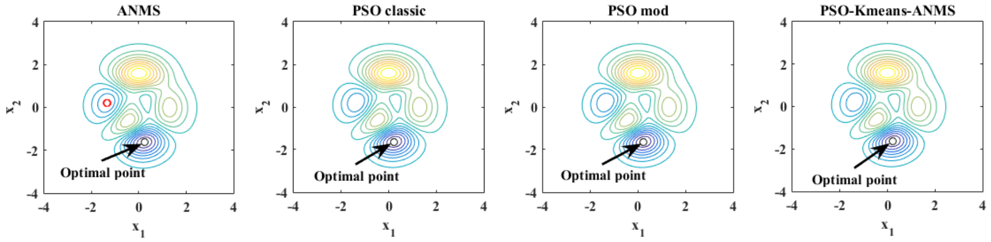

S12 Fig. Simulations details for the Peaks function. The 100 results obtained by the algorithms ANMS, PSO classic, PSO mod and PSO-Kmeans-ANMS for the swarms with 36 particles.

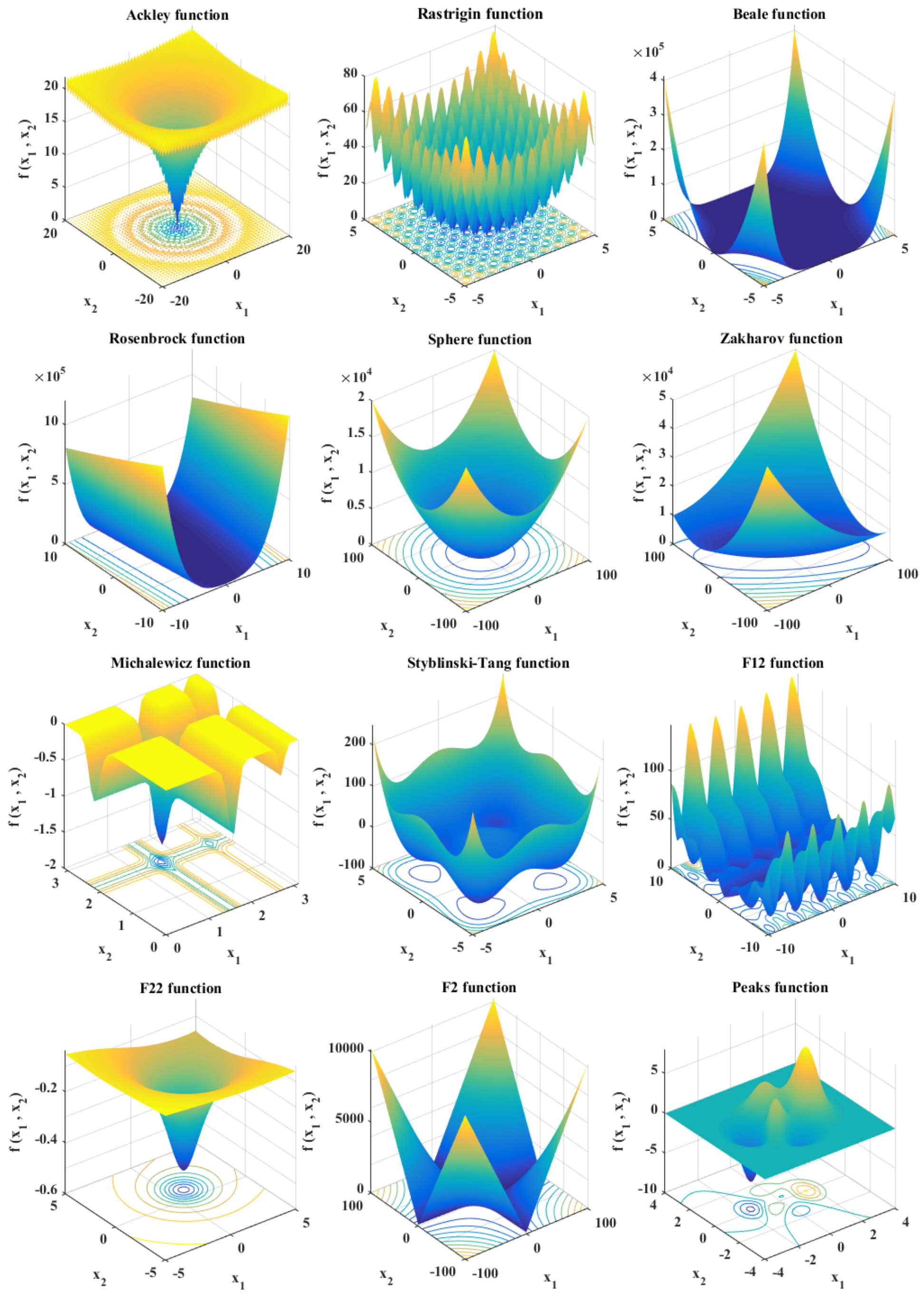

S13 Fig. Benchmark functions for validation of the hybrid algorithm.

S1 Tab. Compilation of all results and best solutions for all algorithms. Data referring to the validation process of the hybrid algorithm. **Abbreviations:** sr(%), success rate; rt(s), average execution time; aes, average number of evaluations; rop, result for the optimal point (approximate).

Functions	Values	Algorithms			
		ANMS	PSO classic	PSO mod	PSO-Kmeans-ANMS
Ackley	sr(%)	100	100	100	100
	rt(s)	7.46E-3	1.58E-1	1.60E-1	2.30E-1<br>5.13E-03
	aes	73	1944	1944	1036<br>56
	rop	x_1 = 1.79E-5<br>x_2 = -1.84E-5<br>$f(\mathbf{X})$ = 7.26E-5	x_1 = -1.18E-4<br>x_2 = 2.56E-5<br>$f(\mathbf{X})$ = 3.42E-4	x_1 = -1.90E-7<br>x_2 = -3.69E-7<br>$f(\mathbf{X})$ = 1.17E-6	x_1 = -1.88E-4<br>x_2 = -3.03E-5<br>$f(\mathbf{X})$ = 5.39E-4
Rastrigin	sr(%)	26	97	98	100
	rt(s)	6.87E-3	1.54E-1	1.55E-1	2.57E-1<br>4.18E-3
	aes	54	1944	1944	1099<br>41
	rop	x_1 = 1.47E-4<br>x_2 = -1.44E-5<br>$f(\mathbf{X})$ = 4.35E-6	x_1 = -2.46E-5<br>x_2 = -3.54E-6<br>$f(\mathbf{X})$ = 1.22E-7	x_1 = 2.87E-9<br>x_2 = -1.84E-8<br>$f(\mathbf{X})$ = 7.11E-14	x_1 = -4.32E-4<br>x_2 = 2.68E-5<br>$f(\mathbf{X})$ = 3.71E-5
Beale	sr(%)	82	86	99	100
	rt(s)	4.92E-3	1.50E-1	1.49E-1	2.20E-1<br>2.61E-3
	aes	48	1944	1944	1011<br>26
	rop	x_1 = 2.99E+0<br>x_2 = 4.99E-1<br>$f(\mathbf{X})$ = 5.87E-6	x_1 = 3.00E+0<br>x_2 = 5.00E-1<br>$f(\mathbf{X})$ = 1.49E-10	x_1 = 3.00E+0<br>x_2 = 5.00E-1<br>$f(\mathbf{X})$ = 8.11E-13	x_1 = 3.00E+0<br>x_2 = 5.01E-1<br>$f(\mathbf{X})$ = 2.33E-6
Rosenbrock	sr(%)	100	99	100	100
	rt(s)	9.21E-3	1.46E-1	1.47E-1	2.22E-1<br>4.03E-3
	aes	116	1944	1944	1014<br>45
	rop	x_1 = 1.00E+0<br>x_2 = 1.00E+0<br>$f(\mathbf{X})$ = 8.05E-7	x_1 = 1.00E+0<br>x_2 = 1.00E+0<br>$f(\mathbf{X})$ = 6.69E-6	x_1 = 1.00E+0<br>x_2 = 1.00E+0<br>$f(\mathbf{X})$ = 1.72E-08	x_1 = 9.99E-1<br>x_2 = 9.98E-1<br>$f(\mathbf{X})$ = 1.00E-6
Sphere	sr(%)	100	100	100	100
	rt(s)	4.28E-3	1.53E-1	1.56E-1	2.24E-1<br>2.61E-3
	aes	33	1944	1944	1013<br>25
	rop	x_1 = -5.55E-4<br>x_2 = -1.33E-3<br>$f(\mathbf{X})$ = 2.08E-6	x_1 = 2.62E-6<br>x_2 = -4.01E-6<br>$f(\mathbf{X})$ = 2.29E-11	x_1 = -1.81E-7<br>x_2 = -2.77E-8<br>$f(\mathbf{X})$ = 3.35E-14	x_1 = -6.04E-4<br>x_2 = 3.29E-4<br>$f(\mathbf{X})$ = 4.73E-7
Zakharov	sr(%)	100	100	100	100
	rt(s)	4.59E-3	1.51E-1	1.58E-1	2.26E-1<br>3.15E-3
	aes	36	1944	1944	1018<br>29
	rop	x_1 = 1.72E-3<br>x_2 = -3.51E-3<br>$f(\mathbf{X})$ = 6.16E-6	x_1 = -2.90E-5<br>x_2 = 5.78E-5<br>$f(\mathbf{X})$ = 1.67E-9	x_1 = -2.13E-7<br>x_2 = 1.32E-8<br>$f(\mathbf{X})$ = 8.52E-14	x_1 = -6.72E-5<br>x_2 = -1.28E-4<br>$f(\mathbf{X})$ = 4.26E-8

Continued on next page

Functions	Values	Algorithms			
		ANMS	PSO classic	PSO mod	PSO-Kmeans-ANMS
Michalewicz	$sr(\%)$	83	100	100	100
	rt(s)	4.13E-3	1.49E-1	1.48E-1	2.28E-1<br>2.74E-3
	aes	36	1944	1944	1056<br>28
	rop	x_1 = 2.20E+0<br>x_2 = 1.57E+0<br>$f(\mathbf{X})$= -1.80E+0	x_1 = 2.20E+0<br>x_2 = 1.57E+0<br>$f(\mathbf{X})$= -1.80E+0	x_1 = 2.20E+0<br>x_2 = 1.57E+0<br>$f(\mathbf{X})$= -1.80E+0	x_1 = 2.20E+0<br>x_2 = 1.57E+0<br>$f(\mathbf{X})$= -1.80E+0
Styblinski	$sr(\%)$	72	100	100	100
	rt(s)	4.46E-3	1.49E-1	1.47E-1	2.20E-01<br>3.13E-3
	aes	40	1944	1944	1030<br>34
	rop	x_1 = -2.90E+0<br>x_2 = -2.90E+0<br>$f(\mathbf{X})$= -7.83E+1	x_1 = -2.90E+0<br>x_2 = -2.90E+0<br>$f(\mathbf{X})$= -7.83E+1	x_1 = -2.90E+0<br>x_2 = -2.90E+0<br>$f(\mathbf{X})$= -7.83E+1	x_1 = -2.90E+0<br>x_2 = -2.90E+0<br>$f(\mathbf{X})$= -7.83E+1
F12	$sr(\%)$	84	100	100	100
	rt(s)	6.19E-3	1.80E-1	1.85E-1	2.51E-1<br>4.11E-3
	aes	39	1944	1944	1026<br>29
	rop	x_1 = -9.99E-1<br>x_2 = -1.00E+0<br>$f(\mathbf{X})$ = 2.93E-6	x_1= -10.00E-1<br>x_2 = -1.00E+0<br>$f(\mathbf{X})$ = 6.13E-10	x_1 = -1.00E+0<br>x_2 = -1.00E+0<br>$f(\mathbf{X})$ = 1.55E-14	x_1= -10.00E-1<br>x_2 = -9.97E-1<br>$f(\mathbf{X})$ = 8.27E-7
F22	$sr(\%)$	100	100	100	100
	rt(s)	5.43E-3	2.00E-1	2.00E-1	2.55E-1<br>3.51E-3
	aes	27	1944	1944	1043<br>21
	rop	x_1 = 1.02E+0<br>x_2 = 1.02E+0<br>$f(\mathbf{X})$ = -5.24E-1	x_1 = 1.02E+0<br>x_2 = 1.02E+0<br>$f(\mathbf{X})$ = -5.24E-1	x_1 = 1.02E+0<br>x_2 = 1.02E+0<br>$f(\mathbf{X})$ = -5.24E-1	x_1 = 1.02E+0<br>x_2 = 1.02E+0<br>$f(\mathbf{X})$ = -5.24E-1
F2	$sr(\%)$	100	100	100	100
	rt(s)	7.62E-3	1.57E-1	1.63E-1	2.33E-1<br>4.68E-3
	aes	61	1944	1944	1016<br>44
	rop	x_1 = 2.31E-5<br>x_2 = -2.01E-5<br>$f(\mathbf{X})$= 4.32E-5	x_1 = -3.46E-6<br>x_2 = 3.09E-5<br>$f(\mathbf{X})$= 3.43E-5	x_1 = 1.60E-7<br>x_2 = 5.85E-8<br>$f(\mathbf{X})$= 2.19E-7	x_1 = -3.70E-5<br>x_2 = -1.41E-4<br>$f(\mathbf{X})$= 1.78E-4
Peaks	$sr(\%)$	80	100	100	100
	rt(s)	4.36E-3	1.48E-1	1.48E-1	2.22E-1<br>3.08E-3
	aes	38	1944	1944	1032<br>33
	rop	x_1 = 2.29E-1<br>x_2 = -1.63E+0<br>$f(\mathbf{X})$= -6.55E+0	x_1 = 2.28E-1<br>x_2 = -1.63E+0<br>$f(\mathbf{X})$= -6.55E+0	x_1 = 2.28E-1<br>x_2 = -1.63E+0<br>$f(\mathbf{X})$= -6.55E+0	x_1 = 2.28E-1<br>x_2 = -1.63E+0<br>$f(\mathbf{X})$= -6.55E+0

End of Table
